# CHERP Regulates the Alternative Splicing of pre-mRNAs in the Nucleus

**DOI:** 10.3390/ijms23052555

**Published:** 2022-02-25

**Authors:** Yasutaka Yamanaka, Takaki Ishizuka, Ken-ichi Fujita, Naoko Fujiwara, Masashi Kurata, Seiji Masuda

**Affiliations:** 1 Division of Integrated Life Sciences, Graduate School of Biostudies, Kyoto University, Kyoto 606-8502, Japan; yamanaka.yasutaka.46z@st.kyoto-u.ac.jp (Y.Y.); ishizuka.takaki.76c@st.kyoto-u.ac.jp (T.I.); kenichi.fujita@fujita-hu.ac.jp (K.-i.F.); fujiwara.naoko.3x@kyoto-u.ac.jp (N.F.); masashikurata_0203@yahoo.ne.jp (M.K.); 2 Division of Gene Expression Mechanism, Institute for Comprehensive Medical Science, Fujita Health University, Toyoake 470-1192, Japan; 3 Department of Food Science and Nutrition, Faculty of Agriculture, Kindai University, Nara 631-8505, Japan

**Keywords:** CHERP, poly(A)^+^ RNA, alternative splicing, U2 snRNP, U2 snRNP related protein, intron retention, cassette exon

## Abstract

Calcium homeostasis endoplasmic reticulum protein (CHERP) is colocalized with the inositol 1,4,5-trisphosphate receptor (IP3R) in the endoplasmic reticulum or perinuclear region, and has been involved in intracellular calcium signaling. Structurally, CHERP carries the nuclear localization signal and arginine/serine-dipeptide repeats, like domain, and interacts with the spliceosome. However, the exact function of CHERP in the nucleus remains unknown. Here, we showed that poly(A)^+^ RNAs accumulated in the nucleus of CHERP-depleted U2OS cells. Our global analysis revealed that CHERP regulated alternative mRNA splicing events by interaction with U2 small nuclear ribonucleoproteins (U2 snRNPs) and U2 snRNP-related proteins. Among the five alternative splicing patterns analyzed, intron retention was the most frequently observed event. This was in accordance with the accumulation of poly(A)^+^ RNAs in the nucleus. Furthermore, intron retention and cassette exon choices were influenced by the strength of the 5′ or 3′ splice site, the branch point site, GC content, and intron length. In addition, CHERP depletion induced anomalies in the cell cycle progression into the M phase, and abnormal cell division. These results suggested that CHERP is involved in the regulation of alternative splicing.

## 1. Introduction

Genetic information is stored in genomic DNA. In higher eukaryotes, immature pre-mRNA is transcribed from genomic DNA by RNA polymerase II and undergoes a series of processing events, including capping, splicing and polyadenylation. The cap is added at the 5′ terminus while the RNA polymerase II pauses. Then, the transcription elongation continues. Introns are sometimes removed concomitantly to elongation in order to generate spliced mRNA. The 3′ terminus becomes polyadenylated once the RNA polymerase II has reached it. Mature mRNAs translocate to the cytosol through the nuclear pore complex to be translated into proteins.

It is important to recognize exons, introns and their boundaries, which are regulated by cis-regulatory elements [1,2,3]. These elements are classified into splicing signals and splicing regulatory elements. Splicing signals, such as the 5′ splice site, the 3′ splice site, the branch point site and the polypyrimidine tract are associated with the spliceosome [4]. Splicing regulatory elements, such as the exonic splicing enhancer (ESE), the exonic splicing silencer (ESS), the intronic splicing enhancer (ISE) and the intronic splicing silencer (ISS) are associated with specific trans-acting factors, like U small nuclear ribonucleoproteins (snRNPs), serine and arginine-rich (SR) proteins and heterogeneous nuclear ribonucleoproteins (hnRNPs) [5].

Splicing requires five U small nuclear RNAs (snRNAs), U1, U2, U4, U5 and U6, and more than 240 proteins [4,6,7]. These components form several sub-complex snRNPs that regulate splicing by binding to specific pre-mRNA sites within the spliceosome. There are two types of mRNA splicing, e.g., constitutive and alternative splicing [8]. The former generates one transcript by excising the introns and combining the exons, whereas the latter generates several transcripts obtained from multiple exon combinations. Thus, alternative splicing produces a variety of mRNAs from a pre-mRNA [9]. It is regulated in tissue, cell and developmental stage-specific manners and is involved in producing the transcript variants from about 90% of human genes with multiple exons. Therefore, this system is essential in humans. The pattern of alternative splicing can be primarily categorized into five groups: cassette exon, alternative 5′ splice site, alternative 3′ splice site, mutually exclusive exons and intron retention [9]. Beside U snRNPs, numerous cis-regulatory elements and trans-acting factors regulate precisely alternative splicing [1]. For example, SR proteins and hnRNPs are implicated in alternative splicing [10,11]. SR proteins are generally thought to activate splicing by binding to ESE and ISE, whereas hnRNPs inhibit splicing by binding to ESS and ISS [2]. Other RNA binding proteins have also been shown to affect alternative splicing [2,12].

Previously, it has been reported that calcium homeostasis endoplasmic reticulum protein (CHERP) is localized in the endoplasmic reticulum and the perinuclear region, and colocalizes with the inositol 1,4,5-trisphosphate receptor (IP3R) [13]. CHERP interacts with a calcium channel of the endoplasmic reticulum, the ryanodine receptor 1 (RyR1), in the skeletal muscle [14], suggesting that it has a function in cell cycle control, cell growth and intracellular calcium signaling by regulating intracellular calcium concentrations [14]. On the other hand, CHERP harbors a nuclear localization signal and multiple structural features related to RNA processing, including an arginine/serine-dipeptide repeat (RS) domain and a glycine-rich motif (G-patch). It also interacts with the spliceosome, especially the U2 snRNP-related factor, implying that CHERP participates in the alternative splicing as an SR-like protein [15]. In addition, CHERP is associated with the apoptosis-linked gene 2 (ALG-2) in nuclear speckles in a Ca^2+^ concentration-dependent manner, and affects the alternative splicing of exon 41 and 42 of the inositol triphosphate receptor type 1 (IP_3_R1) [16]. Recently, it has been found that CHERP is involved in the regulation of splicing by interacting with RNA-binding motif protein 17 (RBM17) and the U2 snRNP-associated SURP domain-containing protein (U2SURP) [17]. However, the precise function of CHERP in the nucleus remains to be determined.

Here, we investigated the function of CHERP in the nucleus and found that CHERP depletion induced an accumulation of poly(A)^+^ RNAs in the nucleus. To evaluate the causes of this nuclear accumulation, we performed RNA sequencing (RNA-seq) analyses, which indicated that the depletion of CHERP induced a dramatic change in alternative splicing, in particular, resulting in an increased retention of introns in transcripts. The properties of the intron retention and other alternative splicing events as well as the cell fate were also analyzed.

## 2. Results

### 2.1. CHERP Is Involved in Splicing in the Nucleus

**Figure 1:**
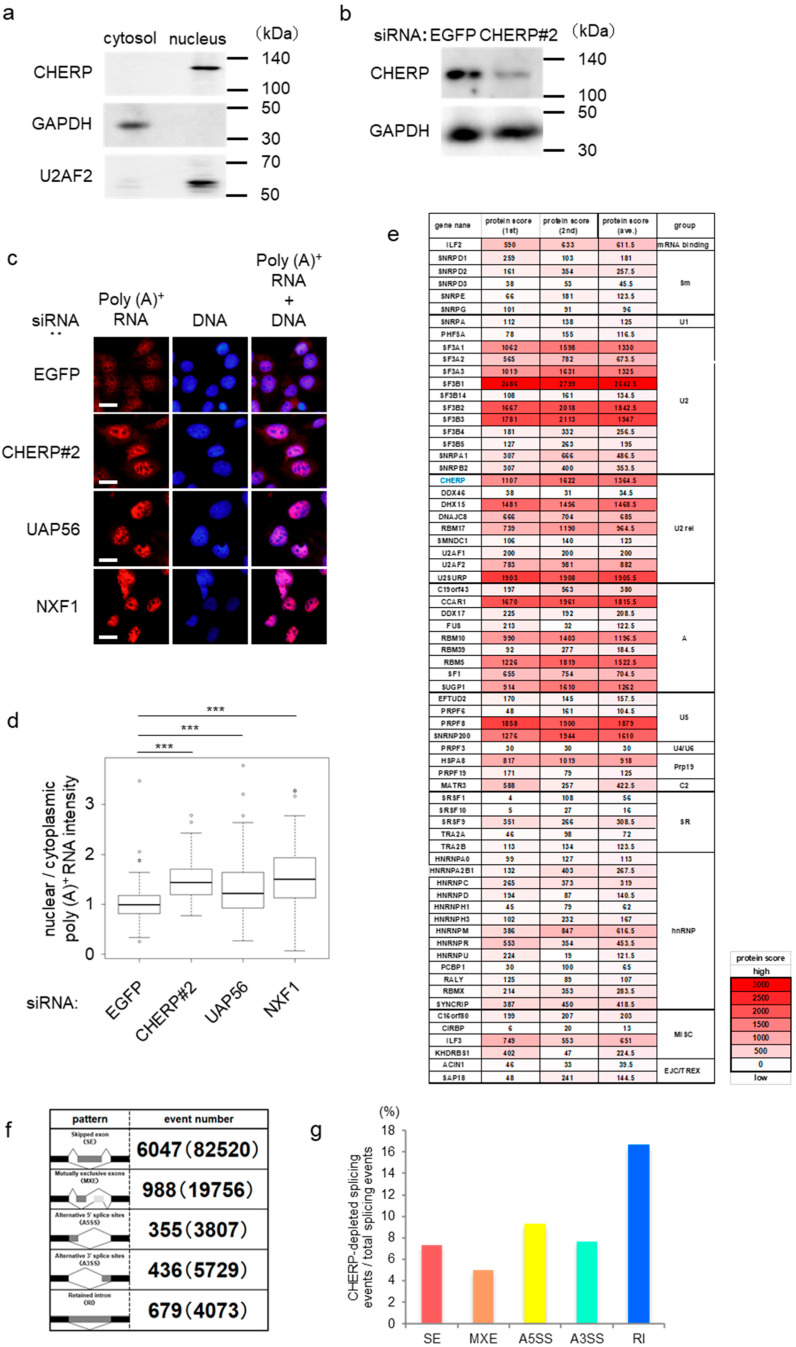
Calcium homeostasis endoplasmic reticulum protein (CHERP) is involved in mRNA metabolism in the nucleus. (**a**) Western blot analysis of CHERP expression in the cytoplasmic and nuclear fractions prepared from U2OS cells. Glyceraldehyde-3-phosphate dehydrogenase (GAPDH) was used as the cytoplasmic marker and U2 small nuclear RNA auxiliary factor 2 (U2AF2) as the nuclear marker. (**b**) Expression of CHERP in U2OS cells transfected with sRNAs against EGFP (control) or CHERP (CHERP#2). GAPDH was used to normalize the sample loading. Samples were analyzed 48 h after transfection. (**c**) Poly(A)^+^ RNA localization analyzed by RNA fluorescent in situ hybridization (RNA-FISH) 48 h after transfection with EGFP (control) or CHERP#2 siRNAs in U2OS cells. Poly(A)^+^ RNAs were detected with Alexa 594 labeled oligo-d(T)_45_ probes. The DNA was stained with 4’, 6-diamidino-2-phenylindole (DAPI). Scale bar, 20 µm. (**d**) Nuclear/cytoplasmic ratios of the poly(A)^+^ RNAs measured in (**c**). The signal intensities of the nucleus and the cytoplasm were quantified (n = 100). Boxes show median (center line) and upper and lower quartiles. Whiskers represent the lowest and highest values. Statistical analyses were performed using one-way analysis of variance (ANOVA) followed by Dunnett’s test. *** *p* < 0.001. (**e**) Splicing-related proteins bound to FLAG-CHERP. Splicing-related proteins with protein score > 0 were shown. CHERP is highlighted with blue letter. All proteins interacting with FLAG-CHERP are shown in Table S1. (**f**) Transcriptome analysis analyzed by rMATS. Cells were transfected with EGFP (control) or CHERP#2 siRNA. RNAs were extracted 48 h after transfection. Alternative splicing events were divided into five splicing patterns by rMATS. The event number in parentheses is the total number of splicing events. Data with false discovery rates (FDR) < 0.05 were significant. (**g**) Frequency of statistically significant changes within each alternative splicing subtype relative to the total number of splicing events in CHERP-depleted cells.

To determine CHERP localization, cellular fractionation was performed and analyzed by Western blot. CHERP was found in the nuclear fraction of U2OS cells (Figure 1a), prompting us to investigate its function in mRNA metabolism. To this aim, we used siRNAs, which induced a marked decrease of CHERP expression (Figure 1b and Figure S1a), and assessed poly(A)^+^ RNA localization (Figure 1c,d and Figure S1b). Poly(A)^+^ RNAs clearly accumulated in the nucleus after the depletion of CHERP to a similar extent as the accumulation induced by silencing the U2 auxiliary factors 65-associated protein (UAP56) or the nuclear RNA export factor 1 (NXF1), which are both involved in mRNA export. These results suggested that CHERP was involved in mRNA processing or nuclear export.

To elucidate the function of CHERP in mRNA nuclear metabolism, we identified proteins interacting with CHERP by immunoprecipitating CHERP from cells stably expressing FLAG-tagged CHERP with an anti-FLAG antibody, and analyzing the interaction partners by liquid chromatography-tandem mass spectrometry (LC-MS/MS). The experiments were performed twice (Figure S1c,d). The proteins detected are listed in Table S1. These were mostly categorized according to functions related to mRNA processing, especially to splicing (Figure 1e and Figure S1e). Among them, components of U2 snRNPs were the most frequently detected. We confirmed that the U2 auxiliary factors U2AF1 (U2AF35) and U2AF2 (U2AF65) interacted with CHERP by Western blot (Figure S1f). These results implicated that CHERP likely had a role in an early step of splicing. To evaluate the exact influence of CHERP on splicing events, transcriptome analyses of CHERP-depleted U2OS cells were performed (Figure 1f,g and Figure S1g,h). We classified the alternative splicing events into five categories using rMATS. The frequency of splicing changes was measured, and an event was considered significant for false discovery rate (FDR) < 0.05. CHERP depletion elicited an alteration of all alternative splicing events, with retained introns being the most frequent.

### 2.2. Identification and Characterization of Intron Retention after CHERP Depletion

**Figure 2:**
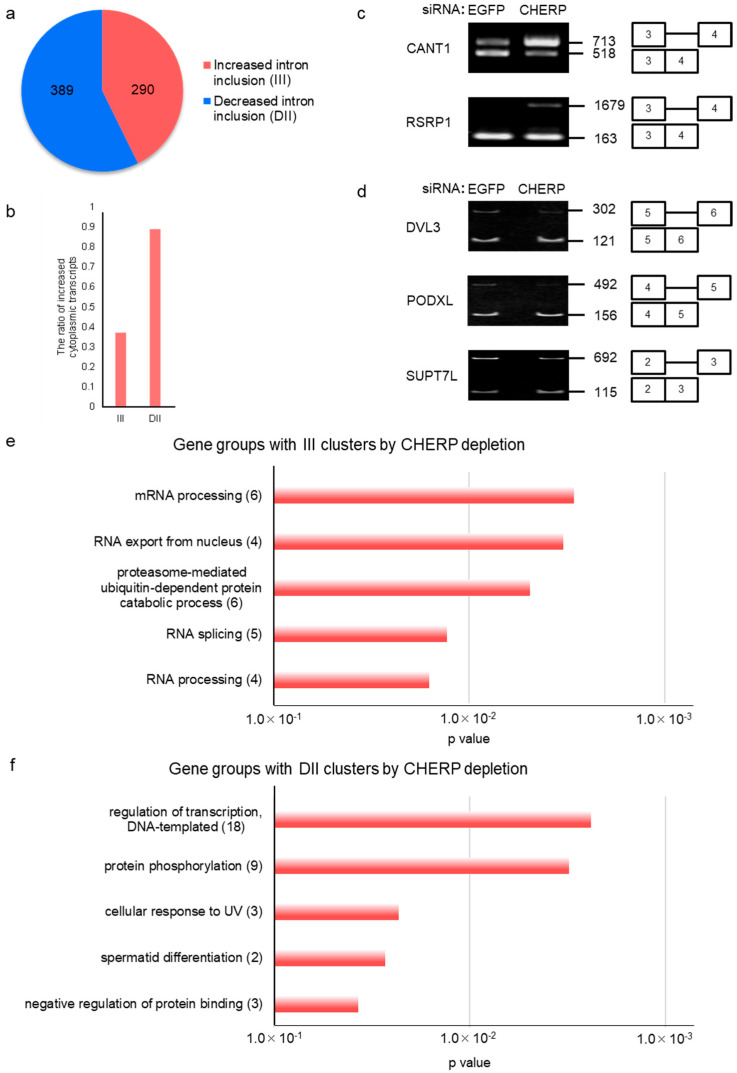
CHERP regulates transcripts with increased intron inclusions (III) and decreased intron inclusions (DII). (**a**) Number of increased and decreased intron inclusion events after CHERP depletion. Events with FDR < 0.05 were considered statistically significant. (**b**) Cytoplasmic/nuclear ratios of transcripts with III or DII after CHERP depletion. Events with FDR < 0.05 were considered statistically significant. (**c**,**d**) Validation of (**c**) III or (**d**) DII induced by CHERP depletion by reverse transcription-polymerase chain reaction (RT-PCR). (**e**,**f**) Gene ontology (GO) groups enriched in transcripts with III (**e**) or DII (**f**) after CHERP depletion are shown. FDR < 0.05, inclusion or skipping count ≥ 10, and IncLevelDifference ≤ –0.1 for RNA sequencing (RNA-Seq) data was considered statistically significant.

To investigate the characteristics of introns retained in response to CHERP depletion, we considered separately increased intron inclusions (III) and decreased intron inclusions (DII) and found 290 III and 389 DII cases for FDR < 0.05 (Figure 1e and Figure 2a), respectively. We measured the levels of mRNAs with III and DII events in the cytoplasm. As expected, CHERP depletion induced a tendency toward a decrease of the levels of transcripts with III, whereas the amounts of mRNAs with DII were clearly increased in the cytoplasm of CHERP-depleted cells (Figure 2b and Figure S2a). Next, we validated the results regarding retained introns. To this aim, reverse transcription-polymerase chain reactions (RT-PCRs) were performed using a series of polymerase chain reaction (PCR) primer pairs targeting two adjacent constitutive exons of a selected intron. III and DII were observed in the CHERP-depleted condition (Figure 2c,d). The introns in calcium-activated nucleotidase 1 (CANT1) and arginine and serine rich protein 1 (RSRP1), which typically are III, were increased, and introns of disheveled segment polarity protein 3 (DVL3), like podocalyxin (PODXL) and STAGA complex 65 subunit gamma (SUPT7L), which are usually DII, were reduced. Gene ontology (GO) analysis of transcripts with III and DII in CHERP-depleted cells are shown in Figure 2e,f. The III-containing transcripts in CHERP-depleted cells were related to RNA metabolism, whereas a variety of gene functions were affected by DII. GOs-enriched transcripts with retained introns were similar to those enriched in DII-containing mRNAs, rather than to those with III. Thus, poly(A)^+^ RNAs accumulated in the nucleus of CHERP-depleted cells seemed to be linked to III events. Moreover, dividing retained intron events into III and DII allowed identification of the poly(A)^+^ RNA that accumulated.

### 2.3. CHERP Regulates Intron Retention in the Target Transcripts

**Figure 3:**
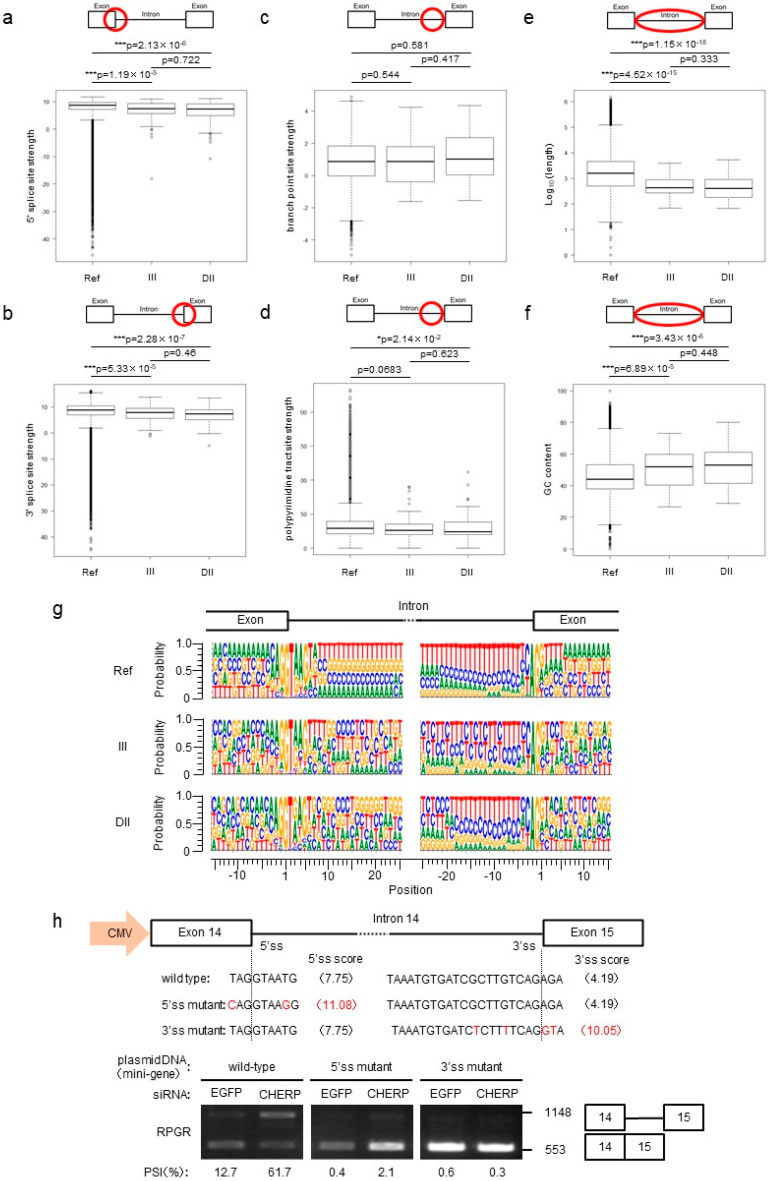
Characteristics of III and DII induced by CHERP depletion. (**a**,**b**) 5′ and 3′ splice site scores after CHERP depletion, calculated by MaxEntScan. (**c**) Branch point site scores after CHERP depletion, calculated by SVM-BPfinder. (**d**) Polypyrimidine tract site scores after CHERP depletion, calculated by SVM-BPfinder. (**e**,**f**) Length and GC content of CHERP-targeted introns, analyzed by BedTools. (**a**–**f**) FDR < 0.05, inclusion or skipping count ≥ 10, and IncLevelDifference ≤ –0.1 (III) or IncLevelDifference ≥ 0.1 (DII) were considered significantly different. All introns were used as reference introns. For statistical analysis, a Wilcoxon rank sum test was used to calculate *p*-values. * *p* < 0.05, *** *p* < 0.001. (**g**) Nucleotide sequences of exon–intron boundaries in III, DII and references, estimated with BedTools. (**h**) Splicing assay using retinitis pigmentosa GTPase regulator (RPGR) minigenes containing CHERP target intron. Red letters indicated bases and scores that introduce mutation strengthening 5’ or 3’ splice site.

To determine which mechanisms were triggered by CHERP to alter splicing events, we investigated the properties of introns. In both III and DII, the 5′ and 3′ splice site scores of introns affected by CHERP depletion were weaker than those obtained for the reference genes (Figure 3a,b). The branch point site scores were not significantly changed, and the polypyrimidine tract site scores in III were weaker than those of the reference genes (Figure 3c,d). Furthermore, the length of introns affected by CHERP depletion was significantly shorter than that of the reference genes (Figure 3e). The DII regulated by CHERP had a higher GC content in introns than that in reference genes (Figure 3f). Short introns with high GC content have been associated with intron inclusion events [18]. As splicing efficiency is based on the splice site, branch point site and polypyrimidine tract site scores, we analyzed the nucleotides in CHERP-targeted 5′ and 3′splice sites (Figure 3g). Both sides of the intronic sequences in the reference genes were rich in thymine residues. In contrast, III and DII induced by CHERP depletion were slightly different from those of the reference genes. To examine whether the weak splice sites were targeted by CHERP, we constructed a retinitis pigmentosa GTPase regulator (RPGR) minigene as a target for CHERP. Additionally, mutant minigenes, containing 5′ and 3′ splice site mutations that rendered the splice sites stronger, were generated. Plasmids with wild-type or mutant minigenes were transfected to cells, and transgene products were analyzed by RT-PCR. The splicing in the wild-type minigenes, but not in the mutant minigenes, was dependent on CHERP (Figure 3h). These data suggested that CHERP functioned as a regulator involved in the exact splice site selection.

### 2.4. Identification and Characterization of Cassette Exons Regulated by CHERP

**Figure 4:**
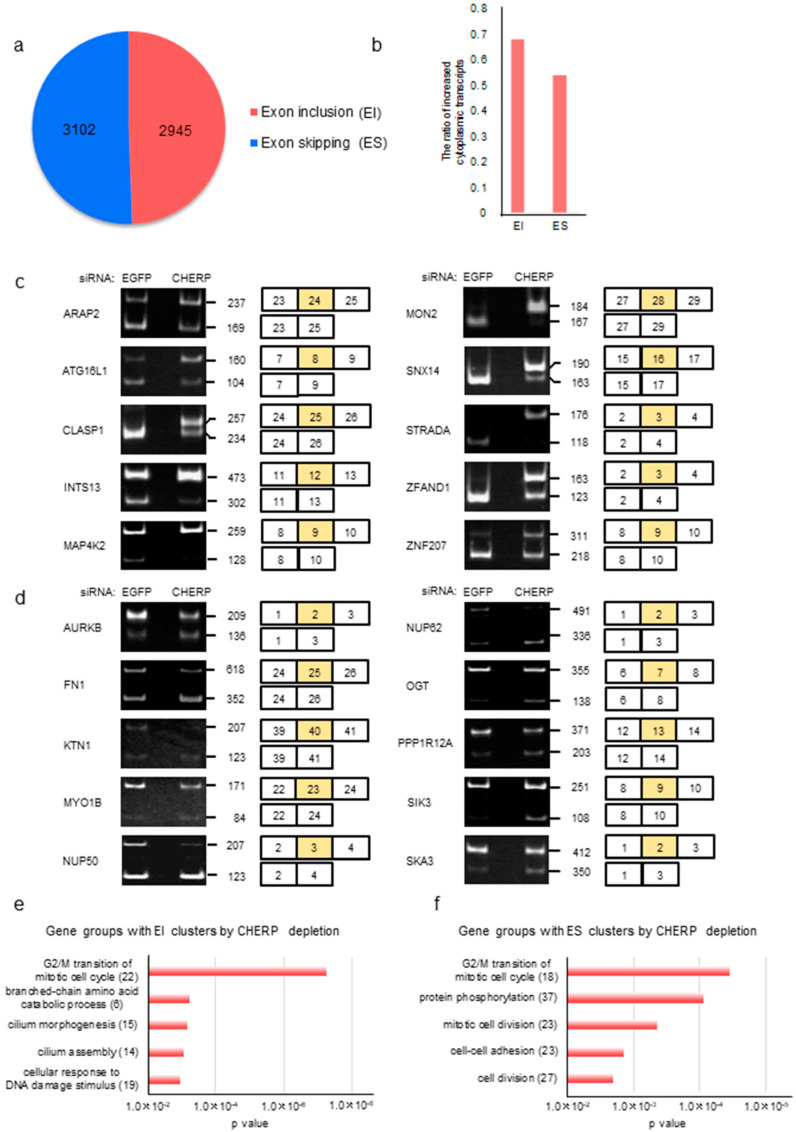
CHERP regulates transcripts with exon inclusion (EI) or exon skipping (ES). (**a**) Number of exon inclusion and skipping events after CHERP depletion. Target exons with FDR < 0.05 were considered statistically significant. (**b**) Cytoplasmic/nuclear expression ratios of mRNAs with EI or ES after CHERP depletion. Target exons with FDR < 0.05 were considered statistically significant. (**c**,**d**) RT-PCR validation of (**c**) EI or (**d**) ES induced by CHERP depletion. (**e**,**f**) GO groups enriched in transcripts with EI (**e**) or ES (**f**) induced by CHERP depletion are shown. FDR < 0.05, inclusion or skipping count ≥10, and IncLevelDifference ≤ –0.1 (EI) or ≥0.1 (ES) for RNA-Seq data were considered statistically significant.

The number of altered exon inclusions (EI) and exon skipping (ES) in cassette exons regulated by CHERP were counted. We found 2945 cases of EI and 3102 instances of ES with FDR < 0.05 (Figure 1e and Figure 4a), implicating that CHERP did not selectively affect EI or ES. EI tended to be increased in cytoplasmic target transcripts, whereas ES was not dramatically affected (Figure 4b and Figure S4a). To validate the cytoplasmic expression of alternatively spliced transcripts, we designed PCR primer pairs located in two exons adjacent to the target exon. RT-PCR analyses clearly showed EI and ES in CHERP-depleted cells, suggesting that transcripts with EI and ES were successfully exported to the cytoplasm (Figure 4c,d and Figure S4a). The transcripts with EI after CHERP depletion were enriched in the GO cell cycle, whereas those with ES were linked to cell cycle or division (Figure 4e,f and Figure S4b).

### 2.5. CHERP Regulates Cassette Exon Inclusion and Skipping in Target Genes

**Figure 5:**
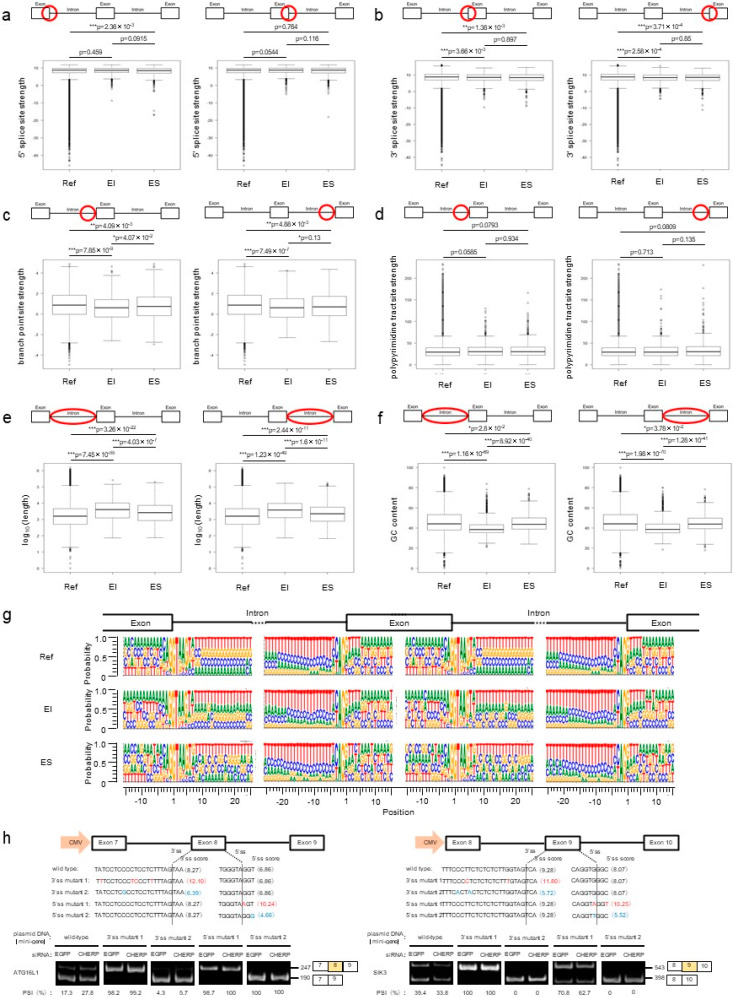
Characteristics of EI and ES induced by CHERP depletion. (**a**,**b**) 5′ and 3′ splice site scores after CHERP depletion, calculated by MaxEntScan. (**c**) Branch point site scores after CHERP depletion, calculated by SVM-BPfinder. (**d**) Polypyrimidine tract site scores after CHERP depletion, calculated by SVM-BPfinder. (**e**,**f**) Length and GC content of CHERP-targeted introns, analyzed by BedTools. (**a**–**f**) FDR < 0.05, inclusion or skipping count ≥ 10, and IncLevelDifference ≤ –0.1 (EI) or IncLevelDifference ≥0.1 (ES) were considered significantly different. For statistical analysis, Wilcoxon rank sum test was used to calculate *p*-value. * *p* < 0.05, ** *p* < 0.01, *** *p* < 0.001. (**g**) Nucleotide sequences of exon–intron boundaries in EI, ES and references, estimated by BedTools. (**h**) Splicing assay using autophagy related 16 like 1 (ATG16L1) and serine/threonine-protein kinase 3 (SIK3) minigenes containing CHERP target introns. Red letters indicated bases and scores that introduce mutation strengthening 5’ or 3’ splice site. Blue letters indicated bases and scores that introduce mutation weakening 5’ or 3’ splice site.

To elucidate the specificity of CHERP-regulated cassette exons, we performed a correlation analysis between the splice site properties and CHERP-dependent or -independent genes. In EI and ES, the 5′ splice sites in introns upstream and downstream of targeted exons were not significantly different from those of reference genes, except for 5′ splice sites in introns upstream of ES (Figure 5a). By contrast, 3′ splice sites in introns upstream and downstream of targeted exons were weaker than those of reference genes (Figure 5b), implicating that U2 snRNP recruitment to 3′ splice sites of upstream and downstream introns might be reduced. Branch point site scores were significantly weaker for upstream and downstream introns targeted by CHERP. Polypyrimidine tract site scores were not altered compared with those of reference genes (Figure 5c,d). These results suggest that the environment around CHERP-targeted exons was less likely to be spliced than reference exons. CHERP-regulated introns upstream and downstream from targeted exons were significantly longer than those of reference genes (Figure 5e). Furthermore, in introns upstream and downstream from targeted exons, the GC content was lower in EI and ES than that of reference genes (Figure 5f), suggesting that exons surrounded by longer introns with lower GC content tended to be targets of CHERP in skipped exon (SE). These intron profiles were different from those of III and DII (compare with Figure 3e,f). Then, we calculated the nucleotide usage of 5′ and 3′ splice sites for introns upstream and downstream from CHERP-targeted exons. CHERP-targeted nucleotide sequences were not much different from that in reference genes, although 5′ and 3′ splice site scores seemed lower (Figure 5g). Therefore, a slight difference in nucleotides compared with reference genes might affect CHERP-dependent EI and ES. To examine this hypothesis, we constructed autophagy related 16 like 1 (ATG16L1) and serine/threonine-protein kinase 3 (SIK3) minigenes for EI and ES analysis, respectively. Mutations leading to stronger or weaker splice sites were introduced to 5′ or 3′ splice sites. When strengthening splice site mutations were introduced in the 3′ splice sites, the target exon was fully recognized independently of CHERP depletion (Figure 5h). By contrast, weakening mutations in the 3′ splice sites of the target exons led to the exons being fully skipped, regardless of CHERP depletion. Similar results were obtained with mutations introduced in the 5′ splice sites. Among these mutations, four mutants consisted of a single nucleotide change, supporting the hypothesis that nucleotide sequences around the 5′ and 3′ sites of target exons were sensitive to CHERP.

### 2.6. CHERP Regulates Cell Survival and Cell Death

**Figure 6:**
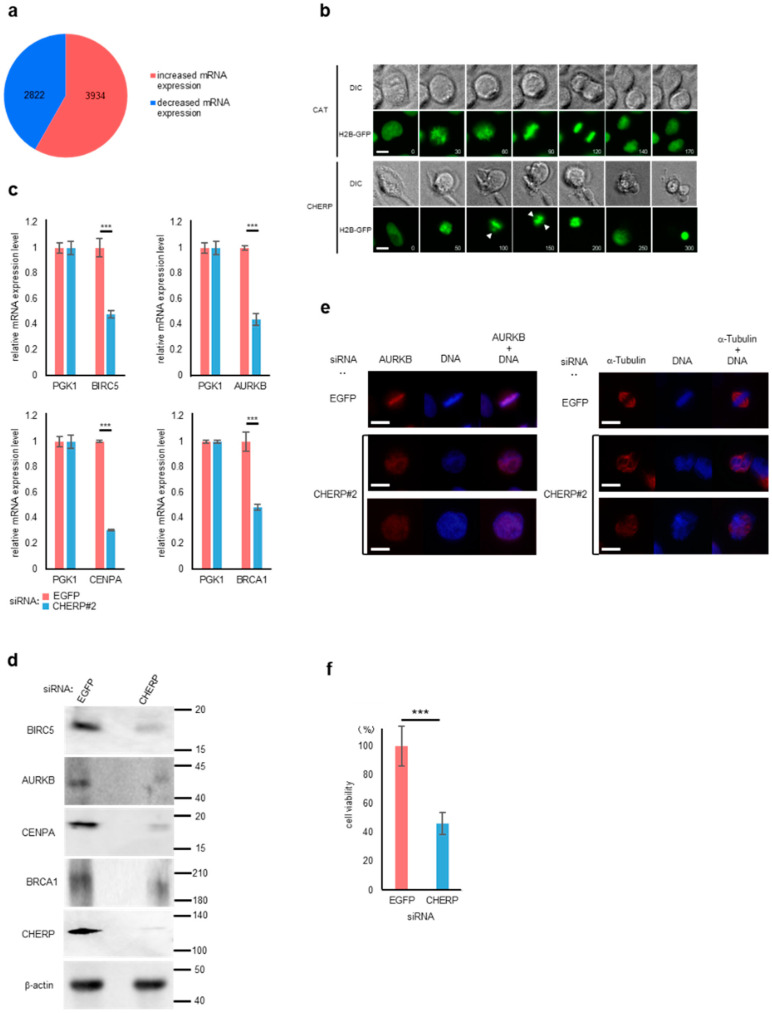
CHERP is involved in cell survival and cell death through the expression of cell cycle regulators. (**a**) Number of increased and decreased mRNA expression events after CHERP depletion. Events with FDR < 0.05 were considered statistically significant. (**b**) Live-cell imaging pictures of chloramphenicol acetyltransferase (CAT: control) and CHERP-depleted (CHERP#2) U2OS cells stably expressing histone 2B-green fluorescent proteins (H2B-GFP). The numbers at the bottom right side of the pictures indicates the time (min) from nuclear envelope breakdown (NEBD). The arrowhead indicates the misaligned chromosome. Scale bar, 10 μm. (**c**) Cytoplasmic expression of mRNAs under CHERP depletion. The mRNA expression of factors regulating the cell cycle (the Survivin/baculoviral IAP repeat containing 5 [BIRC5]), cell division (aurora kinase B [AURKB] and centromere protein A [CENPA]), and apoptosis (breast cancer type 1 [BRCA1]), was measured by reverse transcription-quantitative PCR (RT-qPCR) and normalized to the levels of phosphoglycerate kinase 1 (PGK1) mRNA. The bars indicated relative mRNA expression of samples treated with siRNA against EGFP (red) and CHERP#2 (blue). Statistical analyses were performed using one-way analysis of variance (ANOVA) followed by Dunnett’s test. *** *p* < 0.001. (**d**) The cytoplasmic expression of BIRC5, AURKB, CENPA and BRCA1 proteins after CHERP depletion, measured by Western blot. Protein levels were normalized to β-actin levels. (**e**) Effect of CHERP depletion on AURKB and α-Tubulin localization. Scale bar, 10 μm. (**f**) Effect of CHERP depletion on cell viability, measured by MTT assay. Statistical analyses were performed using Student’s t-test. *** *p* < 0.001.

The GO analysis revealed an enrichment of cell cycle genes in transcripts with EI induced by CHERP depletion, and those with ES were enriched in cell cycle or division, implying that CHERP played a role in cell cycle progression and cell viability. To investigate the role of CHERP in cell function, the cytoplasmic mRNA transcriptome was analyzed in cells with or without CHERP depletion. CHERP depletion induced the increase of the expression of 3934 transcripts and a decreased expression of 2822 mRNAs (Figure 6a). To determine the cellular function of CHERP depletion, the GO analysis of transcripts with an expression increased more than 1.5 fold and less than 0.67 fold is shown (Figure S6a,b). Several transport functions were enriched, whereas the representation of cell cycle-related genes was reduced when CHERP was depleted. We then investigated whether the function of CHERP was associated with cell cycle or cell division. U2OS cells stably expressing histone 2B-green fluorescent protein (H2B-GFP) were used to analyze cell division by live cell imaging (Figure 6b). In CHERP-depleted cells, centromeres were not well aligned, and the time of mitosis was often delayed during the prometaphase. aurora kinase B (AURKB) has a key role in the alignment of chromosomes at prometaphase and AURKB mRNA expression was markedly reduced in CHERP-depleted cells (Figure 6c). CHERP depletion also reduced the cytoplasmic expression of the Survivin/baculoviral IAP repeat containing 5 (BIRC5), AURKB, centromere protein A (CENPA) and breast cancer type 1 (BRCA1) mRNAs, which function in the progression of the M phase, prompting us to investigate the corresponding protein expression. BIRC5, AURKB, CENPA and BRCA1 protein levels were reduced in CHERP-depleted cells (Figure 6d). Moreover, CHERP depletion not only resulted in a weak expression of AURKB, but also in its aberrant distribution, as shown by immunostaining (Figure 6e). CHERP depletion also led to the aberrant distribution of α-Tubulin. These mislocalizations might be the cause of the delay of the M phase progression. The viability of CHERP-depleted cells was significantly reduced (Figure 6f), suggesting that CHERP was indispensable for cellular function, likely by regulating alternative splicing.

## 3. Discussion

Here, the function of CHERP in mRNA metabolism was investigated. RNA fluorescent in situ hybridization (RNA-FISH) analyses revealed that poly(A)^+^ RNAs accumulated in the nucleus of CHERP-depleted cells, suggesting that CHERP was involved in mRNA metabolism in the nucleus [19,20]. We then performed LC-MS/MS analyses to assess CHERP functions by identifying its interaction partners. CHERP interacted strongly with U2 snRNP components, such as spliceosome factor 3b subunits SF3B1, SF3B2 and SF3B3, and U2-related proteins, such as U2AF2 and U2SURP, indicating that CHERP participated in mRNA splicing [21,22]. Our results are consistent with the results of Lin-Moshier et al., who used HEK293 cells stably expressing GFP-CHERP [15]. De Maio et al. also showed, by immunoprecipitation, that CHERP forms a complex with RBM17 and U2SURP to induce splicing changes [17]. RBM17 and U2SURP were detected as a CHERP interaction partner in our LC-MS/MS experiments, indicating that our data were solid.

Poly(A)^+^ RNAs accumulated in the nucleus of CHERP-depleted cells and CHERP was associated with U2 snRNP components. This prompted us to investigate CHERP’s role in mRNA splicing using next generation sequencing (NGS) and rMATS. Among alternative splicing events, retained introns were the most frequently observed, which was consistent with our observation that CHERP depletion induced poly(A)^+^ RNA accumulation in the nucleus [18,23]. Although the transcriptome analysis revealed that all types of alternative splicing were affected by CHERP depletion, which was in agreement with the De Maio et al. paper, a detailed analysis showed that CHERP depletion caused retained introns more frequently, although this was not observed by De Maio et al. [17]. This discrepancy might be due to the different cell types used in both analyses. This possibility is supported by the recent findings that CHERP depletion in Hela cells frequently caused retained intron events [24] and implies that CHERP regulated the alternative splicing in a cell-specific manner. In our analysis, weak or suboptimal splice sites, high GC content in introns, and short intron lengths were important and common features of intron retention. Interestingly, the depletion of another RNA binding protein, SON, also resulted in the nuclear retention of introns with the same features [23,25]. It has been reported that CHERP forms a complex with RBM17, an RNA-binding protein, and U2SURP, an SR protein [17], suggesting that CHERP does not function as a core splicing factor but rather as a modulating factor [26,27].

Alternative splicing is, importantly, regulated by the sequences of 5′ and 3′ splicing sites and their combinations [6,7,23,28,29,30]. In addition, cis-acting RNA elements are involved in splicing regulation [31,32,33,34,35]. Cis-acting RNA elements often become the target sites of trans-acting factors [36,37,38,39,40,41,42,43]. The status and positional relations of these signals have a significant effect on splicing. Thus, alternative splicing is intricately regulated. We have shown that the GC content is low in the upstream and downstream introns of EI induced by CHERP depletion. This seemed to be different from the data obtained for CHERP-regulated retained introns, which had high GC contents. Interestingly, it has been reported that CHERP binds to specific adenine-rich sequences on exons and causes splicing changes [44]. Thus, CHERP might participate in the regulation of EI and ES by binding to adenine-rich sequences. 

The depletion of CHERP induced alternative splicing changes, in particular of factors promoting the entrance into the M phase, including BIRC5, AURKB, CENPA, and BRCA1, which results in the reduced cytoplasmic expression of these factors [45,46,47]. Our data showed that the cellular expression of these factors was reduced, and AURKB and α-Tubulin were totally mislocalized, implying that CHERP targets genes involved in the progression into the M phase. The implication of the CHERP association with RBM17 and U2SURP in the regulation of splicing events, such as RI and SE, will be investigated next by depleting these factors to uncover a more specific role of CHERP in splicing.

## 4. Materials and Methods

### 4.1. Reagents and Instruments

The following commercial chemicals were used: Dulbecco’s Modified Eagle’s Medium (DMEM) containing high glucose, 4’, 6-diamidino-2-phenylindole (DAPI), and SuperSep™ Ace (FUJIFILM Wako, Osaka, Japan), Fetal bovine serum (FBS), ULTRAhyb ^TM^ -Oligo Hybridization Buffer, and cell culture dish and plate (Thermo Fisher Scientific, Waltham, MA, USA), Alexa Fluor 594-labeled oligo-d(T)_45_ probe (Molecular Probes, Eugene, OR), Lipofectamine 2000 (Invitrogen, Carlsbad, CA, USA), bovine serum albumin, and 3-(4,5-dimethylthiazol-2-yl)-2,5-diphenyltetrazolium bromide, yellow tetrazole (MTT) (Sigma-Aldrich, St. Louis, MO, USA), the chemiluminescent reagent (Merck Millipore, Burlington, MA, USA), Rever Tra Ace^®^, KOD Fx Neo, THUNDERBIRD^®^ SYBR qPCR Mix, and restriction enzymes (Toyobo, Osaka, Japan), RQ1 DNase-Free DNase (Promega, Madison, WI, USA), restriction enzymes (Takara BIO, Kyoto, Japan), PlusOne^TM^ Silver Staining Kit Protein (GE Healthcare, Chicago, IL, USA), polyvinylidene difluoride (PVDF) membrane (Pall, Port, Washington, NY, USA), ECL substrate (Bio-Rad, Richmond, VA, USA) and trypsin-ethylenediaminetetraacetic acid (EDTA), doxycycline, hygromycin, Sepasol^®^ RNA I Super G, and other general reagents (Nacalai tesque, Kyoto, Japan).

### 4.2. Plasmid DNAs, siRNAs and Oligo DNA Primers

The plasmid DNAs, siRNAs and oligo DNA primers used in this study are listed in Tables S2–S4. We used pcDNA5/FRT/TO and siRNAs from Invitrogen, and siRNAs and oligo DNA primers from Integrated DNA Technologies (Coralville, IA, USA).

### 4.3. Antibodies

The following commercial antibodies were used: anti-glyceraldehyde 3-phosphate dehydrogenase (GAPDH) mouse polyclonal antibody (FUJIFILM Wako), anti-β-actin mouse monoclonal antibody, anti-FLAG M2 mouse monoclonal antibody, anti-CENPA rabbit polyclonal antibody (Sigma-Aldrich), anti-BIRC5 rabbit polyclonal antibody (Abnova, Taipei, Taiwan), anti-AURKB mouse monoclonal antibody (BD Biosciences, Franklin Lakes, NJ, USA), anti-BRCA1 rabbit polyclonal antibody (Santa Cruz, CA, USA), anti-rabbit goat antibody labeled with Alexa Fluor 594 (Thermo Fisher Scientific), anti-mouse, anti-rat, or anti-rabbit goat antibody conjugated with horseradish peroxidase (HRP) (SeraCare Life Sciences, Milford, MA, USA). The anti-CHERP rabbit polyclonal antibody was a gift from Dr. Masatoshi Maki. The anti-U2AF2 mouse monoclonal and rabbit polyclonal antibodies recognizing NXF1 were gifts from Dr. Robin Reed. The anti-UAP56 rat polyclonal antibody has been described previously [48].

### 4.4. Construction of Plasmid DNAs

To generate inducible stable cell lines, wild-type human CHERP cDNA was inserted into pcDNA5 FLAG FRT/TO. Cloning sites of individual plasmid DNA are described in Table S2. The template DNA containing full-length CHERP cDNA was obtained from Dr. Masatoshi Maki. Wild-type human CHERP was generated by PCR. Sequences were confirmed by sequencing.

For minigene splicing assays, the target region was amplified from human genomic DNA by PCR using KOD Fx Neo according to the manufacturer’s instructions, and cloned into pcDNA5 3 × FLAG FRT/TO. Mutant minigenes were obtained by mutation PCR. Sequences were confirmed by sequencing.

### 4.5. Cell Culture

U2OS cells, Hela cells, HEK293 Flp-In^TM^ T-REx^TM^ cells (Invitrogen) and U2OS stably expressing H2B-GFP were maintained in DMEM, supplemented with 10% FBS 37 °C under 5% CO_2_.

### 4.6. Establishment of Stable Cell Lines

To establish stable cell lines expressing FLAG-CHERP wild-type, CHERP expression vector, pcDNA5 FLAG FRT/TO, was transfected with Lipofectamine 2000 according to the manufacturer’s instructions. Successfully transfected cells were selected using 100 ng/mL hygromycin in DMEM containing 10% FBS. A colony was picked up and expanded in a 6-well plate. To induce the expression of FLAG-CHERP, 1 μg/mL of doxycycline was added to the culture medium for at least 24 h. Cell lysates were separated by sodium dodecyl sulfate-polyacrylamide gel electrophoresis (SDS-PAGE) and analyzed by Western blot. U2OS cells expressing H2B-GFP were described previously [48,49]. 

### 4.7. Immunostaining

Cells (1 × 10^5^ cells/mL) were seeded and cultured on coverslips. After 24 h, cells were fixed with 4% formaldehyde in cultured medium. Then, cells were washed once with phosphate-buffered saline (PBS) and blocked with PBS containing 6% bovine serum albumin for 1 h. The coverslips were incubated with anti-CHERP antiserum in PBS containing 2% bovine serum albumin, washed with PBS three times, and incubated with goat anti-rabbit antibody labeled with Alexa Fluor 594. DNA was counterstained with DAPI.

### 4.8. RNA-FISH

RNA-FISH was performed as described previously [49,50]. Briefly, cells (1 × 10^5^ cells/mL) were seeded and cultured on coverslips. After 24 h, cells were transfected with 20 nM siRNAs using Lipofectamine 2000, according to the manufacturer’s instructions. After 48 h, cells were fixed with 10% formaldehyde and permeabilized with PBS containing 0.1% *t*-octylphenoxypolyethoxyethanol/Triton^TM^ X-100. The buffer was replaced with 2 × saline-sodium citrate buffer (SSC). Then, cells were prehybridized with ULTRAhyb^TM^-Oligo Hybridization Buffer and incubated with 20 pmol Alexa Fluor 594-labeled oligo-d(T)_45_ probe overnight. Cells were washed successively with 2 × SSC, 0.5 × SSC and 0.1 × SSC. Images were taken at random using a Zeiss Axioplan 2 (Carl Zeiss, Jena, Germany) with an OLYMPUS DP70 camera (OLYMPUS, Tokyo, Japan). Segmentation of the nuclei and the cytoplasm and quantification of poly(A)^+^ RNA signals were performed using the CellProfiler cell image analysis software (version 3.1.5) [51].

### 4.9. Preparation of RNA from Cytoplasmic, Nuclear and Whole-Cell Fractions

For the preparation of cytoplasmic and nuclear fractions, cells were scraped, washed with PBS, and treated with a cell lysis buffer containing 20 mM tris(hydroxymethyl)aminomethane (Tris)-HCl (pH 8.0), 200 mM NaCl, 1 mM MgCl_2,_ and 1% octylphenoxy poly(ethyleneoxy)ethanol/Nonidet^TM^ P-40 (NP-40) on ice for 5 min. After centrifugation at 10,000× *g* for 1 min, the supernatant represented the cytoplasmic fractions. The pellets represented the nuclear fractions. Whole-cell fractions were prepared by lysing the cells as described previously [20,49].

### 4.10. Protein Preparation from Cytoplasmic and Nuclear Extracts 

Proteins were extracted from the cytoplasmic fraction and nuclear extraction as follows. Cells were centrifuged and the pellet was suspended very carefully in three times its volume of lysis solution containing 10 mM 4-(2-hydroxyethyl) piperazine-1-ethanesulfonic acid (HEPES)-KOH (pH 7.9), 10 mM KCl, 1.5 mM MgCl_2_, 0.2 mM phenylmethylsulfonyl fluoride (PMSF), and 0.5 mM dithiothreitol (DTT). The cell solutions were then incubated on ice for 10 min, vortexed for 5 s, and centrifuged at 10,000× *g* for 10 s. The supernatants were collected as cytoplasmic fractions. The pellets were resuspended in an equal volume of solution containing 20 mM HEPES-KOH (pH 7.9), 25% glycerol, 420 mM NaCl, 1.5 mM MgCl_2_, 0.2 mM EDTA, 0.2 mM PMSF and 0.5 mM DTT. The nuclei were lysed for 20 min on ice and centrifuged at 10,000× *g* for 10 min. The resulting supernatants were the nuclear fractions. The protein content in each fraction was determined by the Bradford assay (Nacalai tesque). Protein samples were mixed with a 4 × SDS buffer (190 mM Tris-HCl [pH 6.8], 40% glycerol, 0.8% SDS, 0.2% bromophenol blue and 40 mM DTT) and boiled for 2 min.

### 4.11. Immunoprecipitations

Protein nuclear extracts were prepared from HEK293 Flp-In ^TM^ T-REx ^TM^ cells stably expressing FLAG-CHERP and immunoprecipitation was performed as described previously [30]. Briefly, samples containing nuclear extract and RNase A, for the removal of RNA, were incubated for 20 min at 30 °C and then centrifuged to remove precipitants. The supernatants were mixed with 10 μL anti-FLAG M2 antibody beads. Beads were rotated overnight at 4 °C and washed with PBS containing 0.1% Triton^TM^ X-100, 0.2 mM PMSF and 0.5 mM DTT. The proteins on the beads were recovered in SDS buffer containing 250 mM Tris-HCl, 1% SDS, 0.002% bromophenol blue and 40% glycerol. After boiling for 2 min, the eluates were transferred to a new tube and separated by SDS-PAGE.

### 4.12. Silver Staining

The protein samples were mixed with 4× SDS buffer and boiled for 2 min. Protein separation was performed using SuperSep™ Ace, 5–20%, 17-well. Silver staining of SDS-PAGE gels was carried out with Silver Staining Kit Protein, in accordance with the manufacturer’s protocol. Pictures were captured using the image analyzer LAS-1000 (FUJIFILM, Tokyo, Japan).

### 4.13. LC-MS/MS Analyses

LC-MS/MS analyses were described previously [50]. Results were analyzed using the number of peptides identified prot_matches and prot_score, which was calculated with the Mascot software (Matrix Science, London, UK). Protein scores were calculated by subtracting the prot_score of the controls from that of the CHERP-expressing cells. The identified CHERP-interacting proteins are listed in Table S1.

### 4.14. Western Blots

Samples were separated by SDS-PAGE and transferred to a PVDF membrane. Membranes were blocked with 5% skim milk and then incubated with a primary antibody overnight at 4 °C. After washing, membranes were incubated with a secondary antibody conjugated with horseradish peroxidase for 2 h at room temperature. After careful washing, the signals were detected using ECL substrate with LAS 4000 mini (GE Healthcare).

### 4.15. RNA Isolation, cDNA Synthesis and PCR

Total or cytoplasmic RNAs were isolated from whole cells or the cytoplasmic fraction, respectively, with Sepasol^®^ Ⅰ Super G, and then treated with RQ1 DNase-Free RNase to remove genomic DNA, according to the manufacturer’s instructions. Then, cDNAs were synthesized by reverse transcription using Rever Tra Ace^®^, according to the manufacturer’s instructions. PCRs were performed using KOD FX NEO and analyzed by electrophoresis using either 1% agarose gel with a Tris-acetate-EDTA (TAE) buffer, or 8% polyacrylamide gel with a Tris-borate-EDTA (TBE) buffer. PCR products were visualized by a FAS-IV gel imaging system (Nippon Genetics, Tokyo, Japan). Amplified PCR products were confirmed by sequencing.

### 4.16. Preparation of RNA-seq Library

U2OS cells (30% confluent) were transfected with 20 nM EGFP or CHERP#2 siRNA for 48 h. Whole and cytoplasmic RNAs were prepared as described above. The RNA-seq library was prepared using Truseq Stranded mRNA Library Prep Kit (Illumina, San Diego, CA, USA).

### 4.17. Bioinformatic Analyses

RNA-seq analyses of two biological replicates for each group were performed using the NextSeq High Output sequencer (Illumina). Sequence reads were mapped to the hg19 human genome using STAR (version 2.5.2b) [52]. Mapped reads were transformed to expression counts for each transcript with RSEM (version 1.2.25) [53] and a gtf file of GENCODE (version 19). Normalized counts of gene expression for each replicate were obtained by processing EBSeq (version 1.2.25) software [54]. Alternative splicing events were analyzed by rMATS [55]. Significant splicing events were captured for FDR < 0.05. Mapping results were indexed using SAMtools (version 1.9) [56] and visualized using the Integrative Genomics Viewer (IGV: version 2.3) (http://software.broadinstitute.org/software/igv/home, accessed on 25 January 2022). Gene ontology (GO) analysis was performed using Database for Annotation, Visualization and Integrated Discovery (DAVID: version 6.7) [57,58]. The splice site score was calculated using MaxEntScan [59], and branch point and polypyrimidine tract score were determined using the SVM-BPfinder [60] program. To analyze the sequence usage, 15- and 20-nucleotide sequences of exon and intron boundaries at 5′ and 3′ splice sites, respectively, were obtained by BEDtools (version 2.24.0) [61]. Motifs were visualized using Weblogo (version 2.8.2) [62]. Sequence reads were deposited in NCBI under accession number GSE193399.

### 4.18. Quantitative PCR (qPCR)

qPCRs were performed with the Thermal Cycler Dice Real Time System (Takara BIO) using Thunderbird SYBR qPCR Mix. Each sample was prepared in triplicate. The mRNA amounts were calculated by threshold cycle (Ct) values.

### 4.19. Minigene Splicing Reporter Assay

Cells were cotransfected with siRNAs and minigene reporter plasmid DNAs. Briefly, cells were seeded at 15–20% confluency per well in 6-well plates. The next day, cells were transfected with siRNAs using Lipofectamine 2000. After 48 h of incubation, cells were transfected with minigene reporter plasmid DNAs (750 ng plasmid DNAs per well) using Lipofectamine 2000. After one day of incubation, cells were collected for the preparation of total RNA, as described above. Expression of the minigene reporter was analyzed using RT-PCR, followed by electrophoresis.

### 4.20. Live Cell Imaging 

Cells expressing H2B-GFP were seeded on 35 mm-diameter glass bottom dishes (AGC techno glass, Shizuoka, Japan) and transfected with siRNAs using Lipofectamine 2000, as reported previously [20,48]. After siRNA transfection, cells were cultured for 36 h. Images were taken every 5 min using Biostation IMq (Nikon, Tokyo, Japan) at 37 °C in a humidified chamber with 5% CO_2_ for 16 h.

### 4.21. MTT Assays

Cell viability was assayed by colorimetric MTT assay [50,63]. U2OS cells were seeded at 7.5 × 10^3^ cells/mL in a 96-well plate and cultured for 24 h. siRNAs were introduced to each well and incubated for 48 h. Afterward, 5 μL of 5 mg/mL MTT was added to each well and incubated for 4 h. The cell culture plate was then centrifuged at 400× *g* for 5 min. The supernatant was removed and cells were solubilized with 10 mM NH_4_Cl containing 10% SDS (pH 7.0). Cell viability was estimated by measuring the optical density at 600 nm.

## Supplementary Materials

The following supporting information can be downloaded at: https://www.mdpi.com/article/10.3390/ijms23052555/s1.

## References

[1] Chen M., Manley J.L. (2009). Mechanisms of alternative splicing regulation: Insights from molecular and genomics approaches. Nat. Rev. Mol. Cell Biol..

[2] Fu X.D., Ares M. (2014). Context-dependent control of alternative splicing by RNA-binding proteins. Nat. Rev. Genet..

[3] Matera A.G., Wang Z. (2014). A day in the life of the spliceosome. Nat. Rev. Mol. Cell Biol..

[4] Wahl M.C., Will C.L., Lührmann R. (2009). The Spliceosome: Design Principles of a Dynamic RNP Machine. Cell.

[5] Black D.L. (2003). Mechanisms of alternative pre-messenger RNA splicing. Annu. Rev. Biochem..

[6] Wilkinson M.E., Charenton C., Nagai K. (2020). RNA Splicing by the Spliceosome. Annu. Rev. Biochem..

[7] Moore M.J., Query C.C., Sharp P.A. (1999). Splicing of Precursors to mRNA by the Spliceosome.

[8] Kim E., Goren A., Ast G. (2008). Alternative splicing: Current perspectives. BioEssays.

[9] Keren H., Lev-Maor G., Ast G. (2010). Alternative splicing and evolution: Diversification, exon definition and function. Nat. Rev. Genet..

[10] Zhou Z., Fu X.D. (2013). Regulation of splicing by SR proteins and SR protein-specific kinases. Chromosoma.

[11] Geuens T., Bouhy D., Timmerman V. (2016). The hnRNP family: Insights into their role in health and disease. Hum. Genet..

[12] Maris C., Dominguez C., Allain F.H.T. (2005). The RNA recognition motif, a plastic RNA-binding platform to regulate post-transcriptional gene expression. FEBS J..

[13] Laplante J.M., O’Rourke F., Lu X., Fein A., Olsen A., Feinstein M.B. (2000). Cloning of human Ca2+ homoeostasis endoplasmic reticulum protein (CHERP): Regulated expression of antisense cDNA depletes CHERP, inhibits intracellular Ca^2+^ mobilization and decreases cell proliferation. Biochem. J..

[14] Ryan T., Sharma P., Ignatchenko A., MacLennan D.H., Kislinger T., Gramolini A.O. (2011). Identification of novel ryanodine receptor 1 (RyR1) protein interaction with calcium homeostasis endoplasmic reticulum protein (CHERP). J. Biol. Chem..

[15] Lin-Moshier Y., Sebastian P.J., Higgins L.A., Sampson N.D., Hewitt J.E., Marchant J.S. (2013). Re-evaluation of the role of Calcium Homeostasis Endoplasmic Reticulum Protein (CHERP) in cellular calcium signaling. J. Biol. Chem..

[16] Sasaki-Osugi K., Imoto C., Takahara T., Shibata H., Maki M. (2013). Nuclear ALG-2 protein interacts with Ca^2+^ homeostasis endoplasmic reticulum protein (CHERP) Ca^2+^-dependently and participates in regulation of alternative splicing of inositol trisphosphate receptor type 1 (IP3R1) Pre-mRNA. J. Biol. Chem..

[17] De Maio A., Yalamanchili H.K., Adamski C.J., Gennarino V.A., Liu Z., Qin J., Jung S.Y., Richman R., Orr H., Zoghbi H.Y. (2018). RBM17 Interacts with U2SURP and CHERP to Regulate Expression and Splicing of RNA-Processing Proteins. Cell Rep..

[18] Yoshimoto R., Kaida D., Furuno M., Burroughs A.M., Noma S., Suzuki H., Kawamura Y., Hayashizaki Y., Mayeda A., Yoshida M. (2017). Global analysis of pre-mRNA subcellular localization following splicing inhibition by spliceostatin A. RNA.

[19] Legrain P., Rosbash M. (1989). Some cis- and trans-acting mutants for splicing target pre-mRNA to the cytoplasm. Cell.

[20] Okamura M., Yamanaka Y., Shigemoto M., Kitadani Y., Kobayashi Y., Kambe T., Nagao M., Kobayashi I., Okumura K., Masuda S. (2018). Depletion of mRNA export regulator DBP5/ DDX19, GLE1 or IPPK that is a key enzyme for the production of IP6, resulting in differentially altered cytoplasmic mRNA expression and specific cell defect. PLoS ONE.

[21] Zamore P.D., Green M.R. (1989). Identification, purification, and biochemical characterization of U2 small nuclear ribonucleoprotein auxiliary factor. Proc. Natl. Acad. Sci. USA.

[22] Hegele A., Kamburov A., Grossmann A., Sourlis C., Wowro S., Weimann M., Will C.L., Pena V., Lührmann R., Stelzl U. (2012). Dynamic Protein-Protein Interaction Wiring of the Human Spliceosome. Mol. Cell.

[23] Ahn E.Y., DeKelver R.C., Lo M.C., Nguyen T.A., Matsuura S., Boyapati A., Pandit S., Fu X.D., Zhang D.E. (2011). SON Controls Cell-Cycle Progression by Coordinated Regulation of RNA Splicing. Mol. Cell.

[24] Martín E., Vivori C., Rogalska M., Herrero-Vicente J., Valcárcel J. (2021). Alternative splicing regulation of cell-cycle genes by SPF45/SR140/CHERP complex controls cell proliferation. RNA.

[25] Lu X., Göke J., Sachs F., Jacques P.É., Liang H., Feng B., Bourque G., Bubulya P.A., Ng H.H. (2013). SON connects the splicing-regulatory network with pluripotency in human embryonic stem cells. Nat. Cell Biol..

[26] Damianov A., Ying Y., Lin C.H., Lee J.A., Tran D., Vashisht A.A., Bahrami-Samani E., Xing Y., Martin K.C., Wohlschlegel J.A. (2016). Rbfox Proteins Regulate Splicing as Part of a Large Multiprotein Complex LASR. Cell.

[27] Ying Y., Wang X.J., Vuong C.K., Lin C.H., Damianov A., Black D.L. (2017). Splicing Activation by Rbfox Requires Self-Aggregation through Its Tyrosine-Rich Domain. Cell.

[28] Cheah P.Y., Wong Y.H., Koh P.K., Loi C., Chew M.H., Tang C.L. (2014). A novel indel in exon 9 of APC upregulates a “skip exon 9” isoform and causes very severe familial adenomatous polyposis. Eur. J. Hum. Genet..

[29] Cho S., Moon H., Loh T.J., Jang H.N., Liu Y., Zhou J., Ohn T., Zheng X., Shen H. (2015). Splicing inhibition of U2AF^65^ leads to alternative exon skipping. Proc. Natl. Acad. Sci. USA.

[30] Echeverria G.V., Cooper T.A. (2014). Muscleblind-like 1 activates insulin receptor exon 11 inclusion by enhancing U2AF65 binding and splicing of the upstream intron. Nucleic Acids Res..

[31] Anczuków O., Akerman M., Cléry A., Wu J., Shen C., Shirole N.H., Raimer A., Sun S., Jensen M.A., Hua Y. (2015). SRSF1-Regulated Alternative Splicing in Breast Cancer. Mol. Cell.

[32] Anczuków O., Rosenberg A.Z., Akerman M., Das S., Zhan L., Karni R., Muthuswamy S.K., Krainer A.R. (2012). The splicing factor SRSF1 regulates apoptosis and proliferation to promote mammary epithelial cell transformation. Nat. Struct. Mol. Biol..

[33] Calarco J.A., Superina S., O’Hanlon D., Gabut M., Raj B., Pan Q., Skalska U., Clarke L., Gelinas D., van der Kooy D. (2009). Regulation of Vertebrate Nervous System Alternative Splicing and Development by an SR-Related Protein. Cell.

[34] Dauksaite V., Gotthardt M. (2018). Molecular basis of titin exon exclusion by RBM20 and the novel titin splice regulator PTB4. Nucleic Acids Res..

[35] Ray D., Kazan H., Chan E.T., Castillo L.P., Chaudhry S., Talukder S., Blencowe B.J., Morris Q., Hughes T.R. (2009). Rapid and systematic analysis of the RNA recognition specificities of RNA-binding proteins. Nat. Biotechnol..

[36] Ajiro M., Jia R., Yang Y., Zhu J., Zheng Z.M. (2015). A genome landscape of SRSF3-regulated splicing events and gene expression in human osteosarcoma U2OS cells. Nucleic Acids Res..

[37] Zhou X., Wu W., Li H., Cheng Y., Wei N., Zong J., Feng X., Xie Z., Chen D., Manley J.L. (2014). Transcriptome analysis of alternative splicing events regulated by SRSF10 reveals position-dependent splicing modulation. Nucleic Acids Res..

[38] Kuroyanagi H., Watanabe Y., Suzuki Y. (2013). Position-dependent and neuron-specific splicing regulation by the CELF family RNA-binding protein UNC-75 in Caenorhabditis elegans. Nucleic Acids Res..

[39] Bechara E.G., Sebestyén E., Bernardis I., Eyras E., Valcárcel J. (2013). RBM5, 6, and 10 differentially regulate NUMB alternative splicing to control cancer cell proliferation. Mol. Cell.

[40] Imai T., Tokunaga A., Yoshida T., Hashimoto M., Mikoshiba K., Weinmaster G., Nakafuku M., Okano H. (2001). The Neural RNA-Binding Protein Musashi1 Translationally Regulates Mammalian numb Gene Expression by Interacting with Its mRNA. Mol. Cell. Biol..

[41] Ruth Zearfoss N., Deveau L.M., Clingman C.C., Schmidt E., Johnson E.S., Massi F., Ryder S.P. (2014). A conserved three-nucleotide core motif defines musashi RNA binding specificity. J. Biol. Chem..

[42] Chawla G., Lin C.-H., Han A., Shiue L., Ares M., Black D.L. (2009). Sam68 Regulates a Set of Alternatively Spliced Exons during Neurogenesis. Mol. Cell. Biol..

[43] Iijima T., Wu K., Witte H., Hanno-Iijima Y., Glatter T., Richard S., Scheiffele P. (2011). SAM68 regulates neuronal activity-dependent alternative splicing of neurexin-1. Cell.

[44] Wang Q., Wang Y., Liu Y., Zhang C., Luo Y., Guo R., Zhan Z., Wei N., Xie Z., Shen L. (2019). U2-related proteins CHERP and SR140 contribute to colorectal tumorigenesis via alternative splicing regulation. Int. J. Cancer.

[45] Moore M.J., Wang Q., Kennedy C.J., Silver P.A. (2010). An alternative splicing network links cell-cycle control to apoptosis. Cell.

[46] Ramírez-valle F., Badura M.L., Braunstein S., Narasimhan M., Schneider R.J. (2010). Mitotic Raptor Promotes mTORC1 Activity, G 2/M Cell Cycle Progression, and Internal Ribosome Entry Site-Mediated. Mol. Cell. Biol..

[47] Maggioni D., Garavello W., Rigolio R., Pignataro L., Gaini R., Nicolini G. (2013). Apigenin impairs oral squamous cell carcinoma growth in vitro inducing cell cycle arrest and apoptosis. Int. J. Oncol..

[48] Yamazaki T., Fujiwara N., Yukinaga H., Ebisuya M., Shiki T., Kurihara T., Kioka N., Kambe T., Nagao M., Nishida E. (2010). The Closely Related RNA helicases, UAP56 and URH49, Preferentially Form Distinct mRNA Export Machineries and Coordinately Regulate Mitotic Progression. Mol. Biol. Cell.

[49] Fujita K., Yamazaki T., Harada K., Seno S., Matsuda H., Masuda S. (2020). URH49 exports mRNA by remodeling complex formation and mediating the NXF1-dependent pathway. Biochim. Biophys. Acta Gene Regul. Mech..

[50] Kurata M., Fujiwara N., Fujita K., Yamanaka Y., Seno S., Kobayashi H., Miyamae Y., Takahashi N., Shibuya Y., Masuda S. (2019). Food-Derived Compounds Apigenin and Luteolin Modulate mRNA Splicing of Introns with Weak Splice Sites. iScience.

[51] Carpenter A.E., Jones T.R., Lamprecht M.R., Clarke C., Kang I.H., Friman O., Guertin D.A., Chang J.H., Lindquist R.A., Moffat J. (2006). CellProfiler: Image analysis software for identifying and quantifying cell phenotypes. Genome Biol..

[52] Dobin A., Davis C.A., Schlesinger F., Drenkow J., Zaleski C., Jha S., Batut P., Chaisson M., Gingeras T.R. (2013). STAR: Ultrafast universal RNA-seq aligner. Bioinformatics.

[53] Bo L., Dewey C.N. (2011). RSEM: Accurate transcript quantification from RNA-Seq data with or without a reference genome. BMC Bioinform..

[54] Leng N., Dawson J.A., Thomson J.A., Ruotti V., Rissman A.I., Smits B.M.G., Haag J.D., Gould M.N., Stewart R.M., Kendziorski C. (2013). EBSeq: An empirical Bayes hierarchical model for inference in RNA-seq experiments. Bioinformatics.

[55] Shen S., Park J.W., Lu Z., Lin L., Henry M.D., Wu Y.N., Zhou Q., Xing Y. (2014). rMATS: Robust and flexible detection of differential alternative splicing from replicate RNA-Seq data. Proc. Natl. Acad. Sci. USA.

[56] Danecek P., Bonfield J.K., Liddle J., Marshall J., Ohan V., Pollard M.O., Whitwham A., Keane T., McCarthy S.A., Davies R.M. (2021). Twelve years of SAMtools and BCFtools. Gigascience.

[57] Huang D.W., Sherman B.T., Lempicki R.A. (2009). Systematic and integrative analysis of large gene lists using DAVID bioinformatics resources. Nat. Protoc..

[58] Huang D.W., Sherman B.T., Lempicki R.A. (2009). Bioinformatics enrichment tools: Paths toward the comprehensive functional analysis of large gene lists. Nucleic Acids Res..

[59] Yeo G., Burge C.B. (2004). Maximum entropy modeling of short sequence motifs with applications to RNA splicing signals. J. Comput. Biol..

[60] Corvelo A., Hallegger M., Smith C.W.J., Eyras E. (2010). Genome-Wide Association between Branch Point Properties and Alternative Splicing. PLoS Comput. Biol..

[61] Quinlan A.R., Hall I.M. (2010). BEDTools: A flexible suite of utilities for comparing genomic features. Bioinformatics.

[62] Crooks G., Hon G., Chandonia J., Brenner S. (2004). WebLogo: A sequence logo generator. Genome Res..

[63] Li J., Huang H., Sun L., Yang M., Pan C., Chen W., Wu D., Lin Z., Zeng C., Yao Y. (2009). MiR-21 indicates poor prognosis in tongue squamous cell carcinomas as an apoptosis inhibitor. Clin. Cancer Res..

