# Supplementary material for: CHERP Regulates the Alternative Splicing of pre-mRNAs in the Nucleus
Source: Int J Mol Sci. 2022 Feb 25;23(5):2555. doi: 10.3390/ijms23052555 (PMC8910253; doi:10.3390/ijms23052555)
Supplement: Supplementary file 1 [file ijms-23-02555-s001.zip › Supplementary Figure Legend.pdf]

Supplementary Figure Legend

Figure S1. Role of CHERP in mRNA metabolism in the nucleus.

(**A**) Expression of CHERP in U2OS cells after transfection with siRNAs against EGFP (control) or CHERP (CHERP#1) determined by Western blot. GAPDH was used to control sample loading. Samples were prepared 48 h after transfection. (**B**) Localization of poly(A)$^+$ RNAs analyzed by RNA-FISH 48 h after transfection of U2OS cells with siRNA against EGFP (control) or CHERP (CHERP#1). Poly(A)$^+$ RNAs were detected with Alexa 594 labeled oligo-d(T)$_{45}$ probes. DNA was stained with DAPI. Scale bar, 20 μm. (**C**) CHERP interacting partners were purified from nuclear extracts from HEK293 Flp-In ^{TM} T-REx ^{TM} cells expressing FLAG-CHERP and were analyzed by silver staining. (**D**) Venn diagram showing the number of proteins interacting with CHERP. The numbers indicate, from left to right, the number of proteins interacting with CHERP in only the first analysis, in both analysis the first and the second analysis, and in only the second analysis. (**E**) GO groups enriched in proteins interacting with FLAG-CHERP. Proteins with protein score > 0 (Tables S1) were analyzed by GO term. (**F**) CHERP interacting proteins were analyzed by Western blot. The antibodies are indicated on the left side of each panel. (**G**) CHERP mRNA expression in U2OS cells transfected with siRNAs against EGFP (control) or CHERP (CHERP#2) determined by RT-qPCR. The expression levels were normalized to PGK1 levels. (**H**) The efficacy of the subcellular fractionation for RNA preparation was confirmed by RT-PCR using tRNA (mostly cytoplasmic fraction), U6 small nuclear RNA (snRNA) snRNA (nuclear fraction), and E2F transcription factor 8 (E2F8) (E2F8) (unspliced transcript: nuclear fraction, spliced transcript: both fractions).

Figure S2. CHERP regulates retained introns in transcripts.

(**A**) Quantile log plots showing expression and fold changes of III (left) and DII (right) with FDR < 0.05 in the cytoplasm. Red and blue dots indicate the levels of III and DII, respectively, of corresponding mRNAs. (**B**) GO groups enriched in transcripts with retained introns (RI) after CHERP depletion. FDR < 0.05, inclusion or skipping count ≧ 10 and IncLevelDifference ≧ |0.1| for RNA-Seq data was considered statistically significant.

Figure S3. Characteristics of retained introns induced by CHERP depletion.

(**A, B**) 5' and 3' splice site scores after CHERP depletion calculated by MaxEntScan. (**C**) Branch point site scores after CHERP depletion calculated by SVM-BPfinder. (**D**) Polypyrimidine tract site scores after CHERP depletion calculated by SVM-BPfinder. (**E, F**) Length and GC content of CHERP-targeted introns analyzed by BedTools. (**A–F**) FDR < 0.05, inclusion or skipping count ≧ 10, and IncLevelDifference ≧ |0.1| were considered significant. All introns were used as references introns. For statistical analyses, Wilcoxon rank sum test was used to calculate P-value. (**G**) Nucleotide sequences of exon-intron boundaries in retained introns and references estimated by BedTools.

Figure S4. CHERP regulates skipped exons in transcripts.

(**A**) Quantile-log plot expression level and fold changes of EI (left) or ES (right) with FDR < 0.05 in the cytoplasm. Orange and green dots indicate the levels of EI and ES, respectively, for corresponding mRNAs in the cytoplasm. (**B**) GO groups enriched in transcripts with skipped exons after CHERP depletion. FDR < 0.05, inclusion or skipping count ≧ 10 and IncLevelDifference ≧ |0.1| for RNA-Seq data was considered statistically significant.

Figure S5. Characteristics of SE induced by CHERP depletion.
(**A, B**) 5’ and 3’ splice site scores in the CHERP-regulated splice sites calculated by MaxEntScan. (**C**) Branch point site scores after CHERP depletion calculated by SVM-BPfinder. (**D**) Polypyrimidine tract site scores after CHERP depletion calculated by SVM-BPfinder. (**E, F**) Length and GC content of CHERP-targeted introns analyzed by BedTools. (**A–F**) FDR < 0.05, inclusion or skipping count ≧ 10, and IncLevelDifference ≧ |0.1| was considered statistically significant. All introns were used as references introns. For statistical analyses, Wilcoxon rank sum test was used to calculate P-value. (**G**) Nucleotide sequences of exon-intron boundaries in SE and references estimated by BedTools.

Figure S6. GO analysis of mRNA with increased and decreased cytoplasmic expression after CHERP depletion.
(**A**) GO groups enriched in mRNAs with a significantly increased cytoplasmic expression after CHERP depletion. FDR < 0.05 and fold change ≧ 1.5 for RNA-Seq data was considered statistically significant. (**B**) GO groups enriched in mRNAs with a significantly decreased cytoplasmic expression after CHERP depletion. FDR < 0.05 and fold change ≦ 0.67 for RNA-Seq data was considered statistically significant.
