# Supplementary material for: CHERP Regulates the Alternative Splicing of pre-mRNAs in the Nucleus
Source: Int J Mol Sci. 2022 Feb 25;23(5):2555. doi: 10.3390/ijms23052555 (PMC8910253; doi:10.3390/ijms23052555)

GeneName	prot_desc	prot_acc	prot_mass	prot_.score	prot_.matches
1	Uncharacterize	K7EIP6	2801	0	0
2	Uncharacterize	H7BZT4	10832	124	2
3	Uncharacterize	H3BRM9	9615	0	0
4	Uncharacterize	H0YHG0	59149	34	0
5	Ig kappa chain	P01614	12668	0	1
6	Ig kappa chain	P01616	12048	0	0
7	Uncharacterize	F5H423	23331	133	2
8	Uncharacterize	H7C0C1	21328	39	1
A2M	Alpha-2-macro	P01023	163188	0	0
AAAS	Aladin (Fragme	H3BU82	38494	0	0
AARD	Alanine and ar	Q4LEZ3	17564	0	0
AATF	Protein AATF (	K7EK88	11404	0	0
ABAT	4-aminobutyra	H3BNQ7	57886	0	0
ABCA2	ATP-binding ca	H0Y8C9	118053	0	0
ABCB6	ATP-binding ca	H7BXK9	77498	0	0
ABCD3	ATP-binding ca	F5GYC1	67273	0	0
ABT1	Activator of ba	Q9ULW3	31060	0	0
ACAD11	ACAD11 protei	Q08AE9	33755	0	0
ACAT1	Acetyl-CoA ace	G3XAB4	45171	220	4
ACIN1	Apoptotic chro	S4R3H4	145355	46	1
ACTA1	Actin, alpha sk	Q5T8M7	37800	0	0
ACTB	Actin, cytoplas	P60709	41710	0	0
ACTBL2	Beta-actin-like	Q562R1	41976	0	0
ACTC1	Actin, alpha ca	P68032	41992	0	0
ACTG1	Actin, cytoplas	I3L1U9	23830	0	0
ACTL6A	Actin-like prote	O96019	47430	17	0
ACTN1	Alpha-actinin-1	G3V2W4	27083	0	0
ACTN4	Alpha-actinin-4	O43707	104788	133	3
ACTR1A	Alpha-centract	B4DXP9	37457	0	0
ACTR2	Actin-related p	F5H6T1	34501	0	0
ADAD2	Adenosine dea	H3BNP6	28653	0	0
ADAR	Double-strande	E7ENU4	140739	486	12
ADARB1	Adenosine dea	G5E9B4	79957	0	0
ADARB2	Double-strande	Q9NS39	80571	0	0
ADD1	Adducin 1 (Alp	A2A3N8	73287	0	0
ADD3	Gamma-adduc	Q9UEY8	79105	0	0
AGO1	Protein argona	Q5TA58	88822	77	2
AGO2	Protein argona	Q9UKV8	97146	0	0

AHCTF1	Protein ELYS (	Q8WYP5	252342	0	0
AHNAK	Neuroblast diff	Q09666	628699	79	2
AIFM1	Apoptosis-indu	O95831	66859	0	0
AK2	Adenylate kina	F8VY04	25614	201	5
AKAP8	A-kinase ancho	O43823	76061	175	4
AKAP8L	A-kinase ancho	Q9ULX6	71604	0	0
AKR1B15	Aldo-keto redu	C9JRZ8	39435	0	0
ALDOA	Fructose-bisph	H3BQN4	39793	343	7
ALDOC	Fructose-bisph	P09972	39431	172	3
ALG13	Isoform 2 of Pu	Q9NP73-2	18213	0	0
ALYREF	THO complex s	E9PB61	27541	0	0
ANKEF1	Ankyrin repeat	Q9NU02	86610	0	0
ANKFY1	Ankyrin repeat	Q9P2R3	128318	55	1
ANP32A	Acidic leucine-	H0YN26	19985	219	5
ANP32B	Acidic leucine-	Q92688	28770	205	5
ANXA1	Annexin A1 OS	P04083	38690	0	0
ANXA2	Annexin (Fragr	H0YMD0	25326	60	1
ANXA6	Annexin OS=H	E5RK69	51744	47	1
AP2A1	AP-2 complex :	O95782	107478	0	0
AP2A2	AP-2 complex :	O94973	103895	0	0
AP2B1	AP-2 complex :	K7EJT8	101268	0	0
AP2M1	AP-2 complex :	C9JJ47	28773	0	0
AP2S1	AP-2 complex :	M0QYZ2	18917	0	0
APEX1	DNA-(apurinic	G3V359	19156	73	1
APH1B	Gamma-secret	H0YKZ9	20749	0	0
API5	Apoptosis inhil	H0YER7	57525	69	1
APRT	Adenine phosp	H3BQB1	17551	22	0
AQR	Intron-binding	O60306	171186	47	1
ARF3	ADP-ribosylati	B7ZB63	16135	0	0
ARF4	ADP-ribosylati	P18085	20498	148	2
ARF6	ADP-ribosylati	P62330	20069	38	1
ARG1	Arginase-1 OS	P05089	34713	0	0
ARGLU1	Arginine and gl	Q9NWB6	33197	0	0
ARHGEF18	Rho guanine nu	M0QZS0	91352	0	0
ARID1B	AT-rich interac	H0Y2R3	76153	0	0
ARID2	AT-rich interac	F8WCU9	175042	0	0
ARL1	ADP-ribosylati	B4DWW1	18554	0	0
ARMC5	Armadillo repea	J3KQ26	108000	0	0
ARPC2	Actin related p	G5E9J0	10153	0	0

ARPC4-TTLL3	Protein ARPC4	F8WCF6	21045	0	0
ASAP2	Arf-GAP with S	O43150	111581	0	0
ATAD1	ATPase family	B4E2J1	30615	0	0
ATAD3A	ATPase family	H0Y2W2	64315	0	0
ATAD3B	ATPase family	Q5T9A4	72527	0	0
ATP12A	Potassium-tran	P54707	115437	0	0
ATP13A1	Probable catio	Q9HD20	132870	35	1
ATP1A1	Sodium/potass	P05023	112824	0	0
ATP1B3	Sodium/potass	H7C547	4914	0	0
ATP2A2	Sarcoplasmic/	P16615	114683	0	0
ATP5A1	ATP synthase s	P25705	59714	149	4
ATP5B	ATP synthase s	P06576	56525	0	0
ATP5C1	ATP synthase s	B4DL14	27495	0	0
ATP5D	ATP synthase s	P30049	17479	114	2
ATP5G1	ATP synthase l	I3L0Y5	10032	34	1
ATP5J2-PTCD1	Pentatricopept	G3V325	84057	0	0
ATP5L	ATP synthase s	E9PN17	8447	40	1
ATP5O	ATP synthase s	H7C086	8128	0	0
ATP6AP2	Renin receptor	H0Y750	26616	0	0
ATP6V0A1	V-type proton /	F5H569	96251	0	0
ATP6V1C1	V-type proton /	E7EV59	35686	0	0
ATPIF1	ATPase inhibit	Q9UII2	12241	38	1
BAG2	BAG family mo	A2A296	12173	0	0
BANF1	Barrier-to-auto	O75531	10052	0	0
BARD1	BRCA1-associa	E7EUI3	62141	0	0
BAZ1A	Bromodomain a	Q9NRL2	178592	0	0
BAZ1B	Tyrosine-prote	Q9UIG0	170796	0	0
BCAP31	B-cell receptor	P51572	27974	157	3
BCAS2	Pre-mRNA-spl	O75934	26115	0	0
BCLAF1	Bcl-2-associat	E9PK09	83183	0	0
BLOC1S6	Biogenesis of l	H3BMT8	5720	40	1
BMS1	Ribosome biog	Q14692	145716	0	0
BOLA2	BolA-like prote	H3BTW0	9848	53	1
BRI3BP	BRI3-binding p	Q8WY22	27818	0	0
BRIX1	Ribosome biog	Q8TDN6	41375	0	0
BSG	Basigin OS=Ho	P35613	42174	0	0
BUB3	Mitotic checkp	J3QT28	31684	118	3
BUD31	Protein BUD31	C9JNV2	13559	0	0
BYSL	Bystin (Fragme	H7BY94	20988	0	0

C11orf84	Uncharacterize	Q9BUA3	41011	0	0
C12orf23	UPF0444 trans	Q8WUH6	11741	0	0
C14orf166	UPF0568 prote	Q9Y224	28051	450	9
C16orf80	UPF0468 prote	Q9Y6A4	22760	199	4
C19orf10	UPF0556 prote	Q969H8	18783	64	2
C19orf43	Uncharacterize	Q9BQ61	18408	197	9
C1QBP	Complement co	Q07021	31343	116	2
C21orf33	ES1 protein ho	H7C1F6	27429	54	1
C4A	Complement C	P0C0L4	192664	0	0
C4orf22	Uncharacterize	G5E9Y8	17782	0	0
C6orf47	Uncharacterize	O95873	31690	0	0
CALM2	Calmodulin (Fr	H0Y7A7	20749	50	1
CALML3	Calmodulin-like	P27482	16880	0	0
CALR	Calreticulin OS	P27797	48112	252	7
CALU	Calumenin OS=	O43852	37084	0	0
CAND1	Cullin-associat	Q86VP6	136289	38	1
CANX	Calnexin OS=H	B4DGP8	71458	0	0
CAPZA1	F-actin-cappin	P52907	32902	0	0
CAPZA2	F-actin-cappin	P47755	32929	0	0
CAPZB	Capping protei	B1AK87	29277	0	0
CASK	Peripheral plas	Q5JS72	59327	0	0
CASP14	Caspase-14 OS	P31944	27662	0	0
CBX1	Chromobox pro	J3KS05	20115	41	1
CBX3	Chromobox pro	Q13185	20798	0	0
CBX5	Chromobox pro	P45973	22211	0	0
CCAR1	Cell division cy	Q8IX12	132739	1828	46
CCDC154	Coiled-coil don	H3BS06	11274	0	0
CCDC181	Coiled-coil don	Q5TID7	60066	0	0
CCNK	Cyclin-K OS=H	G3V5E1	43377	0	0
CCT4	T-complex prot	B7Z2F4	57888	163	4
CCT5	T-complex prot	B4DYD8	49495	58	1
CCT8	T-complex prot	B4DEM7	57608	31	1
CDC40	Pre-mRNA-pro	Q5SRN1	60792	0	0
CDC5L	Cell division cy	Q99459	92194	0	0
CDIPT	CDP-diacylglyc	B3KY94	25929	0	0
CDK2	Cyclin-depende	G3V5T9	39153	0	0
CENPV	Centromere pro	Q7Z7K6	29927	0	0
CERS2	Ceramide synth	H0YKH6	26788	0	0
CFL1	Cofilin 1 (Non-	G3V1A4	16801	33	1

CFTR	Cystic fibrosis	E7EPB6	163250	35	1
CHCHD3	Coiled-coil-hel	G3V1K1	15549	0	0
CHD1	Chromodomair	O14646	196567	0	0
CHD3	Chromodomair	Q12873	226450	0	0
CHD4	Chromodomair	F5GWX5	216967	111	3
CHD7	Chromodomair	Q9P2D1	335717	0	0
CHD8	Chromodomair	H0YJG4	101664	0	0
CHERP	Calcium homeo	Q8IWX8	103637	1107	64
CHMP1A	Charged multiv	F8VUA2	19519	0	0
CHMP5	Charged multiv	Q9NZZ3	24555	0	0
CHN1	N-chimaerin O:	B8ZZ96	31322	30	1
CHTOP	Chromatin targ	Q9Y3Y2	26380	0	0
CIRBP	Cold-inducible	B4E2X2	28342	6	0
CIRH1A	Cirhin (Fragme	H3BSH7	77894	0	0
CISD2	CDGSH iron-su	I3L1N9	5141	0	0
CKAP4	Cytoskeleton-a	Q07065	65983	0	0
CKAP5	Cytoskeleton-a	Q14008	225352	123	3
CKB	Creatine kinase	P12277	42617	103	2
CKMT1A	Creatine kinase	P12532	47007	71	2
CLIC4	Chloride intrac	Q9Y696	28754	0	0
CLINT1	Clathrin interac	Q14677	68216	0	0
CLK3	CDC-like kinas	G5E959	56193	0	0
CLPB	Caseinolytic pe	H0YFF5	37195	0	0
CLTA	Clathrin light cl	P09496	27060	0	0
CLTB	Clathrin light cl	P09497	25175	0	0
CLTC	Clathrin heavy	Q00610	191493	653	13
CMAS	N-acylneurami	F5GYM0	11647	0	0
CNPY2	Protein canopy	F8VXJ7	19040	52	1
CNTNAP4	Contactin-assc	F5H107	139833	11	1
CNTROB	Centrobin OS=	Q8N137	101191	0	0
COIL	Coilin OS=Hon	P38432	62570	0	0
COL1A1	Collagen alpha	P02452	138857	0	0
COL1A2	Collagen alpha	P08123	129235	0	0
COMT	Catechol O-me	E7EMS6	24822	47	1
COPA	Coatomer subu	P53621	138258	0	0
COPB1	Coatomer subu	E9PP73	55502	0	0
COPB2	Coatomer prote	B4DZI8	98984	47	1
COPG2	Coatomer subu	Q9UBF2	97560	48	1
CORO1C	Coronin-1C OS	Q9ULV4	53215	0	0

COX17	Cytochrome c (	C9J8T6	10846	0	0
COX4I1	Cytochrome c (	H3BN72	14359	0	0
COX5A	Cytochrome c (	H3BNX8	17224	0	0
COX5B	Cytochrome c (	P10606	13687	0	0
COX6B1	Cytochrome c (	P14854	10186	0	0
COX6C	Cytochrome c (	P09669	8776	0	0
COX7A2	Cytochrome c (	H0UI06	12836	59	1
COX7C	Cytochrome c (	D6R9Z7	6378	0	0
CPA5	Carboxypeptida	Q8WXQ8	49005	30	1
CPD	Carboxypeptida	O75976	152835	0	0
CPSF1	Cleavage and p	Q10570	160782	76	2
CPSF2	Cleavage and p	Q9P2I0	88431	0	0
CPSF3	Cleavage and p	G5E9W3	73430	0	0
CPSF4	Cleavage and p	B7Z7B0	21942	0	0
CPSF6	Cleavage and p	F8WJN3	52238	223	4
CPSF7	Cleavage and p	F5H669	41241	150	3
CPVL	Probable serine	Q9H3G5	54129	88	2
CREB3	Cyclic AMP-res	O43889	43889	0	0
CRNKL1	Crooked neck-	Q5JY65	99111	0	0
CROCC	Rootletin (Frag	B1AKD8	149087	0	0
CRTAP	Cartilage-asso	C9JP16	41406	178	5
CRYAB	Alpha-crystallin	E9PJL7	15436	0	0
CS	Citrate synthas	H0YIC4	14114	73	1
CSE1L	Exportin-2 OS=	B4DUC5	85418	0	0
CSNK2A1	Casein kinase I	E7EU96	45282	0	0
CSNK2B-LY6G	Chimera CSNK	Q5SRQ3	26656	68	1
CST6	Cystatin-M OS	Q15828	16500	0	0
CSTA	Cystatin-A OS=	C9J0E4	7082	0	0
CSTB	Cystatin-B OS=	P04080	11133	0	0
CSTF1	Cleavage stimu	Q05048	48327	0	0
CSTF2	Cleavage stimu	E7EWR4	62902	0	0
CTBP2	C-terminal-bin	Q5SQP8	56066	32	1
CTNNB1	Catenin beta-1	B4DGU4	84712	0	0
CTNNBL1	Beta-catenin-li	B4DE16	61932	0	0
CTNND1	Catenin delta-1	C9JZR2	104784	0	0
CTSD	Cathepsin D lig	H7C1V0	20359	0	0
CTTNBP2	Cortactin-bindi	H0Y448	125549	2	0
CUL4B	Cullin 4B, isofo	K4DI93	102691	0	0
CYB5B	Cytochrome b5	H3BUX2	15707	0	0

CYB5R3	NADH-cytochr	B1AHF3	16686	0	0
CYCS	Cytochrome c (	C9JFR7	11326	50	1
DAD1	Dolichyl-dipho:	F5GXX5	9548	91	2
DAPP1	Dual adapter fo	J3KNB3	30156	1	0
DAZAP1	DAZ-associate	K7EQ02	24789	84	1
DBN1	Drebrin OS=Hc	Q16643	71385	0	0
DCAKD	Dephospho-Co	K7ESP4	24162	0	0
DCD	Dermcidin OS=	P81605	11277	0	0
DCXR	L-xylulose redu	J3KRZ4	14883	56	1
DDB1	DNA damage-b	F5GY55	126887	119	3
DDOST	Dolichyl-dipho:	E7EWT1	46548	0	0
DDRGK1	DDRGK domai	Q96HY6	35589	0	0
DDX1	ATP-dependen	Q92499	82380	70	1
DDX17	Probable ATP-	H3BLZ8	80389	225	6
DDX18	ATP-dependen	Q9NVP1	75359	0	0
DDX21	Nucleolar RNA	Q9NR30	87290	0	0
DDX23	Probable ATP-	Q9BUQ8	95524	0	0
DDX27	Probable ATP-	Q96GQ7	89779	0	0
DDX39A	ATP-dependen	O00148	49098	62	1
DDX39B	Spliceosome R	F8VQ10	50713	59	1
DDX3X	ATP-dependen	O00571	73198	218	2
DDX3Y	Uncharacterize	B4DXX7	72886	0	0
DDX41	Probable ATP-	J3KNN5	71603	0	0
DDX42	ATP-dependen	Q86XP3	102912	35	1
DDX46	Probable ATP-	H0Y9U3	117290	38	1
DDX47	Probable ATP-	Q9H0S4	50615	0	0
DDX5	Probable ATP-	J3KTA4	69044	233	6
DEK	Protein DEK (F	H0Y8X0	18540	0	0
DHCR7	7-dehydrochol	Q9UBM7	54454	35	1
DHX15	Putative pre-m	O43143	90875	1481	67
DHX30	Putative ATP-c	H7BXY3	130469	0	0
DHX37	Probable ATP-	F5H3Y4	107235	0	0
DHX9	ATP-dependen	Q08211	140869	0	0
DIABLO	Diablo homolo	F5GXT8	15243	66	1
DIDO1	Death-inducer	Q9BTC0	243723	0	0
DKC1	H/ACA ribonuc	O60832	57638	0	0
DKFZP586J061	DKFZP586J061	A4D212	265588	0	0
DKFZp781K13	NADH dehydro	Q5H9R2	13554	0	0
DLD	Dihydrolipoyl d	B4DHG0	43560	200	3

DNAI2	Dynein interme	J3QRG2	74800	32	1
DNAJA1	DnaJ homolog	P31689	44839	0	0
DNAJA2	DnaJ homolog	O60884	45717	36	1
DNAJA3	DnaJ homolog	E7ES32	33061	42	1
DNAJB11	DnaJ homolog	H7C2Y5	19346	0	0
DNAJB12	DnaJ (Hsp40) h	J3KPS0	45462	0	0
DNAJC10	DnaJ homolog	Q8IXB1	91021	35	0
DNAJC17	DnaJ homolog	Q9NVM6	34666	0	0
DNAJC8	DnaJ homolog	O75937	29823	666	24
DNAJC9	DnaJ homolog	Q8WXX5	29891	0	0
DNMT1	Cytosine-speci	F5GX68	183267	34	1
DOCK7	Dedicator of cy	H0Y7L2	147350	0	0
DPYSL5	Dihydropyrimid	E7EWB4	20853	0	0
DRAP1	Dr1-associatec	C9JCC6	23190	0	0
DROSHA	Ribonuclease 3	Q9NRR4	159214	0	0
DSG1	Desmoglein-1 (	Q02413	113676	0	0
DSP	Desmoplakin C	P15924	331569	0	0
DYNC1H1	Cytoplasmic dy	Q14204	532072	774	17
DYNLL2	Dynein light ch	Q96FJ2	10343	0	0
DYNLRB1	Dynein light ch	B1AKR6	16242	0	0
EBP	3-beta-hydroxy	Q15125	26336	0	0
ECHS1	Enoyl-CoA hyd	P30084	31367	79	2
ECI1	Enoyl-CoA delt	H3BS70	24689	54	1
EDC4	Enhancer of ml	Q6P2E9	151567	32	1
EDF1	Endothelial diff	O60869	16359	48	1
EEF1A1	Elongation fact	Q5JR01	15926	0	0
EEF1A1P5	Putative elonga	Q5VTE0	50153	28	2
EEF1A2	Elongation fact	Q05639	50438	429	12
EEF1B2	Elongation fact	P24534	24748	0	0
EEF1D	Elongation fact	H0YCK7	22934	0	0
EEF1G	Elongation fact	B4DTG2	56114	232	5
EEF2	Elongation fact	P13639	95277	22	0
EFTUD2	116 kDa U5 sm	K7EJ81	108215	170	4
EIF1AY	Eukaryotic tran	A6NJH9	14446	0	0
EIF3A	Eukaryotic tran	F5H335	162537	0	0
EIF3C	Eukaryotic tran	H3BRV0	104036	0	0
EIF3I	Eukaryotic tran	Q5TFK1	14073	0	0
EIF4A1	Eukaryotic initi	P60842	46125	0	1
EIF4A2	Eukaryotic initi	E7EQG2	41264	33	1

EIF4A3	Eukaryotic initi	P38919	46841	197	4
EIF4B	Eukaryotic tran	E7EX17	69657	0	0
EIF4H	Eukaryotic tran	Q15056	27368	82	2
EIF5A	Eukaryotic tran	I3L397	16108	203	5
EIF6	Eukaryotic tran	P56537	26582	0	0
ELAVL1	ELAV-like prot	B4DVB8	38972	182	3
EMD	Emerin OS=Ho	P50402	28976	0	0
ENDOV	Endonuclease '	E5RGZ9	13239	30	1
ENO1	Alpha-enolase	P06733	47139	193	4
ENO2	Enolase OS=H	F5H0C8	34741	0	0
ENY2	Transcription a	Q9NPA8	11521	0	0
EP400	E1A-binding pr	Q96L91	343276	32	1
EPB41L2	Band 4.1-like p	O43491	112519	0	0
EPN2	Epsin-2 OS=H	I3L2B2	61514	0	0
EPN3	Epsin-3 OS=H	B4DIQ9	28336	0	0
ERH	Enhancer of ru	P84090	12251	0	0
ERLIN1	Erlin-1 OS=Ho	O75477	38901	0	0
ERLIN2	Erlin-2 (Fragm	E5RHW4	37701	0	0
ERP29	Endoplasmic re	P30040	28975	75	1
ESYT1	Extended syna	Q9BSJ8	122780	0	0
ETFA	Electron transf	H0YK49	24145	92	2
ETFB	Electron transf	P38117	27826	82	2
EWSR1	RNA-binding p	B0QYK0	64889	4	1
EXOSC10	Exosome comp	Q01780	100768	0	0
EXOSC4	Exosome comp	E9PI41	28395	0	0
EXOSC5	Exosome comp	M0R050	21243	0	0
EXOSC6	Exosome comp	Q5RKV6	28218	0	0
EXOSC9	Exosome comp	D6RIY6	46948	0	0
EZR	Ezrin OS=Hom	P15311	69370	0	0
FAM124B	Protein FAM12	Q9H5Z6	50929	0	0
FAM184B	Protein FAM18	Q9ULE4	120969	0	0
FAM208A	Protein FAM20	Q9UK61	188914	0	0
FAM25A	Protein FAM25	B3EWG3	9314	0	0
FAM98A	Protein FAM98	B4DT23	34066	0	0
FANCD2	Fanconi anemi	Q9BXW9	166356	0	0
FANCI	Fanconi anemi	H3BP78	122868	0	0
FARP2	FERM, RhoGEF	F5GZ84	72217	33	1
FASN	Fatty acid synt	P49327	273254	40	1
FAU	40S ribosomal	E9PR30	10898	0	0

FBL	rRNA 2~-O-me	M0QXL5	26623	34	1
FBLL1	rRNA/tRNA 2~	R4GMW7	34782	0	0
FEN1	Flap endonucle	F5H1Y3	18263	0	0
FHL1	Four and a half	Q5JXH7	23310	0	0
FIP1L1	Pre-mRNA 3~-	Q6UN15	66487	0	0
FKBP8	Peptidyl-prolyl	J3KQ73	47114	0	0
FLG	Filaggrin OS=H	P20930	434922	0	0
FLG2	Filaggrin-2 OS=	Q5D862	247928	0	0
FLII	Protein flightle	Q13045	144659	0	0
FLNA	Filamin-A OS=	Q5HY54	276378	364	9
FLNB	Filamin-B OS=	O75369	277990	0	0
FLOT1	Flotillin-1 (Frag	A2AB09	27310	0	0
FLOT2	Flotillin-2 OS=	J3QLD9	47113	0	0
FMR1	Fragile X ment	G8JL90	68924	0	0
FNBP4	Formin-binding	Q8N3X1	110198	0	0
FNDC1	Fibronectin typ	J3KNQ2	194424	0	0
FUBP1	Far upstream e	B4DT31	69821	143	3
FUBP3	Far upstream e	Q96I24	61602	0	0
FUS	RNA-binding p	P35637	53465	533	17
FXR1	Fragile X ment	B4DXZ6	68285	0	0
FXR2	Fragile X ment	P51116	74178	0	0
GABRA4	Gamma-amino	D6R924	4265	0	0
GANAB	Neutral alpha-g	Q14697	106807	355	9
GAPDH	Glyceraldehyde	E7EUT5	36030	204	6
GATAD2B	Transcriptional	Q8WXI9	65220	0	0
GCN1L1	Translational a	Q92616	292572	256	7
GDI2	Rab GDP disso	E7EU23	51148	0	0
GEMIN5	Gem-associate	Q8TEQ6	168483	0	0
GFAP	Glial fibrillary a	P14136	49850	0	0
GIGYF2	PERQ amino ac	I1E4Y6	152434	42	1
GK	Glycerol kinase	H7C2A0	13093	39	1
GK3P	Putative glycer	Q14409	60559	0	0
GLO1	Lactoylglutathi	Q04760	20764	0	0
GLOD4	Glyoxalase don	I3L3Q4	25484	0	0
GNA12	Guanine nucle	Q03113	44251	42	1
GNAI2	Guanine nucle	P04899	40425	0	0
GNAS	Guanine nucle	Q5JWF2	110956	42	1
GNB1	Guanine nucle	B1AKQ8	12277	40	1
GNB2	Guanine nucle	E7EP32	32388	0	0

GNB2L1	Guanine nucle	D6R909	35055	84	2
GNG12	Guanine nucle	Q9UBI6	8001	0	0
GNG5	Guanine nucle	P63218	7314	0	0
GNL3	Guanine nucle	Q9BVP2	61954	0	0
GNL3L	Guanine nucle	Q9NVN8	65532	0	0
GOT2	Aspartate amir	E7ERW2	47487	116	3
GPI	Glucose-6-pho	K7EQ48	53368	131	3
GRM1	Metabotropic g	F8W805	101407	0	0
GSDMA	Gasdermin-A (	J3KRG2	49334	0	0
GSTO1	Glutathione S-	Q5TA02	23326	38	1
GSTP1	Glutathione S-	A8MX94	19468	0	0
GTF2I	General transc	P78347	112346	43	1
GTF3C1	General transc	Q12789	238725	0	0
GTPBP4	Nucleolar GTP	Q9BZE4	73918	0	0
GXYLT1	Glucoside xylo	Q4G148	50534	0	0
H1F0	Histone H1.0 C	P07305	20850	0	0
H1FX	Histone H1x O	Q92522	22474	0	0
H2AFJ	Histone H2A (F	H0YFX9	9970	0	0
H2AFX	Histone H2AX	P16104	15135	0	0
H2AFY	Core histone m	O75367	39592	0	0
H2AFY2	Core histone m	Q9P0M6	40033	0	0
H2AFZ	Histone H2A.Z	P0C0S5	13545	99	3
H3F3A	Histone H3 OS	B4DEB1	14044	8	0
H3F3B	Histone H3 (Fr	K7EK07	14905	0	0
HADHB	3-ketoacyl-Co/	B4E2W0	48848	0	0
HBA2	HCG1745306, i	G3V1N2	11940	0	0
HBE1	Hemoglobin su	A8MUF7	9464	0	0
HCFC1	HCF N-termina	A6NEM2	213343	0	0
HCK	Tyrosine-prote	H0Y3C5	59473	0	0
HDAC2	Histone deacet	B3KRS5	51965	0	0
HDAC4	Histone deacet	F5GX36	72986	33	1
HEATR1	HEAT repeat-c	Q5T3Q7	233127	0	0
HEXA	Beta-hexosami	H3BP20	61960	0	0
HINT2	Histidine triad	Q9BX68	17151	0	0
HIST1H1A	Histone H1.1 C	Q02539	21829	0	0
HIST1H1C	Histone H1.2 C	P16403	21352	0	0
HIST1H1D	Histone H1.3 C	P16402	22336	0	0
HIST1H1E	Histone H1.4 C	P10412	21852	0	0
HIST1H2AA	Histone H2A ty	Q96QV6	14225	0	0

HIST1H2AG	Histone H2A ty	P0C0S8	14083	228	7
HIST1H2BJ	Histone H2B ty	P06899	13896	0	0
HIST1H2BL	Histone H2B ty	Q99880	13944	0	0
HIST1H2BN	Histone H2B O	U3KQK0	18792	142	4
HIST1H4A	Histone H4 OS	P62805	11360	0	0
HIST2H2AB	Histone H2A ty	Q8IUE6	13987	0	0
HIST2H3PS2	Histone H3 OS	Q5TEC6	15421	0	0
HIST3H2BB	Histone H2B ty	Q8N257	13900	0	0
HMGA1	High mobility g	H7BYM6	34274	0	0
HMGB1	High mobility g	Q5T7C4	18299	0	0
HMGB1P1	Putative high n	B2RPK0	24223	34	1
HMGB2	High mobility g	D6R9A6	15394	0	0
HNRNPA0	Heterogeneous	Q13151	30822	99	8
HNRNPA1	Heterogeneous	F8W6I7	33135	752	39
HNRNPA2B1	Heterogeneous	P22626	37407	132	63
HNRNPA3	Heterogeneous	P51991	39571	0	28
HNRNPAB	Heterogeneous	D6R9P3	30284	0	0
HNRNPC	Heterogeneous	G3V4C1	33550	391	10
HNRNPD	Heterogeneous	Q14103	38410	763	22
HNRNPDL	Heterogeneous	O14979	46409	57	11
HNRNPF	Heterogeneous	P52597	45643	0	0
HNRNPH1	Heterogeneous	D6RBM0	51197	279	30
HNRNPH2	Heterogeneous	P55795	49232	0	2
HNRNPH3	Heterogeneous	B4DHY1	36903	158	20
HNRNPK	Heterogeneous	P61978	50944	642	15
HNRNPL	Heterogeneous	P14866	64092	58	2
HNRNPM	Heterogeneous	M0QZM1	77464	386	10
HNRNPR	Heterogeneous	O43390	70899	553	12
HNRNPU	Heterogeneous	Q00839	90528	224	7
HNRNPUL1	Heterogeneous	B7Z4B8	95679	634	20
HNRNPUL2	Heterogeneous	Q1KMD3	85052	636	15
HP1BP3	Heterochromat	Q5SSJ5	61169	0	0
HRNR	Hornerin OS=H	Q86YZ3	282228	0	0
HSD17B10	3-hydroxyacyl-	Q99714	26906	49	1
HSD17B12	Estradiol 17-be	Q53GQ0	34302	162	4
HSD17B4	Hydroxysteroid	G5E9S2	64552	220	4
HSP90AA1	Heat shock prc	P07900	84607	292	6
HSP90AB1	Heat shock prc	P08238	83212	0	0
HSP90B1	Endoplasmin C	P14625	92411	197	3

HSPA1A	Heat shock 70	P08107	70009	0	1
HSPA1L	Heat shock 70	P34931	70331	0	3
HSPA4	Heat shock 70	P34932	94271	56	1
HSPA5	78 kDa glucose	P11021	72288	1097	21
HSPA6	Heat shock 70	P17066	70984	100	4
HSPA8	Heat shock cog	P11142	70854	815	22
HSPA9	Stress-70 prote	P38646	73635	489	12
HSPB1	Heat shock pro	F8WE04	20394	0	0
HSPD1	60 kDa heat sh	P10809	61016	303	5
HSPE1	10 kDa heat sh	P61604	10925	89	2
HTRA1	Serine proteas	Q92743	51255	0	0
IGHG2	Ig gamma-2 ch	P01859	35878	0	0
IGLC2	Ig lambda-2 ch	P0CG05	11287	0	0
IGLL5	Immunoglobuli	B9A064	23049	1	0
IK	Protein Red OS	Q13123	65562	0	0
ILF2	Interleukin enh	Q12905	43035	590	15
ILF3	Isoform 7 of Int	Q12906-7	95748	1098	25
IMMT	Mitochondrial i	B9A067	78925	0	0
IMP3	U3 small nucle	Q9NV31	21837	0	0
IMP4	U3 small nucle	H0Y714	31926	0	0
INF2	Inverted formir	Q27J81	135540	0	0
ING5	Inhibitor of gro	Q8WYH8	27733	0	0
IQGAP2	Ras GTPase-ac	Q13576	180465	0	0
IREB2	Iron-responsive	P48200	104978	74	3
IRS4	Insulin recepto	O14654	133685	0	0
JUP	Junction plakog	F5GWP8	81693	0	0
KATNAL2	Katanin p60 AT	K7EIJ8	38097	51	1
KBTBD3	Kelch repeat ar	G3V161	60637	1	1
KDELR1	ER lumen prote	P24390	24526	0	0
KDELR2	ER lumen prote	H7BYF7	4124	0	0
KHDRBS1	KH domain-cor	Q07666	48197	402	11
KHSRP	Far upstream e	Q92945	73070	58	1
KIAA0430	Meiosis arrest	G8JLP4	174865	0	0
KIAA1429	Protein virilizer	Q69YN4	201898	0	0
KLRC4-KLRK1	Protein KLRC4	H3BQV0	17268	34	1
KM-PA-2	Ribosome biog	Q96Q25	71885	0	0
KPNA6	Importin subun	F5GYL8	60556	0	0
KPNB1	Importin subun	Q14974	97108	0	0
KPRP	Keratinocyte pr	Q5T749	64093	0	0

KTN1	Kinectin OS=H	Q86UP2	156179	0	0
LAMA5	Laminin subun	O15230	399479	33	1
LANCL1	LanC-like prote	E9PHS0	22028	50	1
LAS1L	Ribosomal biog	Q9Y4W2	83013	0	0
LBR	Lamin-B recep	Q14739	70658	0	0
LCN1	Lipocalin-1 OS	P31025	19238	134	2
LDHA	L-lactate dehy	F5GXY2	36665	107	3
LDHAL6B	L-lactate dehy	Q9BYZ2	41916	0	0
LDHB	L-lactate dehy	P07195	36615	131	3
LEMD2	LEM domain-c	D6R958	5903	0	0
LEPRE1	Prolyl 3-hydrox	Q32P28	83341	275	7
LGALS3BP	Galectin-3-bin	Q08380	65289	0	0
LGALS7	Galectin-7 OS=	P47929	15066	0	0
LIG3	DNA ligase 3 C	P49916	112835	32	1
LIMA1	LIM domain an	Q9UHB6	85173	14	1
LIN7A	Protein lin-7 h	H0YI92	14130	0	0
LINC00479	Putative uncha	Q96M42	15199	0	0
LMNA	Prelamin-A/C (	P02545	74095	149	3
LMNB1	Lamin-B1 OS=	P20700	66368	461	9
LMNB2	Lamin B2, isof	J9JID7	69906	255	6
LOC646903	LOC646903 pr	Q96EG4	15828	0	0
LRMP	Processed lym	F5H006	50394	0	0
LRPPRC	Leucine-rich P	B8ZZ38	56254	0	0
LRRC59	Leucine-rich re	Q96AG4	34909	0	0
LSM6	U6 snRNA-ass	P62312	9122	62	1
LUC7L	LUC7-like (S. c	A8MYV2	32428	0	0
LUC7L2	Putative RNA-b	Q9Y383	46486	77	2
LUC7L3	Cisplatin resist	J3KPP4	58185	0	0
LYAR	Cell growth-re	Q9NX58	43588	0	0
LYZ	Lysozyme C OS	F8VV32	11481	0	0
MAGEC1	Melanoma-ass	O60732	123568	0	0
MAGOH	Mago-nashi ho	B1ARP8	12836	46	1
MAGOHB	Protein mago r	Q96A72	17265	0	0
MAGT1	Magnesium tra	Q9H0U3	38011	0	0
MAP1A	MAP1 light cha	J3KPX8	305484	0	0
MAP1B	Microtubule-as	P46821	270468	68	2
MAP3K9	Mitogen-activa	G3V347	92769	0	0
MAPKAPK2	MAP kinase-ac	P49137	45538	0	0
MARS	Methionine--tF	H0YHV5	26681	0	0

MATR3	Matrin-3 OS=H	A8MXP9	99905	588	12
MBD3	Methyl-CpG bi	K7EIE8	26306	0	0
MCM3	DNA replicatio	B4DWW4	95848	0	0
MCM7	DNA replicatio	P33993	81257	0	0
MCOLN3	Mucolipin 3, is	B1ANB7	37219	0	0
MCTS1	Malignant T-ce	Q9ULC4	20542	0	0
MDC1	Mediator of DN	Q14676	226529	0	0
MDH2	Malate dehydr	P40926	35481	237	4
MDN1	Midasin OS=H	Q9NU22	632420	171	4
MEN1	Menin OS=Hor	E7EN32	61381	0	0
METTL3	N6-adenosine-	B4DTN4	27585	35	1
MEX3A	RNA-binding p	A1L020	54139	0	0
MFAP1	Microfibrillar-a	P55081	51927	0	0
MGST1	Microsomal glu	F5H760	11886	0	0
MGST2	Microsomal glu	Q99735	16610	0	0
MGST3	Microsomal glu	Q5VV89	18405	0	0
MLEC	Malectin (Frag	H0YG07	19722	0	0
MMTAG2	Multiple myelo	Q9BU76	29394	0	0
MOGS	Mannosyl-oligo	Q13724	91861	0	0
MOV10	Mov10, Molone	Q5JR04	107142	0	0
MRE11A	Double-strand	B3KTC7	81020	0	0
MRPL12	39S ribosomal	P52815	21335	0	0
MRPL22	39S ribosomal	J3KQY1	26458	0	0
MRPS15	28S ribosomal	P82914	29823	0	0
MRPS16	28S ribosomal	B4E032	13820	0	0
MRPS2	28S ribosomal	Q5T8A0	30460	0	0
MRPS31	28S ribosomal	Q92665	45290	0	0
MRTO4	mRNA turnove	Q9UKD2	27543	0	0
MSH2	DNA mismatch	P43246	104677	35	1
MSH3	DNA mismatch	P20585	127332	0	0
MSH6	DNA mismatch	B4DF41	137870	0	0
MT-ATP6	ATP synthase s	P00846	24801	0	0
MT-CO2	Cytochrome c o	P00403	25548	37	1
MT-ND1	NADH-ubiquin	P03886	35637	0	0
MTA1	Metastasis-ass	E7ESY4	79323	0	0
MTA2	Metastasis-ass	O94776	74976	0	0
MTCH2	Mitochondrial c	E9PIE4	28644	0	0
MTX2	Metaxin 2 OS=	Q8IZ68	28805	0	0
MYADM	Myeloid-associ	C9J5M0	26222	0	0

MYBBP1A	Myb-binding pı	I3L1L3	140155	0	0
MYCBP	C-Myc-binding	Q99417	11959	0	0
MYEF2	Myelin express	Q9P2K5	64081	42	1
MYH10	Myosin-10 OS=	P35580	230635	0	0
MYH11	Myosin-11 OS=	P35749	227199	0	0
MYH14	Myosin-14 OS=	F2Z2U8	231075	0	0
MYH7	Myosin-7 OS=I	P12883	222959	0	0
MYH9	Myosin-9 OS=I	P35579	226392	0	0
MYL12A	Myosin regulat	J3QRS3	20444	36	1
MYL6	Myosin light pc	G3V1V0	18026	0	0
MYO1B	Unconventiona	E9PDF6	128400	0	0
MYO1C	Unconventiona	F5H6E2	118920	0	0
MYO1D	Unconventiona	O94832	116129	0	0
MYO6	Unconventiona	E7EW20	148742	0	0
NACA	Nascent polype	H0YHX9	22930	0	0
NAP1L1	Nucleosome as	H0YH88	21065	0	0
NAT10	N-acetyltransfe	Q9H0A0	115657	0	0
NAT14	N-acetyltransfe	M0R1E3	17831	0	0
NBAS	Neuroblastoma	H0Y5G7	160425	0	0
NCAPD3	Condensin-2 c	P42695	168783	0	0
NCBP1	Nuclear cap-bi	Q09161	91781	0	0
NCCRP1	F-box only prot	Q6ZVX7	30828	0	0
NCL	Nucleolin OS=I	P19338	76568	975	17
NCOA5	Nuclear recept	Q9HCD5	65496	0	0
NDUFA12	NADH dehydro	H0YID5	6148	0	0
NDUFA13	NADH dehydro	J3KN00	25804	0	0
NDUFA2	NADH dehydro	O43678	10915	0	0
NDUFA4	NADH dehydro	O00483	9364	0	0
NDUFA6	NADH dehydro	R4GN43	8504	0	0
NDUFS2	NADH dehydro	B7Z9L2	26953	0	0
NDUFS3	NADH dehydro	O75489	30223	93	1
NDUFS7	NADH dehydro	B3KRI2	22188	0	0
NDUFS8	NADH dehydro	F8W9K7	13844	0	0
NDUFV1	NADH dehydro	G3V0I5	50022	0	0
NDUFV2	NADH dehydro	E7EPT4	27889	0	0
NEK10	Serine/threonii	Q6ZWH5	133176	0	0
NHP2	H/ACA ribonuc	Q9NX24	17190	0	0
NHP2L1	NHP2-like prot	P55769	14165	57	1
NIFK	MKI67 FHA do	Q9BYG3	34201	0	0

NIP7	60S ribosome s	J3QLW7	12445	0	0
NIPBL	Nipped-B-like	Q6KC79	315854	0	0
NKRF	NF-kappa-B-re	O15226	77624	123	2
NLE1	Notchless prot	K7ERN7	13709	0	0
NLRC3	Protein NLRC3	B5MDB6	111493	0	0
NME1	Nucleoside dip	P15531	17138	0	0
NME1-NME2	Nucleoside dip	Q32Q12	32621	142	4
NME2	Nucleoside dip	P22392	17287	0	0
NME3	Nucleoside dip	Q13232	19003	30	1
NME4	Nucleoside dip	F2Z2X0	25444	0	0
NMNAT1	Nicotinamide n	B1AN62	18299	0	0
NO66	Bifunctional lys	Q9H6W3	71041	0	0
NOC2L	Nucleolar comp	Q9Y3T9	84866	0	0
NOC3L	Nucleolar comp	F5H677	57315	0	0
NOC4L	Nucleolar comp	Q9BVI4	58431	0	0
NOL11	Nucleolar prote	Q9H8H0	81072	0	0
NOL6	Nucleolar prote	G8JLK7	121957	0	0
NOLC1	Nucleolar and c	S4R341	8046	0	0
NONO	Non-POU dom	Q15233	54197	111	5
NOP16	Nucleolar prote	D6RIC3	17187	0	0
NOP2	Putative riboso	P46087	89247	0	0
NOP56	Nucleolar prote	O00567	66009	0	0
NOP58	Nucleolar prote	Q9Y2X3	59541	0	0
NOVA2	RNA-binding p	Q9UNW9	48979	0	0
NPM1	Nucleophosmin	P06748	32555	249	6
NPM3	Nucleoplasmin	O75607	19331	0	0
NR2F1	COUP transcrip	P10589	46126	0	0
NSDHL	Sterol-4-alpha-	C9JDR0	28128	0	0
NUDT16L1	Protein syndes	Q9BRJ7	23323	91	3
NUDT21	Cleavage and p	O43809	26211	662	12
NUMA1	Isoform 2 of Nu	Q14980-2	236372	0	0
NUP107	Nuclear pore co	B4DZ67	103109	0	0
NUP133	Nuclear pore co	F5H5C2	127262	0	0
NUP153	Nuclear pore co	F6QR24	157240	0	0
NUP155	Nuclear pore co	E9PF10	148000	0	0
NUP160	Nuclear pore co	G3V198	140198	0	0
NUP188	Nucleoporin NU	Q5SRE5	195917	0	0
NUP205	Nuclear pore co	Q92621	227776	0	0
NUP210	Nuclear pore m	Q8TEM1	204983	71	2

NUP214	Nuclear pore c	P35658	213488	0	0
NUP35	Nucleoporin NU	B4DP57	21129	0	0
NUP37	Nucleoporin Nu	Q8NFH4	36684	0	0
NUP43	Nucleoporin Nu	Q8NFH3	42124	0	0
NUP85	Nuclear pore c	J3KT10	70133	0	0
NUP93	Nuclear pore c	H3BVG0	99492	0	0
NUP98	Nuclear pore c	J3KP29	177290	0	0
NUPL1	Nucleoporin p5	Q5JRG1	49065	0	0
NXF1	Nuclear RNA e	Q9UBU9	70139	0	0
OLA1	Obg-like ATPa	J3KQ32	46908	0	0
ORC4	Origin recognit	O43929	50345	0	0
ORC5	Origin recognit	O43913	50251	0	0
OSBPL8	Oxysterol-bind	F8VQX7	81606	0	0
OTUD4	OTU domain co	G3V0I6	123968	0	0
P4HB	Protein disulfic	P07237	57081	266	8
PA2G4	Proliferation-a	Q9UQ80	43759	93	2
PABPC1	Polyadenylate-	E7ERJ7	67096	33	1
PABPC4	Poly(A) binding	B1ANR0	67928	0	0
PABPN1	Polyadenylate-	G3V4T2	20230	38	1
PAK1IP1	p21-activated p	Q9NWT1	43936	0	0
PARK7	Protein DJ-1 O	K7ELW0	17898	0	0
PARP1	Poly [ADP-ribo	P09874	113012	73	1
PARP10	Poly [ADP-ribo	E9PK67	109029	0	0
PAXBP1	PAX3- and PAX	Q9Y5B6	104739	0	0
PBRM1	Protein polybro	H0Y5B5	126213	0	0
PCBP1	Poly(rC)-bindir	Q15365	37474	30	0
PCBP2	Poly(RC) bindi	G3V0E8	33475	0	0
PCID2	PCI domain-co	Q5JVF3	46000	0	0
PCMT1	Protein-L-isoa	J3KP72	30296	28	0
PCNP	PEST proteolyt	Q8WW12	18913	433	12
PDCD11	Protein RRP5 H	Q14690	208570	0	0
PDCD6	Programmed c	O75340	21855	0	0
PDIA3	Protein disulfic	G5EA52	54929	341	7
PDIA4	Protein disulfic	P13667	72887	32	0
PDIA6	Protein disulfic	B5MCQ5	53228	0	0
PDS5A	Sister chromati	Q29RF7	150734	0	0
PDS5B	Sister chromati	Q9NTI5	164563	0	0
PEBP1	Phosphatidylet	P30086	21044	0	0
PELP1	Proline-, glutar	I3L3A8	124865	31	1

PES1	Pescadillo hom	B5MCF9	66036	0	0
PEX6	Peroxin Pex6p	Q8WYQ0	77694	0	0
PFKFB3	6-phosphofruc	H0Y483	16566	0	0
PFKP	6-phosphofruc	B1APP6	26054	34	1
PFN1	Profilin-1 OS=I	P07737	17506	37	0
PGAM1	Phosphoglycer	P18669	28786	38	1
PGK1	Phosphoglycer	B7Z7A9	41402	133	3
PGRMC1	Membrane-ass	B7Z1L3	15869	0	0
PHB	Prohibitin (Fra	E7ESE2	22385	189	4
PHB2	Prohibitin-2 OS	J3KPX7	33382	272	6
PHF5A	PHD finger-lik	Q7RTV0	12397	78	9
PHGDH	D-3-phosphogl	Q5SZU1	53051	136	3
PHIP	PH-interacting	Q8WWQ0	206560	0	0
PIN4	Peptidyl-prolyl	H0Y4T6	9537	0	0
PIP	Prolactin-induc	P12273	16562	0	0
PKM	Pyruvate kinas	H3BQ34	30701	0	0
PKP1	Plakophilin-1 C	Q13835	82808	0	0
PLA2G4B	Cytosolic phos	P0C869	87922	0	0
PLEC	Plectin OS=Ho	Q15149	531466	0	0
PLOD1	Procollagen-ly	B4DR87	88217	246	5
PLRG1	Pleiotropic reg	A8MW61	57146	0	0
PNN	Pinin OS=Hom	Q9H307	81565	0	0
POGZ	Pogo transpos	Q7Z3K3	155245	0	0
POLDIP3	Polymerase de	B4DMM2	20211	0	0
POLG2	DNA polymera	J3KRM2	17673	0	0
POLR2A	DNA-directed I	P24928	217039	125	3
POLR2C	DNA-directed I	P19387	31422	0	0
POLR2E	DNA-directed I	P19388	24536	0	0
POLR2G	DNA-directed I	P62487	19282	0	0
POLR2K	DNA-directed I	P53803	6999	0	0
PPA1	Inorganic pyro	Q5SQT6	19969	0	0
PPA2	Inorganic pyro	H0Y9D8	25619	0	0
PPIA	Peptidyl-prolyl	P62937	18001	157	4
PPIB	Peptidyl-prolyl	P23284	23728	72	0
PPIF	Peptidyl-prolyl	P30405	22026	163	4
PPIH	Peptidyl-prolyl	C9JQD4	15790	0	0
PPIL1	Peptidyl-prolyl	Q9Y3C6	18225	0	0
PPIL3	Peptidyl-prolyl	H7BZ14	20259	0	0
PPP1CA	Serine/threoni	E9PMD7	28879	0	0

PPP1CB	Serine/threonii	P62140	37163	0	0
PPP1CC	Serine/threonii	F8VYE8	34927	0	0
PPP1R9B	Neurabin-2 OS	Q96SB3	89138	0	0
PPP2CB	Serine/threonii	H0YBN9	6093	32	1
PPP2R1A	Serine/threonii	B3KQV6	45575	0	0
PPP6R2	Serine/threonii	T1ECW5	72922	32	1
PQBP1	Polyglutamine-	H7C053	16556	0	0
PRAF2	PRA1 family pr	A6NP52	17177	56	1
PRC1	Protein regulat	H0YL53	23061	5	3
PRDX1	Peroxiredoxin-	Q06830	22096	0	0
PRDX2	Peroxiredoxin-:	P32119	21878	13	1
PRDX3	Thioredoxin-de	E9PH29	25822	1	0
PRDX4	Peroxiredoxin-·	Q13162	30521	163	4
PRDX6	Peroxiredoxin-(	P30041	25019	48	1
PRKCSH	Glucosidase 2 :	K7ELL7	60154	77	1
PRKDC	DNA-depender	P78527	468788	753	16
PRMT5	Protein arginin	G3V5W5	67631	85	2
PRPF19	Pre-mRNA-pro	Q9UMS4	55146	171	4
PRPF3	U4/U6 small nı	B4DSY9	31869	30	1
PRPF31	U4/U6 small nı	E7EN72	55421	0	0
PRPF39	Pre-mRNA-pro	Q86UA1	78381	15	0
PRPF4	U4/U6 small nı	O43172	58412	0	0
PRPF40A	Pre-mRNA-pro	O75400	108737	0	0
PRPF6	Pre-mRNA-pro	O94906	106858	48	1
PRPF8	Pre-mRNA-pro	Q6P2Q9	273427	1858	42
PRSS3	Trypsin-3 (Fraį	B1AN99	19389	0	0
PRSS33	Serine proteas	I3L3D7	25382	0	0
PSAP	Saposin-D OS=	B1AVU8	61652	0	0
PSIP1	PC4 and SFRS	O75475	60067	0	0
PSMA1	Proteasome su	F5GX11	26488	31	1
PSMA3	Proteasome su	P25788	28415	82	1
PSMA4	Proteasome su	H0YL69	26360	0	0
PSMB5	Proteasome su	H0YJM8	28462	115	2
PSMB6	Proteasome su	P28072	25341	0	0
PSMB7	Proteasome su	Q99436	29946	0	0
PSPC1	Paraspeckle cc	Q8WXF1	58706	0	0
PTBP1	Polypyrimidine	P26599	57186	183	4
PTBP2	Polypyrimidine	B4DSS8	59583	0	0
PTGES3	Prostaglandin I	B4DDC6	14563	0	0

PTMA	Thymosin alph	H7C2N1	15849	52	1
PTPLAD1	Very-long-chai	B4DRF4	36407	0	0
PTPN13	Tyrosine-prote	Q12923	276733	0	0
PUF60	Poly(U)-bindin	Q9UHX1	59838	0	0
PWP2	Periodic trypto	Q15269	102387	0	0
RAB10	Ras-related pr	P61026	22527	142	3
RAB15	Ras-related pr	P59190	24375	91	2
RAB33B	Ras-related pr	Q9H082	25701	31	1
RAB35	Ras-related pr	F5H157	21201	91	2
RAB4B	HCG1995540, i	Q6PIK3	13528	0	0
RAB5C	Ras-related pr	P51148	23468	126	2
RAB6A	Ras-related pr	H0YGL6	22746	31	1
RAB7A	Ras-related pr	C9IZZ0	13067	0	0
RAB8B	Ras-related pr	H0YMN7	10478	0	0
RAC2	Ras-related C3	B1AH77	16765	0	0
RAD50	DNA repair pro	Q92878	153797	85	2
RAD52	Isoform beta o	P43351-2	24524	0	0
RAE1	mRNA export f	E9PQ57	47801	0	0
RALY	RNA-binding p	Q5QPL9	24650	125	3
RAN	GTP-binding n	J3KQE5	26799	94	3
RANBP1	Ran-specific G	P43487	23296	0	0
RANBP2	E3 SUMO-prot	P49792	357974	0	0
RANGAP1	Ran GTPase-a	H0Y4Q3	26891	0	0
RAP1B	Ras-related pr	F5GX62	15737	34	1
RAPGEF3	Rap guanine n	F8VRX1	92093	0	0
RAPH1	Ras associatio	C9K0J5	141091	0	0
RB1CC1	RB1-inducible	Q8TDY2	182975	0	0
RBBP4	Histone-bindin	Q09028	47626	121	3
RBBP5	Retinoblastom	Q15291	59116	0	0
RBBP7	Histone-bindin	E9PC52	46909	0	0
RBM10	RNA-binding p	P98175	103469	990	26
RBM12B	RNA-binding p	B9ZVT1	102641	0	0
RBM14	RNA-binding p	Q96PK6	69449	405	9
RBM15	Putative RNA-	Q96T37	107124	52	1
RBM17	Splicing factor	Q96I25	44934	1140	36
RBM20	RNA-binding p	Q5T481	134274	0	0
RBM25	RNA-binding p	P49756	100124	0	0
RBM3	Putative RNA-	P98179	17160	0	0
RBM39	RNA-binding p	E1P5S2	40516	92	2

RBM4	RNA-binding p	E9PB51	26270	0	0
RBM45	RNA-binding p	Q8IUH3	53469	0	0
RBM5	RNA-binding p	P52756	92097	1226	27
RBM6	RNA-binding p	P78332	128565	410	10
RBM8A	RNA-binding p	Q9Y5S9	19877	76	1
RBMX	RNA-binding m	P38159	42306	563	17
RBMXL1	RNA binding m	Q96E39	42116	237	6
RBP3	Retinol-binding	P10745	135278	30	1
RCC1	Regulator of ch	C9JW69	39559	31	0
RCC2	Protein RCC2 (	Q9P258	56049	40	1
RCL1	RNA 3~-termir	Q9Y2P8	40817	0	0
RCN1	Reticulocalbin-	Q15293	38866	0	0
RCN2	Reticulocalbin-	F8WCY5	39115	0	0
RCOR3	REST corepres	B4DV59	41776	0	0
RECQL	ATP-dependen	P46063	73410	40	1
RER1	Protein RER1 (	Q5T091	21011	0	0
RFC1	Replication fac	P35251	128175	0	0
RFC2	Replication fac	P35250	39132	0	0
RFC3	Replication fac	P40938	40530	0	0
RFC4	Replication fac	C9JZI1	36854	0	0
RFC5	Replication fac	F8W9B4	28828	0	0
RGPD1	RANBP2-like a	P0DJD0	196539	57	1
RGPD3	RanBP2-like ar	J3KNE0	198270	0	0
RHCE	Blood group Rh	H0YCJ8	8389	34	1
RIF1	Telomere-asso	Q5UIP0	274294	0	0
RNPS1	RNA-binding p	H3BMM9	31681	0	0
ROCK2	Rho-associated	D6REE7	50695	0	0
RP1	Oxygen-regulat	P56715	240511	33	1
RPA1	Replication pro	I3L2M5	16262	0	0
RPA2	Replication pro	Q5TEJ7	19421	0	0
RPA3	Replication pro	B5MC59	9165	0	0
RPF2	Ribosome prod	Q9H7B2	35560	0	0
RPL10	60S ribosomal	F8W7C6	18580	0	0
RPL10A	60S ribosomal	P62906	24816	40	1
RPL11	60S ribosomal	P62913	20240	65	1
RPL12	60S ribosomal	P30050	17808	79	2
RPL13	60S ribosomal	P26373	24247	0	0
RPL13A	60S ribosomal	M0QYS1	24201	0	0
RPL14	60S ribosomal	E7EPB3	14549	0	0

RPL15	60S ribosomal	P61313	24131	0	0
RPL17	60S ribosomal	J3KRX5	20158	0	0
RPL18	60S ribosomal	H0YHA7	18962	0	0
RPL18A	60S ribosomal	M0R117	18067	0	0
RPL19	Ribosomal prot	J3KTE4	23233	0	0
RPL21	60S ribosomal	P46778	18553	0	0
RPL22	60S ribosomal	K7ERI7	11162	6	0
RPL23	60S ribosomal	P62829	14856	85	2
RPL23A	60S ribosomal	P62750	17792	67	1
RPL24	60S ribosomal	C9JNW5	17532	0	0
RPL26	60S ribosomal	P61254	17248	0	0
RPL26L1	60S ribosomal	E5RIT6	15197	0	0
RPL27	60S ribosomal	P61353	15788	0	0
RPL27A	60S ribosomal	E9PLL6	12194	0	0
RPL28	60S ribosomal	P46779	15738	0	0
RPL3	60S ribosomal	P39023	46080	0	0
RPL30	60S ribosomal	E5RI99	12648	0	0
RPL31	60S ribosomal	B7Z4C8	15109	42	1
RPL34	60S ribosomal	P49207	13284	0	0
RPL35	60S ribosomal	F2Z388	10638	0	0
RPL35A	60S ribosomal	P18077	12530	35	1
RPL36A	60S ribosomal	H0Y5B4	15291	0	0
RPL37A	60S ribosomal	P61513	10268	0	0
RPL38	60S ribosomal	J3KT73	7560	41	1
RPL4	60S ribosomal	P36578	47667	0	0
RPL5	60S ribosomal	P46777	34341	0	0
RPL6	60S ribosomal	Q02878	32708	0	0
RPL7	60S ribosomal	P18124	29207	0	0
RPL7A	60S ribosomal	P62424	29977	0	0
RPL7L1	60S ribosomal	R4GMU7	22732	0	0
RPL8	60S ribosomal	P62917	28007	0	0
RPL9	60S ribosomal	H0Y9R4	10150	0	0
RPLP0	60S acidic ribo	P05388	34252	107	2
RPLP1	60S acidic ribo	P05386	11507	30	1
RPLP2	60S acidic ribo	H0YDD8	11658	137	3
RPN1	Dolichyl-dipho	P04843	68527	36	1
RPN2	Dolichyl-dipho	P04844	69241	0	0
RPP40	Ribonuclease F	O75818	41807	0	0
RPS10	40S ribosomal	P46783	18886	14	0

RPS10-NUDT3	Protein RPS10-	S4R435	32576	0	0
RPS11	40S ribosomal	P62280	18419	75	2
RPS12	40S ribosomal	P25398	14505	0	0
RPS13	40S ribosomal	P62277	17212	0	0
RPS14	40S ribosomal	P62263	16263	51	1
RPS15	40S ribosomal	K7ELC2	17712	0	0
RPS15A	40S ribosomal	P62244	14830	81	2
RPS16	40S ribosomal	M0R210	14410	93	2
RPS17	40S ribosomal	H0YK46	21629	73	2
RPS18	40S ribosomal	P62269	17708	141	2
RPS19	40S ribosomal	M0QXK4	16051	195	4
RPS2	40S ribosomal	H0YEN5	21140	0	0
RPS20	40S ribosomal	P60866	13364	39	1
RPS23	40S ribosomal	P62266	15798	0	0
RPS24	40S ribosomal	E7ETK0	15187	0	0
RPS25	40S ribosomal	P62851	13734	92	2
RPS26	40S ribosomal	P62854	13007	0	0
RPS27	40S ribosomal	Q5T4L4	7352	0	0
RPS28	40S ribosomal	P62857	7836	23	0
RPS29	40S ribosomal	P62273	6672	0	0
RPS3	40S ribosomal	P23396	26671	181	2
RPS3A	40S ribosomal	D6RG13	25591	0	0
RPS4X	40S ribosomal	P62701	29579	122	3
RPS5	40S ribosomal	M0QZN2	14754	88	1
RPS6	40S ribosomal	P62753	28663	0	0
RPS7	40S ribosomal	P62081	22113	86	2
RPS8	40S ribosomal	Q5JR95	21866	0	0
RPS9	40S ribosomal	P46781	22578	0	0
RPSA	40S ribosomal	C9J9K3	29487	0	0
RPSAP58	40S ribosomal	A6NE09	32888	48	1
RRNAD1	Protein RRNAD	Q4VX71	29655	0	0
RRP1	Ribosomal RN/	P56182	52807	0	0
RRP12	RRP12-like pro	F5H456	112965	0	0
RRP1B	Ribosomal RN/	Q14684	84375	0	0
RRP9	U3 small nucle	O43818	51809	0	0
RTCB	tRNA-splicing l	Q9Y3I0	55175	329	7
RTN1	Reticulon OS=l	A8MT72	21848	38	2
RTN3	Reticulon-3 OS	O95197	112541	78	3
RTN4	Reticulon OS=l	F8W914	37121	0	0

RUFY2	RUN and FYVE	H0YD93	71390	4	1
RUVBL1	RuvB-like 1 OS	Q9Y265	50196	99	2
RUVBL2	RuvB-like 2 OS	Q9Y230	51125	0	0
RYR1	Ryanodine rece	P21817	564815	0	0
S100A14	Protein S100-A	Q9HCY8	11655	0	0
S100A6	Protein S100-A	R4GN98	9675	0	0
S100A7	Protein S100-A	P31151	11464	0	0
S100A8	Protein S100-A	P05109	10828	79	2
SAFB	Scaffold attach	Q15424	102580	660	17
SAFB2	Scaffold attach	Q14151	107408	0	0
SAMM50	Sorting and as:	A8MZI2	28477	0	2
SAP18	Histone deacet	O00422	17550	48	2
SAP30BP	SAP30-binding	J3QQJ0	35862	0	0
SART1	U4/U6.U5 tri-s	O43290	90200	0	0
SATB2	DNA-binding p	Q3ZB87	69094	0	0
SCAMP1	Secretory carri	U3KQ30	37849	77	2
SCGB1D2	Secretoglobin f	O95969	9918	0	0
SCP2	Non-specific li	P22307	58956	0	0
SDHA	Succinate dehy	H0Y8X1	13628	0	0
SEC11A	SEC11-like 1 (S	H0YK72	18976	0	0
SEC22B	Vesicle-traffick	O75396	24578	89	2
SEC23A	Protein transpo	B3KXI2	63061	0	0
SEC61A2	Protein transpo	F2Z2C7	17860	0	0
SEC61B	Protein transpo	S4R3B5	4871	0	0
SEC63	Translocation p	Q9UGP8	87942	0	0
SEH1L	Nucleoporin SE	K7ELV2	29522	0	0
SEL1L	Protein sel-1 h	Q9UBV2	88698	0	0
2-Sep	Septin-2 OS=H	B5MCX3	36917	0	0
SERPINB4	Serpin B4 (Frag	H0Y5H9	42472	0	0
SET	Protein SET OS	Q01105	33469	142	2
SF1	Splicing factor	H7C0N4	80569	1158	28
SF3A1	Splicing factor	Q15459	88831	1062	42
SF3A2	Splicing factor	K7EMT0	49224	606	15
SF3A3	Splicing factor	Q12874	58812	1066	34
SF3B1	Splicing factor	O75533	145738	2543	89
SF3B14	Pre-mRNA bra	Q9Y3B4	14576	108	23
SF3B2	Splicing factor	Q13435	100165	1949	69
SF3B3	Splicing factor	I3L4G7	135492	1830	64
SF3B4	Splicing factor	Q15427	44357	237	7

SF3B5	Splicing factor	Q9BWJ5	10129	127	7
SFI1	Protein SFI1 hc	D3YTJ2	128933	0	0
SFN	14-3-3 protein	P31947	27757	0	0
SFPQ	Splicing factor,	P23246	76102	0	0
SFRS3	Serine/arginine	B4E241	14194	97	2
SFSWAP	Splicing factor,	Q12872	104758	40	1
SHMT2	Serine hydroxy	H0YIZ0	28800	0	0
SHQ1	Protein SHQ1 h	B4DL05	61510	0	0
SHROOM3	Protein Shroom	Q8TF72	216724	0	0
SIGMAR1	Sigma non-opi	Q99720	25112	0	0
SKIV2L2	Superkiller vira	F5H7E2	106746	0	0
SKP1	S-phase kinase	E5RJR5	18708	0	0
SLC12A7	Solute carrier f	Q9Y666	119029	0	0
SLC16A1	Monocarboxyla	Q5T8R3	31669	0	0
SLC22A5	Solute carrier f	H7C1R8	15573	0	0
SLC25A1	Tricarboxylate	P53007	33991	0	0
SLC25A10	Mitochondrial (	B4DLN1	48069	60	1
SLC25A11	Mitochondrial 2	I3L1P8	32162	48	1
SLC25A12	Calcium-bindir	B3KR64	62681	0	0
SLC25A24	Calcium-bindir	Q6NUK1	53320	0	0
SLC25A3	Phosphate carr	F8VVM2	36138	39	1
SLC25A5	ADP/ATP trans	P05141	32831	257	7
SLC25A6	ADP/ATP trans	P12236	32845	0	0
SLIRP	SRA stem-loop	H0YJ40	10843	33	1
SLTM	SAFB-like tran	H7BXE3	48819	0	0
SMARCA2	Probable globa	P51531	181166	0	0
SMARCA4	SMARCA4 isof	Q9HBD4	188031	47	1
SMARCA5	SWI/SNF-relat	O60264	121828	0	0
SMARCC1	SWI/SNF comp	Q92922	122790	0	0
SMARCC2	SWI/SNF comp	F8VXC8	136100	86	2
SMARCD1	SWI/SNF-relat	Q96GM5	58196	0	0
SMC1A	Structural mair	Q14683	143144	33	1
SMC1B	Structural mair	Q8NDV3	143818	0	0
SMC3	Structural mair	Q9UQE7	141454	0	0
SMC4	Structural mair	E9PD53	144365	220	5
SMCHD1	Structural mair	J3KTL8	155549	0	0
SMNDC1	Survival of mot	O75940	26694	106	2
SMU1	WD40 repeat-c	Q2TAY7	57507	0	0
SNAP23	Synaptosomal-	H3BM38	13897	0	0

SNRNP200	U5 small nucle	O75643	244353	1276	25
SNRNP40	U5 small nucle	Q96DI7	44488	61	1
SNRNP70	U1 small nucle	P08621	51526	0	0
SNRPA	U1 small nucle	M0QXK2	31259	112	3
SNRPA1	U2 small nucle	H0YMA0	28398	275	19
SNRPB2	U2 small nucle	P08579	25470	307	20
SNRPC	U1 small nucle	P09234	17381	0	0
SNRPD1	Small nuclear r	P62314	13273	301	9
SNRPD2	Small nuclear r	P62316	13518	203	15
SNRPD3	Small nuclear r	B4DJP7	13283	38	3
SNRPE	Small nuclear r	P62304	10797	116	4
SNRPF	Small nuclear r	P62306	9719	0	2
SNRPG	Small nuclear r	P62308	8490	101	4
SNRPN	Small nuclear r	B3KVR1	25059	214	8
SNW1	SNW domain-c	G3V3A4	65351	0	0
SNX9	Sorting nexin-9	Q9Y5X1	66550	0	0
SOD1	Superoxide dis	H7BYH4	13901	94	2
SON	Protein SON O	P18583	263664	0	0
SP140L	Nuclear body p	H7BYP4	46190	0	0
SPATA7	Spermatogenes	G3V287	9187	0	0
SPDEF	SAM pointed d	O95238	37494	0	0
SPEN	Msx2-interacti	F6WRY4	43002	0	0
SPIN1	Spindlin-1 OS=	Q9Y657	29582	0	0
SPIN2B	Spindlin-2B (F	Q5JZB8	27794	32	1
SPINK5	Serine proteas	Q9NQ38	120637	0	0
SPRR3	Small proline-r	B1AN48	17048	38	1
SPTA1	Spectrin alpha	P02549	279842	0	0
SPTAN1	Spectrin alpha	Q13813	284772	116	2
SPTBN1	Spectrin beta c	Q01082	274439	72	0
SPTBN2	Spectrin beta c	O15020	271157	0	0
SRM	Spermidine syr	K7EQ47	14421	0	0
SRP14	Signal recognit	H0YLA2	13050	0	0
SRP68	Signal recognit	Q9UHB9	70686	0	0
SRP72	Signal recognit	D6RDY6	39641	0	0
SRPRB	Signal recognit	Q9Y5M8	29684	0	0
SRRM2	Serine/arginine	Q9UQ35	299438	0	0
SRRT	Serrate RNA ef	Q9BXP5	100604	107	2
SRSF1	Serine/arginine	J3KTL2	28312	7	0
SRSF10	Serine/arginine	Q5JRI1	20900	5	0

SRSF2	Serine/arginine	J3KP15	15518	40	1
SRSF4	Serine/arginine	Q08170	56645	0	0
SRSF5	Serine/arginine	Q13243	31245	0	0
SRSF6	Serine/arginine	Q13247	39563	0	0
SRSF7	Serine/arginine	C9JAB2	26912	0	0
SRSF9	Serine/arginine	S4R3G0	25526	199	6
SSB	Lupus La prote	P05455	46808	0	0
SSBP1	Single-strande	E7EUY5	15703	0	0
SSR1	Translocon-ass	F5H5Y2	29402	0	0
SSR4	Translocon-ass	P51571	18987	0	0
SSRP1	FACT complex	Q08945	81024	0	0
STAG1	Cohesin subun	Q6P275	140001	0	0
STAG2	Cohesin subun	F8WAK8	133986	0	0
STK17A	Serine/threoni	Q9UEE5	46529	0	0
STK38	Serine/threoni	Q15208	54155	0	0
STK38L	Serine/threoni	I3L0D0	53968	0	0
STMN1	Stathmin OS=H	P16949	17292	134	2
STMN2	Stathmin OS=H	E5RGX5	19562	94	2
STOML2	Stomatin-like p	B4E1K7	33317	0	0
STRAP	Serine-threoni	B0AZV0	28488	0	0
STRBP	Spermatid peri	Q96SI9	73606	0	0
STRN4	Striatin-4 (Frag	R4GN16	34684	0	0
SUB1	Activated RNA	P53999	14386	0	0
SUGP1	SURP and G-pa	B4DVK3	72425	948	20
SUGP2	SURP and G-pa	M0R2Z9	121468	0	0
SUMO1	SMT3 suppress	B8ZZ67	7161	0	0
SUMO3	Small ubiquitin	B4DUW4	15758	0	0
SUN1	SUN domain-co	H0Y6N5	70999	0	0
SUN2	SUN domain-co	J3KQE0	79037	0	0
SUPT16H	FACT complex	Q9Y5B9	119838	0	0
SUPT6H	Transcription e	Q7KZ85	198949	0	0
SURF4	Surfeit 4 OS=H	Q5T8U5	21114	0	0
SYMPK	Symplekin OS=	Q92797	141059	0	0
SYNCRIP	Heterogeneous	O60506	69560	387	7
TAF15	TATA-binding p	Q92804	61793	230	11
TAGLN2	Transgelin-2 O	P37802	22377	0	0
TARDBP	TAR DNA-bind	Q13148	44711	39	1
TARS	Threonine--tRN	G3XAN9	72022	0	0
TAX1BP1	Tax1-binding p	B8ZZD4	93551	0	0

TBC1D8B	TBC1 domain f	Q0IIM8	128627	0	0
TBCA	Tubulin-specifi	B4DT30	15781	53	1
TBL3	Transducin bet	Q12788	88978	0	0
TBP	TATA-box-binc	H0Y6D8	11605	0	0
TBRG4	Protein TBRG4	H7C4R5	29590	0	0
TCEB1	Transcription e	R4GMY8	6963	41	1
TCEB2	Transcription e	I3L0M9	15592	108	3
TCERG1	Transcription e	O14776	123823	0	0
TCOF1	Treacle protein	J3KQ96	144040	0	0
TECR	Very-long-chai	Q9NZ01	36011	0	0
TEX10	Testis-express	Q9NXF1	105608	0	0
TFAM	Transcription f	H7BYN3	25662	0	0
TFIP11	Tuftelin-intera	Q9UBB9	96758	0	0
TGM1	Protein-glutam	H0YLT9	23400	0	0
THOC1	THO complex s	J3KT14	28089	0	0
THOC2	THO complex s	Q8NI27	182659	0	0
THOC6	THO complex s	Q86W42	37511	0	0
THRAP3	Thyroid hormo	Q9Y2W1	108601	0	0
TIA1	Nucleolysin TI/	F8W8I6	42808	0	0
TIAL1	Nucleolysin TI/	Q01085	43421	0	1
TIMM13	Mitochondrial i	Q9Y5L4	10493	0	0
TIMM50	Mitochondrial i	M0R0C3	28204	0	0
TJP1	Tight junction		G3V1L9	197339	0	0
TKT	Transketolase	B4E022	62839	0	0
TMCC3	Transmembran	G3V207	50143	35	1
TMCO1	Transmembran	J3QQY2	11365	0	0
TMED10	Transmembran	P49755	24960	69	2
TMED2	Transmembran	F5GX39	13622	32	1
TMEM109	Transmembran	Q9BVC6	26194	0	0
TMEM167A	Protein kish-A	Q8TBQ9	8054	0	0
TMEM189	HCG2044781 C	G3V2F7	42181	0	0
TMEM194A	Transmembran	G3V5K2	12019	0	0
TMEM201	Transmembran	H0Y4R5	59803	0	0
TMEM33	Transmembran	D6RAA6	25207	0	0
TMEM43	Transmembran	Q9BTV4	44847	0	0
TMEM97	Transmembran	J3KT68	10969	32	1
TMOD3	Tropomodulin-	H0YKU1	20889	0	0
TMPO	Lamina-associ	P42167	75446	35	1
TMPRSS13	Transmembran	J3KQC6	63127	0	1

TMX1	Thioredoxin-re	G3V448	11904	0	0
TNRC6B	Isoform 2 of Tr	Q9UPQ9-1	182703	0	0
TOMM20	Mitochondrial i	Q15388	16288	0	0
TOMM22	Mitochondrial i	Q9NS69	15512	0	0
TOMM40	Mitochondrial i	O96008	37869	0	0
TOMM5	Mitochondrial i	F8W8Z9	10969	0	0
TOP1	DNA topoisom	P11387	90669	0	0
TOP2A	DNA topoisom	P11388	174276	0	0
TOP2B	DNA topoisom	E9PCY5	130398	0	0
TP53	Cellular tumor	P04637	43625	0	0
TPI1	Triosephospha	P60174	30772	83	3
TPM3	Tropomyosin 3	Q5VU58	33202	0	0
TPR	Nucleoprotein	P12270	267131	156	3
TRA2A	Transformer-2	Q13595	32669	46	1
TRA2B	Transformer-2	P62995	33646	113	2
TRAM1	Translocating	G3XAN4	33414	0	0
TRAP1	Heat shock prc	I3L0K7	57184	0	0
TRIM21	E3 ubiquitin-pr	F5H012	54047	0	0
TRIM23	E3 ubiquitin-pr	P36406	64025	38	1
TRIM28	Transcription i	Q13263	88493	0	0
TRIM6-TRIM34	Protein TRIM6-	B2RNG4	97666	0	0
TRMU	Mitochondrial t	O75648	47714	35	1
TRRAP	Transformatior	H0Y4W2	405650	0	0
TSPAN10	Tetraspanin-1(	Q9H1Z9	36475	0	1
TTN	Titin (Fragmen	H7C1P9	109167	0	0
TUBA1A	Tubulin alpha-	Q71U36	50104	608	12
TUBA1C	Tubulin alpha-	F5H5D3	57693	374	6
TUBA4B	Putative tubuli	Q9H853	27534	12	0
TUBB	Tubulin beta cl	P07437	49639	134	3
TUBB2B	Tubulin beta-2	Q9BVA1	49921	0	0
TUBB3	Tubulin beta-3	Q13509	50400	381	6
TUBB4B	Tubulin beta-4	P68371	49799	140	2
TUFM	Elongation fact	P49411	49510	319	7
TUSC3	Tumor suppres	D6RA37	33068	0	0
TXN	Thioredoxin OS	P10599	11730	1	0
TXNL4A	Thioredoxin-lik	K7ESL1	8457	0	0
U2AF1	Splicing factor	Q01081	27854	200	6
U2AF2	Splicing factor	K7ENG2	53467	830	20
U2SURP	U2 snRNP-ass	O15042	118219	2279	60

UBA1	Ubiquitin-like r	Q5JRR6	56816	0	0
UBA52	Ubiquitin-60S r	M0R1V7	12795	36	1
UBB	Ubiquitin (Frag	J3QS39	10463	52	2
UBE2M	NEDD8-conjug	M0QX69	8234	1	0
UBE2NL	Putative ubiqui	Q5JXB2	17366	0	0
UBTF	Nucleolar trans	E9PKP7	87380	0	0
UCHL1	Ubiquitin carbo	D6R956	26823	32	1
UFC1	Ubiquitin-fold r	Q9Y3C8	19446	0	0
UFL1	E3 UFM1-prote	O94874	89540	0	0
UGGT1	UDP-glucose:g	Q9NYU2	177078	0	0
UGT2B15	UDP-glucuronc	P54855	60996	0	0
UQCR10	Cytochrome b-	Q9UDW1	7304	0	0
UQCRC1	Cytochrome b-	P31930	52612	0	0
UQCRC2	Cytochrome b-	H3BP04	19460	0	0
URB1	Nucleolar pre-r	O60287	254227	0	0
USMG5	Up-regulated d	Q96IX5	6453	0	0
USP17L24	Ubiquitin carbo	Q0WX57	59673	0	0
USP28	Ubiquitin carbo	H0YG96	41909	0	0
USP39	U4/U6.U5 tri-s	B9A018	62005	0	0
USP9X	Probable ubiqu	Q93008	292094	0	0
UTP14C	U3 small nucle	Q5TAP6	87135	0	0
UTP15	U3 small nucle	H0Y8P4	61393	0	0
UTP18	U3 small nucle	Q9Y5J1	61964	0	0
UTP20	Small subunit p	O75691	318182	0	0
UTP3	Something abo	Q9NQZ2	54525	0	0
UTS2	Urotensin-2 OS	Q5H8X8	16266	0	0
VAMP2	Vesicle-associa	J3QRU4	12243	0	0
VAPA	Vesicle-associa	Q9P0L0	27875	0	0
VAPB	Vesicle-associa	O95292	27211	63	1
VARS	Valine--tRNA li	H0Y426	32490	0	0
VCP	Transitional en	P55072	89266	131	3
VDAC1	Voltage-depen	P21796	30754	158	3
VDAC2	Voltage-depen	B4DKM5	27462	132	3
VDAC3	Voltage-depen	F5H740	30738	44	1
VIM	Vimentin OS=H	P08670	53619	986	19
WAPAL	Wings apart-lik	Q7Z5K2	132863	0	0
WARS	Tryptophan--tR	P23381	53132	37	1
WBP11	WW domain-bi	F5H5G4	23147	0	0
WDR12	Ribosome biog	Q9GZL7	47678	0	0

WDR18	WD repeat-cor	U3KQC1	43321	43	1
WDR3	WD repeat-cor	Q9UNX4	106032	0	0
WDR33	pre-mRNA 3~ (	Q9C0J8	145799	40	1
WDR36	WD repeat-cor	Q8NI36	105255	0	0
WDR43	WD repeat-cor	Q15061	74843	0	0
WDR5	WD repeat-cor	P61964	36565	0	0
WDR77	Methylosome p	B4DP38	36701	0	0
WDR82	WD repeat-cor	Q6UXN9	35056	0	0
WHSC1L1	Histone-lysine	B7ZL11	160212	0	0
WNK2	Serine/threoni	H0Y7T5	234438	0	0
XAB2	Pre-mRNA-spl	F5H315	99617	0	0
XPNPEP3	Probable Xaa-l	Q9NQH7	56997	4	0
XPO1	Exportin-1 OS=	O14980	123306	0	0
XRCC5	X-ray repair cr	P13010	82652	82	2
XRCC6	X-ray repair cr	P12956	69799	221	4
XRN2	5~-3~ exoribo	B4DZC3	102351	0	0
YBX1	Nuclease-sens	H0Y449	41991	0	0
YEATS2	YEATS domain	H0Y6M6	21152	35	1
YIF1B	Protein YIF1B (	Q5BJH7	34413	0	0
YLPM1	Isoform 4 of YL	P49750-4	241495	0	0
YTHDC1	YTH domain-c	J3QR07	85536	0	0
YWHAB	14-3-3 protein	P31946	28065	112	2
YWHAE	14-3-3 protein	P62258	29155	208	6
YWHAG	14-3-3 protein	P61981	28285	254	6
YWHAH	14-3-3 protein	A2IDB2	28201	0	0
YWHAQ	14-3-3 protein	P27348	27747	56	1
YWHAZ	14-3-3 protein	E7EX29	28019	189	5
ZBBX	Zinc finger B-b	F2Z370	87945	0	0
ZC3H14	Zinc finger CC(	H0YJA2	73271	0	0
ZC3H3	Zinc finger CC(	Q8IXZ2	101878	0	0
ZFYVE19	Zinc finger FY\	H3BN64	29680	46	1
ZMYM2	Zinc finger MY	Q9UBW7	154810	0	0
ZMYM4	Zinc finger MY	Q5VZL5	172677	0	0
ZMYM6NB	Uncharacterize	Q8NCS4	16873	0	0
ZMYND8	Protein kinase	Q2HXV1	126336	0	0
ZNF326	DBIRD comple	Q5BKZ1	65613	361	6
ZNF638	Zinc finger pro	Q14966	220488	0	0
ZNF687	Zinc finger pro	H0Y5I5	75622	0	0
ZRANB2	Zinc finger Rar	O95218	37382	0	0

ZSCAN2	Zinc finger and	F5GY18	22609	0	0

gene name	protein score	group
DDX3X	218	mRNA binding
ELAVL1	182	
ILF2	590	
PABPC1	33	
SRRT	107	
SNRPD1	259	Sm
SNRPD2	161	
SNRPD3	38	
SNRPE	66	
SNRPG	101	
SNRPA	112	U1
PHF5A	78	U2
SF3A1	1062	
SF3A2	565	
SF3A3	1019	
SF3B1	2486	
SF3B14	108	
SF3B2	1667	
SF3B3	1781	
SF3B4	181	
SF3B5	127	
SNRPA1	307	
SNRPB2	307	
CHERP	1107	U2 rel
DDX42	35	
DDX46	38	
DHX15	1481	
DNAJC8	666	
RBM17	739	
SMNDC1	106	
U2AF1	200	
U2AF2	783	
U2SURP	1903	
BUB3	118	
C19orf43	197	
CCAR1	1670	

DDX17	225	A
DDX5	267	
FUS	213	
RBM10	990	
RBM39	92	
RBM5	1226	
SF1	655	
SUGP1	914	
EFTUD2	170	U5
PRPF6	48	
PRPF8	1858	
SNRNP200	1276	
SNRNP40	61	
NHP2L1	57	U4/U6
PRPF3	30	
HSPA8	817	Prp19
PRPF19	171	
AQR	47	Prp19 rel
GNB2L1	84	C2
MATR3	588	
LSM6	62	LSm
SRSF1	4	SR
SRSF10	5	
SRSF2	40	
SRSF9	351	
TRA2A	46	
TRA2B	113	
HNRNPA0	99	hnRNP
HNRNPA1	129	
HNRNPA2B1	132	
HNRNPC	265	
HNRNPD	194	
HNRNPH1	45	
HNRNPH3	102	
HNRNPK	132	
HNRNPM	386	
HNRNPR	553	
HNRNPU	224	

HNRNPUL1	256	
PCBP1	30	
PTBP1	183	
RALY	125	
RBMX	214	
SYNCRIP	387	
C16orf80	199	MISC
CIRBP	6	
ILF3	749	
KHDRBS1	402	
PRMT5	85	
ACIN1	46	EJC/TREX
DDX39B	72	
EIF4A3	197	
MAGOH	46	
RBM8A	76	
SAP18	48	
2	124	
4	34	
7	133	
8	39	
ACAT1	220	
ACTL6A	17	
ACTN4	133	
ADAR	486	
AGO1	77	
AHNAK	79	
AK2	201	
AKAP8	175	
ALDOA	343	
ALDOC	172	
ANKFY1	55	
ANP32A	219	
ANP32B	205	
ANXA2	60	
ANXA6	47	
API5	69	
APRT	22	

ARF4	148
ARF6	38
ATP13A1	35
ATP5A1	149
ATP5D	114
ATP5G1	34
ATP5L	40
ATPIF1	38
BCAP31	157
BLOC1S6	40
BOLA2	53
C14orf166	330
C19orf10	64
C1QBP	116
C21orf33	54
CALM2	50
CALR	252
CAND1	38
CBX1	41
CCT4	163
CCT5	58
CCT8	31
CFL1	33
CFTR	35
CHD4	111
CHN1	30
CKAP5	123
CKB	103
CKMT1A	71
CLTC	653
CNPY2	52
CNTNAP4	11
COMT	47
COPB2	47
COPG2	48
COX7A2	59
CPA5	30
CPSF1	76

CPSF6	223
CPSF7	150
CPVL	88
CRTAP	178
CS	73
CSNK2B-LY6G5B-1181	68
CTBP2	32
CTTNBP2	2
CYCS	50
DAD1	91
DAPP1	1
DAZAP1	84
DCXR	56
DDB1	119
DDX1	70
DDX39A	62
DHCR7	35
DIABLO	66
DLD	200
DNAI2	32
DNAJA2	36
DNAJA3	42
DNAJC10	35
DNMT1	34
DYNC1H1	774
ECHS1	79
ECI1	54
EDC4	32
EDF1	48
EEF1A1P5	28
EEF1A2	429
EEF1G	232
EEF2	22
EIF4A2	33
EIF4H	82
EIF5A	203
ENDOV	30
ENO1	193

EP400	32
ERP29	75
ETFA	92
ETFB	82
EWSR1	4
FARP2	33
FASN	40
FBL	34
FLNA	364
FUBP1	143
GANAB	200
GAPDH	204
GCN1L1	256
GIGYF2	42
GK	39
GNA12	42
GNAS	42
GNB1	40
GOT2	116
GPI	131
GSTO1	38
GTF2I	43
H2AFZ	99
H3F3A	8
HDAC4	33
HIST1H2AG	228
HIST1H2BN	142
HMGB1P1	34
HNRNPDL	57
HNRNPL	58
HNRNPUL2	636
HSD17B10	49
HSD17B12	162
HSD17B4	220
HSP90AA1	292
HSP90B1	197
HSPA4	56
HSPA5	1097

HSPA6	100
HSPA9	514
HSPD1	303
HSPE1	89
IGLL5	1
IREB2	74
KATNAL2	51
KBTBD3	1
KHSRP	58
KLRC4-KLRK1	34
LAMA5	33
LANCL1	50
LCN1	134
LDHA	107
LDHB	131
LEPRE1	275
LIG3	32
LIMA1	14
LMNA	149
LMNB1	461
LMNB2	255
LUC7L2	77
MAP1B	68
MDH2	175
MDN1	171
METTL3	35
MSH2	35
MT-CO2	37
MYEF2	42
MYL12A	36
NCL	936
NDUFS3	93
NKRF	123
NME1-NME2	142
NME3	30
NONO	111
NPM1	210
NUDT16L1	91

ungrouped

NUDT21	563
NUP210	71
P4HB	266
PA2G4	93
PABPN1	38
PARP1	73
PCMT1	28
PCNP	433
PDIA3	341
PDIA4	32
PELP1	31
PFKP	34
PFN1	37
PGAM1	38
PGK1	133
PHB	189
PHB2	272
PHGDH	136
PLOD1	246
POLR2A	125
PPIA	157
PPIB	72
PPIF	163
PPP2CB	32
PPP6R2	32
PRAF2	56
PRC1	5
PRDX2	13
PRDX3	1
PRDX4	163
PRDX6	48
PRKCSH	77
PRKDC	753
PRPF39	15
PSMA1	31
PSMA3	82
PSMB5	115
PTMA	52

RAB10	142
RAB15	91
RAB33B	31
RAB35	91
RAB5C	126
RAB6A	31
RAD50	85
RAN	94
RAP1B	34
RBBP4	121
RBM14	405
RBM15	52
RBM6	330
RBMXL1	237
RBP3	30
RCC1	31
RCC2	40
RECQL	40
RGPD1	57
RHCE	34
RP1	33
RPL12	79
RPN1	36
RPS5	88
RPSAP58	48
RTCB	329
RTN1	38
RTN3	78
RUFY2	4
RUVBL1	99
S100A8	79
SAFB	660
SCAMP1	77
SEC22B	89
SET	142
SFRS3	97
SFSWAP	40
SLC25A10	60

SLC25A11	48
SLC25A3	39
SLC25A5	257
SLIRP	33
SMARCA4	47
SMARCC2	86
SMC1A	33
SMC4	220
SNRPN	214
SOD1	94
SPIN2B	32
SPRR3	38
SPTAN1	116
SPTBN1	72
STMN1	134
STMN2	94
TAF15	81
TARDBP	39
TBCA	53
TCEB1	41
TCEB2	108
TMCC3	35
TMED10	69
TMED2	32
TMEM97	32
TMPO	35
TPI1	83
TPR	156
TRIM23	38
TRMU	35
TUBA1A	608
TUBA1C	374
TUBA4B	12
TUBB	134
TUBB3	381
TUBB4B	140
TUFM	319
TXN	1

UBB	52	
UBE2M	1	
UCHL1	32	
VAPB	63	
VCP	131	
VDAC1	158	
VDAC2	132	
VDAC3	44	
VIM	885	
WARS	37	
WDR18	43	
WDR33	40	
XPNPEP3	4	
XRCC5	82	
XRCC6	221	
YEATS2	35	
YWHAB	112	
YWHAE	137	
YWHAG	254	
YWHAQ	56	
YWHAZ	189	
ZFYVE19	46	
ZNF326	361	

protein score

high

2500
2000
1500
1000
500
0

low

GeneName	prot_desc	prot_acc	prot_mass	prot_.score	prot_.matches
1	Uncharacterize	M0QYC6	12045	0	0
2	Uncharacterize	F5H423	23331	0	0
3	Uncharacterize	H0YHG0	59149	0	0
4	Uncharacterize	H7BZT4	10832	0	0
5	Ig kappa chain	P01614	12668	0	3
6	Uncharacterize	H7C0C1	21328	0	0
A2M	Alpha-2-macro	P01023	163188	3	0
AARD	Alanine and ar	Q4LEZ3	17564	39	1
AATF	Protein AATF (	Q9NY61	63094	0	0
ABCB6	ATP-binding ca	H7BXK9	77498	0	0
ACAA2	3-ketoacyl-Co/	K7EME0	36260	0	0
ACAT1	Acetyl-CoA ace	P24752	45171	0	0
ACIN1	Apoptotic chro	S4R3H4	145355	33	1
ACOT13	Acyl-coenzyme	Q9NPJ3	14951	0	0
ACP1	Low molecular	F2Z2Q9	7496	0	0
ACSL1	Long-chain-fat	H0Y9U7	32788	32	1
ACTA1	Actin, alpha sk	Q5T8M7	37800	0	0
ACTB	Actin, cytoplas	P60709	41710	56	2
ACTBL2	Beta-actin-like	Q562R1	41976	0	0
ACTC1	Actin, alpha ca	P68032	41992	67	3
ACTG1	Actin, cytoplas	I3L1U9	23830	0	0
ACTL6A	Actin-like prote	O96019	47430	1	0
ACTN1	Alpha-actinin-1	G3V2W4	102993	0	0
ACTN4	Alpha-actinin-4	O43707	104788	0	0
ADAD2	Adenosine dea	H3BNP6	28653	0	0
ADAR	Double-strande	H0YCK3	140739	577	12
ADC	Arginine decarl	Q96A70	49947	0	0
ADNP	Activity-depen	Q9H2P0	123485	0	0
AGL	Glycogen debra	P35573	174652	0	0
AGO1	Protein argona	Q9UL18	97152	0	0
AGO2	Protein argona	Q9UKV8	97146	0	0
AGO3	Protein argona	Q9H9G7	97298	86	2
AHCTF1	Protein ELYS (	Q8WYP5	252342	0	0
AHCY	Adenosylhomo	P23526	47685	0	0
AHNAK	Neuroblast diff	Q09666	628699	0	0
AIFM1	Apoptosis-indu	O95831	66859	0	0
AK2	Adenylate kina	F8W1A4	25614	0	0
AKAP8	A-kinase anch	O43823	76061	204	6

AKAP8L	A-kinase anch	Q9ULX6	71604	144	4
AKR1A1	Alcohol dehydr	Q5T621	16506	0	0
AKR1B1	Aldose reducta	E9PCX2	29426	0	0
ALDH18A1	Delta-1-pyrroli	P54886	87248	0	0
ALDOA	Fructose-bisph	H3BPS8	39315	0	0
ALDOC	Fructose-bisph	P09972	39431	0	0
ALG13	Isoform 2 of Pu	Q9NP73-2	18213	32	1
ALYREF	THO complex s	E9PB61	27541	0	0
ANKFY1	Ankyrin repeat	Q9P2R3	128318	0	0
ANP32A	Acidic leucine-	H0YN26	19985	1	0
ANP32B	Acidic leucine-	Q5T6W8	28770	32	1
ANP32C	Acidic leucine-	O43423	26746	98	2
ANXA2	Annexin (Fragr	H0YKL9	19518	0	0
ANXA5	Annexin OS=H	D6RBL5	29319	0	0
ANXA6	Annexin (Fragr	H0YC77	10129	0	0
APEX1	DNA-(apurinic	G3V3M6	29172	0	0
APH1B	Gamma-secret	H0YKZ9	20749	0	0
APOB	Apolipoprotein	P04114	515283	0	0
APOBEC3C	DNA dC->dU-e	Q9NRW3	22811	90	2
APRT	Adenine phosp	H3BQB1	17551	0	0
ARF3	ADP-ribosylati	B7ZB63	20588	0	0
ARF4	ADP-ribosylati	P18085	20498	0	0
ARF5	ADP-ribosylati	P84085	20517	0	0
ARF6	ADP-ribosylati	P62330	20069	69	1
ARHGEF17	Rho guanine nu	Q96PE2	221535	33	1
ARHGEF18	Rho guanine nu	M0QZS0	91352	0	0
ARHGEF19	Rho guanine nu	H0Y4D8	34549	0	0
ARL1	ADP-ribosylati	B4DWW1	18554	70	1
ARL8B	ADP-ribosylati	B4DI85	18630	0	0
ARPC3	Actin-related p	F8VR50	9720	0	0
ARPC4-TTLL3	Protein ARPC4	H7C0A3	21045	0	0
ASAP2	Arf-GAP with S	O43150	111581	4	0
ATAD3B	ATPase family	Q5T9A4	72527	0	0
ATIC	Phosphoribosy	F5GWY2	58579	0	0
ATP13A1	Probable catio	Q9HD20	132870	0	0
ATP1A1	Sodium/potass	P05023	112824	0	0
ATP5A1	ATP synthase s	K7ENP3	59714	0	0
ATP5B	ATP synthase s	F8VPV9	56525	0	0
ATP5C1	ATP synthase s	B4DL14	27495	0	0

ATP5D	ATP synthase s	P30049	17479	0	0
ATP5G1	ATP synthase l	D6R9H7	10188	0	0
ATP5J2-PTCD3	Pentatricopept	G3V325	84057	0	0
ATP5L	ATP synthase s	E9PN17	8447	0	0
ATP5O	ATP synthase s	H7C086	8128	0	0
ATP6V0C	V-type proton /	P27449	15725	0	0
ATP6V1C1	V-type proton /	E7EV59	35686	31	1
ATPIF1	ATPase inhibit	Q9UII2	12241	0	0
BAG2	BAG family mo	B4DXE2	20176	0	0
BAG4	BAG family mo	O95429	49563	43	1
BANF1	Barrier-to-auto	O75531	10052	0	0
BARD1	BRCA1-associa	E7EUI3	62141	0	0
BAX	Apoptosis regu	Q07812	21171	0	0
BAZ1B	Tyrosine-prote	Q9UIG0	170796	0	0
BCAP31	B-cell receptor	P51572	27974	0	0
BCAS2	Pre-mRNA-spl	O75934	26115	0	0
BCLAF1	Bcl-2-associat	E9PK09	83183	0	0
BEAN1	Isoform 3 of Pr	Q3B7T3-3	22331	0	0
bK150C2.9	BK150C2.9 pro	Q6ICH2	24136	0	0
BLOC1S6	Biogenesis of l	H3BNE3	13587	68	2
BMS1	Ribosome biog	Q14692	145716	0	0
BOLA2	BolA-like prote	H3BTW0	10110	0	0
BPTF	Nucleosome-re	F5GXF5	271364	0	0
BRI3BP	BRI3-binding p	Q8WY22	27818	0	0
BRIX1	Ribosome biog	Q8TDN6	41375	0	0
BSG	Basigin OS=Hc	P35613	42174	0	0
BUB3	Mitotic checkp	J3QT28	31684	0	0
BUD31	Protein BUD31	C9JNV2	13559	0	0
BYSL	Bystin OS=Hor	Q13895	49570	0	0
C10orf115	Putative uncha	Q5QP74	10337	0	0
C11orf31	Selenoprotein I	H0YE28	10435	0	0
C11orf84	Uncharacterize	Q9BUA3	41011	0	0
C12orf23	UPF0444 trans	Q8WUH6	11741	47	1
C14orf166	UPF0568 prote	Q9Y224	28051	373	6
C16orf80	UPF0468 prote	Q9Y6A4	22760	207	5
C19orf10	UPF0556 prote	Q969H8	18783	0	0
C19orf43	Uncharacterize	Q9BQ61	18408	563	22
C1orf167	Uncharacterize	H0Y5F2	68415	0	0
C1QBP	Complement c	I3L3B0	31343	82	1

C21orf33	ES1 protein ho	H7C1F6	27429	0	0
C2CD4B	C2 calcium-de|	A6NLJ0	38745	0	0
C4A	Complement C	P0C0L4	192664	0	0
C7orf50	Uncharacterize	C9JQV0	21870	0	0
CACNG6	Voltage-depen	Q9BXT2	28111	0	0
CACYBP	Calcyclin-bindi	Q9HB71	26194	0	0
CALCOCO1	Calcium-bindir	Q9P1Z2	77289	30	1
CALM2	Calmodulin (Fr	H0Y7A7	20749	0	0
CALR	Calreticulin (Fr	K7EJB9	48112	0	0
CALU	Calumenin OS=	O43852	37084	31	1
CAND1	Cullin-associat	Q86VP6	136289	0	0
CANX	Calnexin OS=H	B4DGP8	71458	0	0
CAPZA2	F-actin-cappin	P47755	32929	0	0
CAPZB	Capping protei	B1AK87	29277	0	0
CARHSP1	Calcium-regula	Q9Y2V2	15882	0	0
CASP14	Caspase-14 OS	P31944	27662	0	0
CBR1	Carbonyl reduc	B4DFK7	18750	0	0
CBX1	Chromobox prc	J3KS05	20115	0	0
CBX3	Chromobox prc	Q13185	20798	0	0
CBX5	Chromobox prc	P45973	22211	0	0
CCAR1	Cell division cy	F5H1H2	132739	1961	56
CCAR2	Cell cycle and a	H0YB24	68881	0	0
CCDC58	Coiled-coil don	Q4VC31	16609	0	0
CCDC86	Coiled-coil don	B4DY99	12735	0	0
CCNK	Cyclin-K OS=H	G3V5E1	43377	31	1
CCR9	C-C chemokine	P51686	41987	0	0
CCT2	T-complex prot	F5GWF6	57452	0	0
CCT4	T-complex prot	P50991	57888	0	0
CCT5	T-complex prot	B4DYD8	49495	0	0
CCT6A	T-complex prot	B4DPJ8	54833	0	0
CCT7	T-complex prot	F5GZK5	59329	0	0
CCT8	T-complex prot	B4DEM7	57608	0	0
CD2BP2	CD2 antigen cy	O95400	37623	49	1
CDC5L	Cell division cy	Q99459	92194	0	0
CDK1	Cyclin-depende	E5RIU6	21724	0	0
CEBPZ	CCAAT/enhanc	Q03701	120898	0	0
CENPB	Major centrom	P07199	65132	0	0
CENPV	Isoform 3 of Ce	Q7Z7K6-3	29927	0	0
CEP85	Centrosomal p	Q6P2H3	85586	31	2

CEP85L	Centrosomal p	Q5SZL2	91751	0	0
CFL1	Cofilin 1 (Non-	G3V1A4	16801	0	0
CGGBP1	CGG triplet rep	Q9UFW8	18809	0	0
CHCHD3	Coiled-coil-hel	G3V1K1	15549	0	0
CHD4	Chromodomain	F5GWX5	216967	308	8
CHD8	Chromodomain	H0YJG4	101664	0	0
CHERP	Calcium homeo	J3QK89	104868	1622	187
CHN1	N-chimaerin O	B8ZZ96	31322	0	0
CHTOP	Chromatin targ	Q9Y3Y2	26380	0	0
CIRBP	Cold-inducible	B4E2X2	28342	20	0
CIRH1A	Cirhin (Fragme	H3BSH7	77894	0	0
CISD2	CDGSH iron-su	I3L1N9	5141	0	0
CKAP4	Cytoskeleton-a	Q07065	65983	0	0
CKAP5	Cytoskeleton-a	Q14008	225352	14	0
CKB	Creatine kinase	H0YJG0	42617	0	0
CLASP2	CLIP-associati	B3KR06	49127	0	0
CLTC	Clathrin heavy	Q00610	191493	0	0
CMAS	N-acylneurami	F5H296	9190	0	0
CNIH4	Protein cornich	A6NLH6	15760	0	0
CNPY2	Protein canopy	F8VXJ7	19040	0	0
CNTNAP4	Contactin-asso	F5H107	139833	0	0
COIL	Coilin OS=Hom	P38432	62570	0	0
COLGALT1	Procollagen ga	M0QX72	71590	0	0
COMT	Catechol O-me	E7EMS6	24822	0	0
COPA	Coatomer subu	P53621	138258	0	0
COPB1	Coatomer subu	E9PP73	55502	0	0
COPB2	Coatomer prote	B4DZI8	98984	0	0
COPE	Coatomer prote	M0QXB4	36901	0	0
COPZ1	Coatomer subu	F8VS17	18363	0	0
CORO1C	Coronin OS=H	B4E3S0	41577	0	0
COX17	Cytochrome c	C9J8T6	10846	0	0
COX4I1	Cytochrome c	P13073	19564	0	0
COX5A	Cytochrome c	H3BNX8	17224	0	0
COX5B	Cytochrome c	P10606	13687	0	0
COX6B1	Cytochrome c	K7EQD3	4781	0	0
COX7A2	Cytochrome c	H0UI06	12836	0	0
CPA5	Carboxypeptida	Q8WXQ8	49005	30	1
CPSF1	Cleavage and p	Q10570	160782	264	6
CPSF2	Cleavage and p	Q9P2I0	88431	186	4

CPSF3	Cleavage and p	G5E9W3	73430	76	2
CPSF4	Cleavage and p	B7Z7B0	21942	0	0
CPSF6	Cleavage and p	F8WJN3	52238	219	5
CPSF7	Cleavage and p	F5H669	41241	304	6
CPVL	Probable serin	Q9H3G5	54129	292	7
CRBN	Protein cereblo	J3QT87	45424	59	1
CRNKL1	Crooked neck-	Q5JY65	99111	79	1
CROCC	Rootletin (Frag	B1AKD8	149087	0	0
CRTAP	Cartilage-asso	O75718	46532	313	8
CS	Citrate synthas	B4DJV2	50400	0	0
CSE1L	Exportin-2 OS=	B4DUC5	110346	0	0
CSNK2A1	Casein kinase I	E7EU96	45282	0	0
CSNK2B-LY6G	Chimera CSNK	Q5SRQ3	26656	0	0
CSTA	Cystatin-A OS=	C9J0E4	7082	0	0
CSTB	Cystatin-B OS=	P04080	11133	0	0
CSTF2	Cleavage stimu	E7EWR4	62902	0	0
CTNND1	Catenin delta-1	C9JZR2	104784	0	0
CTSD	Cathepsin D lig	H7C1V0	20359	0	0
CTTN	Src substrate c	H7C314	25079	0	0
CTTNBP2	Cortactin-bindi	H0Y448	125549	1	1
CYB5B	Cytochrome b5	H3BUX2	15707	0	0
CYB5R3	NADH-cytochro	B1AHF3	16686	0	0
CYCS	Cytochrome c (	C9JFR7	11326	0	0
DAD1	Dolichyl-diphos	F5GXX5	9548	0	0
DAPP1	Dual adapter fo	J3KNB3	30156	11	1
DARS	Aspartate--tRN	C9J7S3	19957	0	0
DAZAP1	DAZ-associate	K7EQ02	24789	0	0
DBI	Acyl-CoA-bindi	P07108	10038	0	0
DBN1	Drebrin (Fragm	D6RFI1	13428	0	0
DCAF13	DDB1- and CU	Q9NV06	51369	0	0
DCAKD	Dephospho-Co	K7ESP4	24162	0	0
DCD	Dermcidin OS=	P81605	11277	0	0
DCUN1D5	DCN1-like prot	H0YCN4	22918	0	0
DCXR	L-xylulose redu	J3KRZ4	23945	0	0
DDB1	DNA damage-b	Q16531	126887	132	2
DDOST	Dolichyl-diphos	U3KQ84	46548	0	0
DDT	D-dopachrome	J3KQ18	14184	0	0
DDX1	ATP-dependen	Q92499	82380	170	4
DDX17	Probable ATP-	H3BLZ8	80389	192	5

DDX18	ATP-dependen	Q9NVP1	75359	0	0
DDX21	Nucleolar RNA	Q9NR30	87290	46	1
DDX23	Probable ATP-	Q9BUQ8	95524	0	0
DDX24	ATP-dependen	G3V529	91424	0	0
DDX27	Probable ATP-	Q96GQ7	89779	0	0
DDX31	Probable ATP-	F8WAJ0	80895	0	0
DDX39	ATP-dependen	B1Q2N1	30469	11	0
DDX39A	ATP-dependen	O00148	49098	0	0
DDX39B	Spliceosome R	B4DIJ6	48794	0	0
DDX3X	ATP-dependen	O00571	73198	0	0
DDX3Y	Uncharacterize	B4DXX7	72886	140	3
DDX46	Probable ATP-	Q7L014	117290	31	1
DDX5	Probable ATP-	J3KTA4	69044	0	0
DDX50	ATP-dependen	Q9BQ39	82514	0	0
DDX54	ATP-dependen	Q8TDD1	98534	0	0
DDX55	ATP-dependen	F5H5U2	65149	0	0
DENR	Density-regula	F8VVL1	17759	0	0
DHCR7	7-dehydrochol	E9PIP9	12078	0	0
DHX15	Putative pre-m	O43143	90875	1456	223
DHX30	Putative ATP-c	H7BXY3	130469	55	1
DHX36	Probable ATP-	F5GZS0	113016	0	0
DHX9	ATP-dependen	Q08211	140869	0	0
DIABLO	Diablo homolo	F5GXT8	15243	0	0
DIDO1	Death-inducer	Q9BTC0	243723	0	0
DKC1	H/ACA ribonuc	O60832	57638	0	0
DKFZp781K13	NADH dehydro	Q5H9R2	13554	0	0
DLD	Dihydrolipoyl d	B4DHG0	43560	0	0
DNAJA1	DnaJ homolog	P31689	44839	0	0
DNAJA3	DnaJ homolog	E7ES32	33061	44	1
DNAJC10	DnaJ homolog	Q8IXB1	91021	0	2
DNAJC2	DnaJ homolog	Q99543	71952	0	0
DNAJC8	DnaJ homolog	O75937	29823	704	30
DNAJC9	DnaJ homolog	Q8WXX5	29891	0	0
DNTTIP2	Deoxynucleotic	J3KP30	66135	0	0
DOLPP1	Dolichyldiphos	Q86YN1	27013	0	0
DPM3	Dolichol-phosp	Q9P2X0	10087	0	0
DPY30	Protein dpy-30	Q9C005	11243	0	0
DPYSL5	Dihydropyrimid	E7EWB4	20853	0	0
DRAP1	Dr1-associatec	C9JCC6	23190	0	0

DSG1	Desmoglein-1 (	Q02413	113676	43	1
DSP	Desmoplakin C	P15924	331569	0	0
DUT	Deoxyuridine 5	H0YKC5	23724	0	0
DYNC1H1	Cytoplasmic dy	Q14204	532072	1706	36
DYNLL2	Dynein light ch	Q96FJ2	10343	37	1
DYNLRB1	Dynein light ch	B1AKR6	16242	0	0
EBNA1BP2	EBNA1 binding	H7C2Q8	40659	0	0
EBP	3-beta-hydroxy	Q15125	26336	0	0
ECH1	Delta(3,5)-Delt	Q13011	35793	0	0
ECHS1	Enoyl-CoA hyd	P30084	31367	0	0
ECI1	Enoyl-CoA delt	H3BS70	32795	0	0
EDF1	Endothelial diff	O60869	16359	0	0
EEF1A1	Elongation fact	Q5JR01	15926	44	1
EEF1A1P5	Putative elonga	Q5VTE0	50153	0	0
EEF1A2	Elongation fact	Q05639	50438	0	0
EEF1B2	Elongation fact	P24534	24748	0	0
EEF1D	Elongation fact	H0YCK7	22934	0	0
EEF1E1	Eukaryotic tran	H0YAL7	15330	0	0
EEF1G	Elongation fact	B4DTG2	56114	0	0
EEF2	Elongation fact	P13639	95277	0	0
EFCAB4B	EF-hand calciu	H0YFI5	13969	0	0
EFTUD2	116 kDa U5 sm	K7EJ81	108215	145	2
EIF1AY	Eukaryotic tran	A6NJH9	14446	0	0
EIF3A	Eukaryotic tran	F5H335	162537	0	0
EIF3K	Eukaryotic tran	B7ZAM9	24468	0	0
EIF4A1	Eukaryotic initi	P60842	46125	0	0
EIF4A3	Eukaryotic initi	P38919	46841	0	0
EIF4B	Eukaryotic tran	B4DS13	69657	0	0
EIF4E	Eukaryotic tran	H0Y8J7	18455	0	0
EIF4G1	Eukaryotic tran	E7EUU4	171535	0	0
EIF4H	Eukaryotic tran	Q15056	27368	0	0
EIF5A	Eukaryotic tran	I3L397	16108	0	0
EIF6	Eukaryotic tran	P56537	26582	0	0
ELAVL1	ELAV-like prot	B4DVB8	38972	0	0
EMD	Emerin OS=Ho	P50402	28976	0	0
EMG1	Ribosomal RN/	V9GYP5	26581	0	0
ENO1	Alpha-enolase	P06733	47139	0	0
ENO2	Enolase OS=H	F5H0C8	34741	0	0
ENY2	Transcription a	Q9NPA8	11521	101	2

EP400	E1A-binding pr	Q96L91	343276	36	1
EPB41L2	Band 4.1-like p	Q6ZSX4	22715	0	0
ERH	Enhancer of ru	P84090	12251	12	0
ERLIN2	Erlin-2 (Fragm	E5RHW4	37701	0	0
ERP29	Endoplasmic re	P30040	28975	0	0
ETFA	Electron transf	H0YK49	30202	0	0
ETFB	Electron transf	P38117	27826	0	0
EWSR1	RNA-binding p	B0QYK0	64889	0	0
EXOSC10	Exosome comp	Q01780	100768	0	0
EXOSC4	Exosome comp	E9PI41	28395	0	0
EXOSC5	Exosome comp	M0R050	21243	0	0
EXOSC6	Exosome comp	Q5RKV6	28218	0	0
EZR	Ezrin OS=Hom	E7EQR4	69370	0	0
FABP5	Fatty acid-bind	Q01469	15155	0	0
FAM103A1	RNMT-activati	Q9BTL3	14373	0	0
FAM124B	Protein FAM12	Q9H5Z6	50929	33	1
FAM136A	Protein FAM13	E7EQY1	26758	0	0
FAM160A1	Protein FAM16	Q05DH4	116547	0	0
FAM184B	Protein FAM18	Q9ULE4	120969	0	0
FAM188B	Protein FAM18	Q4G0A6	84319	40	1
FAM213A	Redox-regulato	Q9BRX8	25747	0	0
FAM98A	Protein FAM98	B4DT23	34066	39	1
FARP2	FERM, RhoGEF	F5GZ84	72217	37	1
FASN	Fatty acid synt	P49327	273254	0	0
FAU	40S ribosomal	E9PR30	10898	0	0
FBL	rRNA 2~-O-me	P22087	33763	164	3
FBLL1	rRNA/tRNA 2~	R4GMW7	34782	86	2
FCF1	Chromosome 1	G3V1S4	21933	0	0
FEN1	Flap endonucle	I3L3E9	17899	0	0
FHL1	Four and a half	Q5JXH7	23310	0	0
FICD	Adenosine mor	J3KP49	19252	0	0
FIP1L1	Pre-mRNA 3~-	Q6UN15	66487	63	1
FKBP10	Peptidyl-prolyl	K7ELI6	18858	0	0
FKBP12-Exip2	Peptidyl-prolyl	Q1JUQ5	10112	0	0
FKBP3	Peptidyl-prolyl	Q00688	25161	0	0
FKBP8	Peptidyl-prolyl	J3KQ73	47114	0	0
FLG	Filaggrin OS=H	P20930	434922	9	0
FLG2	Filaggrin-2 OS=	Q5D862	247928	0	0
FLII	Protein flightle	Q13045	144659	0	0

FLNA	Filamin-A OS=	Q5HY54	276378	0	0
FMR1	Fragile X menta	G3V0J0	66745	0	0
FNBP4	Formin-binding	Q8N3X1	110198	30	1
FTH1	Ferritin OS=Ho	G3V192	17643	0	0
FTSJ3	pre-rRNA proce	Q8IY81	96499	0	0
FUBP1	Far upstream e	B4DT31	69821	0	0
FUBP3	Far upstream e	Q96I24	61602	0	0
FUS	RNA-binding p	H3BPE7	53465	32	26
FXR2	Fragile X menta	P51116	74178	0	0
GANAB	Neutral alpha-g	F5H6X6	106807	0	0
GAPDH	Glyceraldehyde	E7EUT5	36030	0	0
GAR1	H/ACA ribonuc	Q9NY12	22334	0	0
GATAD2B	Transcriptional	Q8WXI9	65220	0	0
GCN1L1	Translational a	Q92616	292572	141	3
GDI2	Rab GDP disso	Q5SX86	17771	0	0
GEMIN2	Gem-associate	H0YDP6	22112	0	0
GIPC1	PDZ domain-co	O14908	36027	0	0
GIPC2	PDZ domain-co	Q8TF65	34333	19	0
GJA8	Gap junction al	P48165	48199	30	1
GLO1	Lactoylglutathi	Q04760	20764	0	0
GLRX5	Glutaredoxin-re	Q86SX6	16618	0	0
GMPS	GMP synthase	F8W720	65887	0	0
GNA12	Guanine nucleo	Q03113	44251	0	0
GNAS	Guanine nucleo	Q5JWF2	110956	0	0
GNB1	Guanine nucleo	B1AKQ8	12277	0	0
GNB2L1	Guanine nucleo	H0Y8W2	30244	0	0
GNG12	Guanine nucleo	Q9UBI6	8001	0	0
GNG5	Guanine nucleo	P63218	7314	0	0
GNL2	Nucleolar GTP-	Q13823	83603	0	0
GNL3	Guanine nucleo	Q9BVP2	61954	0	0
GNL3L	Guanine nucleo	Q9NVN8	65532	0	0
GOT2	Aspartate amin	E7ERW2	43002	0	0
GPI	Glucose-6-pho	K7EQ48	53368	0	0
GRB2	Growth factor r	P62993	25190	71	2
GRM1	Metabotropic g	F8W805	101407	0	0
GRN	Paragranulin (F	K7EKL3	41321	0	0
GRPEL1	GrpE protein ho	Q9HAV7	24264	0	0
GSTO1	Glutathione S-t	P78417	27548	0	0
GSTP1	Glutathione S-t	A8MX94	19468	0	0

GTF2I	General transc	P78347	112346	12	1
GTF3C1	General transc	Q12789	238725	0	0
GTPBP4	Nucleolar GTP-	Q9BZE4	73918	0	0
H1F0	Histone H1.0 C	P07305	20850	0	0
H1FX	Histone H1x OS	Q92522	22474	0	0
H2AFX	Histone H2AX	P16104	15135	0	0
H2AFY	Core histone m	O75367	39592	0	0
H2AFY2	Core histone m	Q9P0M6	40033	0	0
H2AFZ	Histone H2A.Z	P0C0S5	13545	0	0
H3F3A	Histone H3 OS	B4DEB1	14044	102	3
H3F3B	Histone H3 (Fr	K7EK07	14905	0	0
HBA2	HCG1745306, i	G3V1N2	11940	0	0
HBD	Hemoglobin su	E9PFT6	15410	0	0
HBE1	Hemoglobin su	A8MUF7	9464	0	0
HCFC1	HCF N-termina	A6NEM2	213343	0	0
HDAC2	Histone deacet	B3KRS5	51965	48	1
HDAC4	Histone deacet	F5GX36	72986	0	0
HEATR1	HEAT repeat-c	Q9H583	242215	0	0
HES6	Transcription c	H7C020	17010	0	0
HINT1	Histidine triad	P49773	13793	0	0
HINT2	Histidine triad	Q9BX68	17151	0	0
HIST1H1A	Histone H1.1 C	Q02539	21829	0	0
HIST1H1C	Histone H1.2 C	P16403	21352	0	0
HIST1H1D	Histone H1.3 C	P16402	22336	0	0
HIST1H1E	Histone H1.4 C	P10412	21852	0	0
HIST1H2AA	Histone H2A ty	Q96QV6	14225	0	0
HIST1H2AC	Histone H2A ty	Q93077	14097	0	0
HIST1H2AD	Histone H2A ty	P20671	14099	0	0
HIST1H2AG	Histone H2A ty	P0C0S8	14083	67	3
HIST1H2BJ	Histone H2B ty	P06899	13896	0	0
HIST1H2BL	Histone H2B ty	Q99880	13944	0	0
HIST1H2BN	Histone H2B O	U3KQK0	18792	86	2
HIST1H4A	Histone H4 OS	P62805	11360	0	1
HIST2H2AA3	Histone H2A ty	Q6FI13	14087	0	0
HIST2H2AB	Histone H2A ty	Q8IUE6	13987	0	0
HIST2H3PS2	Histone H3 OS	Q5TEC6	15421	0	0
HIST3H2A	Histone H2A ty	Q7L7L0	14113	0	0
HIST3H2BB	Histone H2B ty	Q8N257	13900	0	0
HMG20A	High mobility g	Q9NP66	40119	0	0

HMGA1	High mobility g	H7BYM6	34274	0	0
HMGB1	High mobility g	Q5T7C4	24878	0	0
HMGB2	High mobility g	D6R9A6	24019	0	0
HNRNPA0	Heterogeneous	Q13151	30822	127	13
HNRNPA1	Heterogeneous	F8W6I7	38723	0	17
HNRNPA2B1	Heterogeneous	P22626	37407	403	151
HNRNPA3	Heterogeneous	P51991	39571	0	0
HNRNPAB	Heterogeneous	D6R9P3	30284	78	12
HNRNPC	Heterogeneous	G3V4C1	33550	373	8
HNRNPD	Heterogeneous	Q14103	38410	87	13
HNRNPDL	Heterogeneous	O14979	46409	27	0
HNRNPF	Heterogeneous	P52597	45643	54	3
HNRNPH1	Heterogeneous	H0YB39	51197	79	18
HNRNPH2	Heterogeneous	P55795	49232	0	0
HNRNPH3	Isoform 2 of He	P31942-2	36903	232	75
HNRNPK	Heterogeneous	Q5T6W5	50944	0	0
HNRNPL	Heterogeneous	M0QXS5	64092	0	0
HNRNPM	Heterogeneous	P52272	77464	847	27
HNRNPR	Heterogeneous	S4R3J4	70899	354	8
HNRNPU	Heterogeneous	Q00839	90528	19	0
HNRNPUL1	Heterogeneous	M0QYI8	95679	0	0
HNRNPUL2	Heterogeneous	Q1KMD3	85052	574	19
HOXB9	Homeobox pro	P17482	28041	0	0
HP1BP3	Heterochromat	Q5SSJ5	61169	0	0
HPRT1	Hypoxanthine-	P00492	24564	0	0
HRNR	Hornerin OS=H	Q86YZ3	282228	0	0
HSD17B10	3-hydroxyacyl-	Q5H928	26906	0	0
HSD17B12	Estradiol 17-be	Q53GQ0	34302	0	0
HSD17B4	Peroxisomal m	E7ER27	53894	0	0
HSP90AA1	Full-length cDN	Q86U12	84607	0	0
HSP90AB1	Heat shock pro	P08238	83212	0	0
HSP90AB2P	Putative heat s	Q58FF8	44321	0	0
HSP90B1	Endoplasmin O	P14625	92411	0	0
HSPA1A	Heat shock 70	P08107	70009	0	0
HSPA1L	Heat shock 70	P34931	70331	0	0
HSPA4	Heat shock 70	P34932	94271	0	0
HSPA5	78 kDa glucose	P11021	72288	97	5
HSPA6	Heat shock 70	P17066	70984	46	0
HSPA8	Heat shock cog	E9PI65	70854	1019	27

HSPA9	Stress-70 prote	P38646	73635	429	25
HSPD1	60 kDa heat sh	P10809	61016	0	0
HSPE1	10 kDa heat sh	P61604	10925	0	0
HTATSF1	HIV Tat-specifi	Q5H918	27727	0	0
HYPK	Huntingtin-inte	J3QT56	9243	0	0
IARS	Isoleucine--tRI	J3KR24	131680	0	0
IGF2BP1	Insulin-like gro	Q9NZI8	63441	0	0
IGHG1	Ig gamma-1 ch	P01857	36083	32	1
IGHG2	Ig gamma-2 ch	P01859	35878	7	1
IGLC2	Ig lambda-2 ch	P0CG05	11287	0	0
IGLL5	Immunoglobuli	B9A064	23049	88	2
IK	Protein Red (F	D6REL4	24742	34	1
ILF2	Interleukin enh	Q12905	43035	633	17
ILF3	Interleukin enh	K7ELV3	95748	553	18
IMMT	Mitochondrial i	B9A067	78925	0	0
IMP3	U3 small nucle	Q9NV31	21837	0	0
IMP4	U3 small nucle	H0Y714	31926	0	0
ING5	Inhibitor of gro	Q8WYH8	27733	0	0
INO80	DNA helicase I	H0YMN5	127886	32	1
IQGAP2	Ras GTPase-ac	E7EWC2	160235	0	0
IREB2	Iron-responsive	P48200	104978	74	3
IRGC	Interferon-indu	Q6NXR0	50257	0	0
IRS4	Insulin recepto	O14654	133685	0	0
ITPRIPL2	Inositol 1,4,5-tı	Q3MIP1	58409	9	0
JAGN1	Protein jagunal	Q8N5M9	21111	0	0
JUP	Junction plakoؚ	P14923	81693	0	0
KATNAL2	Katanin p60 AT	K7EIJ8	38097	0	0
KBTBD3	Kelch repeat ai	G3V161	60637	1	0
KCNK1	Potassium cha	Q5T5E6	27513	0	0
KCTD6	BTB/POZ dom	Q8NC69	27592	0	0
KDELR1	ER lumen prote	Q8NBW7	24526	0	0
KHDRBS1	KH domain-coı	Q07666	48197	47	1
KHSRP	Far upstream e	M0R0I5	73070	0	0
KIAA0020	Pumilio domair	Q15397	73538	0	0
KIAA0408	Uncharacterize	H3BRB8	33371	31	1
KIAA1429	Protein virilizer	Q69YN4	201898	0	0
KIF5A	Kinesin heavy (	J3KNA1	107187	0	0
KLHL22	Kelch-like prot	B7Z2G1	55532	0	0
KM-PA-2	Ribosome biog	Q96Q25	71885	0	0

KPNB1	Importin subun	Q14974	97108	0	0
KRR1	KRR1 small sul	Q13601	43638	0	0
KTN1	Kinectin OS=H	Q86UP2	156179	0	0
LAMA4	Laminin subun	H0YAP9	20794	0	0
LAMA5	Laminin subun	O15230	399479	32	1
LANCL1	LanC-like prot	F8WDS9	9277	41	1
LAS1L	Ribosomal biog	Q9Y4W2	83013	0	0
LBR	Lamin-B recep	Q14739	70658	0	0
LDHA	L-lactate dehy	F5GXY2	36665	0	0
LDHAL6B	L-lactate dehy	Q9BYZ2	41916	0	0
LDHB	L-lactate dehy	A8MW50	36615	0	0
LEMD2	LEM domain-c	D6R958	5903	0	0
LEPRE1	Prolyl 3-hydrox	Q32P28	83341	517	10
LEPREL4	Synaptonemal	K7EJ03	9536	0	0
LGALS3BP	Galectin-3-bin	Q08380	65289	0	0
LGI1	Leucine-rich gl	O95970	63777	0	0
LIG3	DNA ligase 3 C	P49916	112835	0	0
LIMA1	LIM domain an	Q9UHB6	85173	0	0
LIMCH1	LIM and calpor	H0Y8P3	103438	0	0
LINC00479	Putative uncha	Q96M42	15199	0	0
LLPH	Protein LLP ho	Q9BRT6	15215	0	0
LMNA	Prelamin-A/C (	P02545	74095	74	2
LMNB1	Lamin-B1 OS=	P20700	66368	1103	21
LMNB2	Lamin B2, isof	J9JID7	69906	232	5
LOC646903	LOC646903 pr	Q96EG4	15828	0	0
LRMP	Processed lym	F5H006	50394	3	0
LRPPRC	Leucine-rich P	P42704	157805	0	0
LRRC27	Leucine-rich re	H0Y4N5	29196	0	0
LRRC59	Leucine-rich re	Q96AG4	34909	0	0
LSM2	U6 snRNA-ass	Q9Y333	10828	0	0
LSM3	U6 snRNA-ass	P62310	11838	0	0
LSM4	U6 snRNA-ass	U3KQK1	13796	0	0
LSM6	U6 snRNA-ass	P62312	9122	0	0
LSM7	U6 snRNA-ass	K7EML7	6934	0	0
LUC7L2	Putative RNA-l	Q9Y383	46486	0	0
LUC7L3	Cisplatin resist	J3KPP4	58185	127	4
LYAR	Cell growth-reg	Q9NX58	43588	0	0
MACF1	Microtubule-ac	H3BPE1	856346	0	0
MAGEC1	MAGEC1 prote	A0PK03	23732	0	0

MAGOH	Mago-nashi ho	B1ARP8	12836	0	0
MAGOHB	Protein mago r	Q96A72	17265	0	0
MAK16	Protein MAK16	H0YBV6	16653	0	0
MAP1A	MAP1 light cha	J3KPX8	305484	0	0
MAP1B	Microtubule-as	P46821	270468	116	3
MAP4	Microtubule-as	E7EVA0	245292	0	0
MAPRE1	Microtubule-as	Q15691	29980	0	0
MAPRE2	Microtubule-as	K7EL66	15026	0	0
MATR3	Matrin-3 (Frag	D6R991	88305	257	6
MBD3	Methyl-CpG bi	K7EIE8	26306	0	0
MCM3	DNA replicatio	B4DWW4	95848	0	0
MCM5	DNA replicatio	B1AHA9	25928	0	0
MCOLN3	Mucolipin 3, is	B1ANB7	37219	0	0
MDC1	Mediator of DN	Q14676	226529	0	0
MDH2	Malate dehydro	G3XAL0	35481	0	0
MDN1	Midasin OS=H	Q9NU22	632420	538	13
MEAF6	Chromatin mod	Q9HAF1	21622	0	0
MEN1	Menin OS=Hor	E7EN32	61381	0	0
MERTK	Tyrosine-prote	E9PHX8	90964	0	0
MEX3A	RNA-binding p	A1L020	54139	42	1
MGST1	Microsomal glu	P10620	17587	0	0
MGST2	Microsomal glu	Q99735	16610	0	0
MGST3	Microsomal glu	Q5VV87	14370	0	0
MIF	Macrophage m	P14174	12468	0	0
MKI67	Antigen KI-67 (	P46013	358474	0	0
MOB1B	MOB kinase ac	Q7L9L4	25075	0	0
MOB2	MOB kinase ac	Q70IA6	26909	0	0
MOGS	Mannosyl-oligo	Q13724	91861	52	1
MOV10	Mov10, Molone	Q5JR04	107142	70	1
MPDU1	Mannose-P-do	I3L1D2	6475	0	0
MPHOSPH10	U3 small nucle	O00566	78816	0	0
MPHOSPH6	M-phase phos	H3BNK8	6732	0	0
MRE11A	Double-strand	B3KTC7	81020	0	0
MRPL10	39S ribosomal	Q7Z7H8	29264	0	0
MRPL11	39S ribosomal	Q9Y3B7	20670	0	0
MRPL17	39S ribosomal	E9PKV2	16361	0	0
MRPL21	39S ribosomal	H3BUY0	21513	0	0
MRPL22	39S ribosomal	J3KQY1	26458	35	1
MRPL23	39S ribosomal	A6NJD9	18518	0	0

MRPL38	39S ribosomal	B3KN96	22957	0	0
MRPS10	28S ribosomal	P82664	22985	0	0
MRPS16	28S ribosomal	B4E032	13820	0	0
MRPS17	28S ribosomal	I3L0E3	25704	0	0
MRPS2	28S ribosomal	Q5T8A0	30460	0	0
MRPS21	28S ribosomal	P82921	10734	0	0
MRPS23	28S ribosomal	J3QLR8	17506	0	0
MRPS28	28S ribosomal	Q9Y2Q9	20830	0	0
MRPS35	28S ribosomal	P82673	36821	51	1
MRTO4	mRNA turnover	Q9UKD2	27543	0	0
MSH2	DNA mismatch	E9PHA6	103122	0	0
MSH6	DNA mismatch	P52701	152689	144	4
MSN	Moesin OS=Ho	P26038	67778	0	0
MT-CO2	Cytochrome c o	P00403	25548	0	0
MTA1	Metastasis-ass	E7ESY4	79323	0	0
MTA2	Metastasis-ass	O94776	74976	0	0
MTHFD1	C-1-tetrahydro	F5H2F4	110544	0	0
MTPN	Myotrophin OS	C9JL85	5701	0	0
MXRA8	Matrix-remode	B3KTR6	47942	0	0
MYBBP1A	Myb-binding pr	Q9BQG0	148762	154	3
MYCBP	C-Myc-binding	Q99417	11959	0	0
MYEF2	Myelin express	Q9P2K5	64081	117	2
MYH10	Myosin-10 OS=	F8W6L6	230635	33	1
MYH11	Myosin-11 OS=	P35749	227199	0	0
MYH14	Myosin-14 OS=	F2Z2U8	231075	0	0
MYH7	Myosin-7 OS=H	P12883	222959	32	1
MYH9	Myosin-9 OS=H	P35579	226392	0	0
MYL12A	Myosin regulat	J3QRS3	20444	0	0
MYL6	Myosin light po	G3V1V0	18026	0	0
MYO1B	Unconventiona	E9PDF6	128400	0	0
MYO1C	Unconventiona	F5H6E2	118920	0	0
MYO1D	Unconventiona	J3QRN6	111219	0	0
NAA38	N-alpha-acetyl	F2Z2Y6	7367	0	0
NACA	Nascent polype	H0YHX9	22930	0	0
NAP1L1	Nucleosome as	H0YH88	21065	0	0
NASP	Nuclear autoar	P49321	85186	0	0
NAT10	N-acetyltransfe	Q9H0A0	115657	0	0
NAT14	N-acetyltransfe	M0R1E3	17831	0	0
NCBP1	Nuclear cap-bi	Q09161	91781	0	0

NCBP2	Nuclear cap-bi	F8WE41	6195	0	0
NCL	Nucleolin OS=I	P19338	76568	0	0
NCOA5	Nuclear recept	Q9HCD5	65496	0	0
NDUFA4	NADH dehydro	O00483	9364	0	0
NDUFA6	NADH dehydro	R4GN43	8504	0	0
NDUFS3	NADH dehydro	O75489	30223	0	0
NDUFS7	NADH dehydro	B3KRI2	22188	0	0
NEDD8	NEDD8 OS=Hc	Q15843	9066	0	0
NENF	Neudesin OS=	Q9UMX5	18845	0	0
NGDN	Neuroguidin (F	H0YJ17	30040	0	0
NHP2	H/ACA ribonuc	Q9NX24	17190	0	0
NHP2L1	NHP2-like prot	P55769	14165	0	0
NIFK	MKI67 FHA do	Q9BYG3	34201	0	0
NIP7	60S ribosome s	Q9Y221	20450	0	0
NKRF	NF-kappa-B-re	O15226	77624	199	4
NLE1	Notchless prot	K7ERN7	13709	0	0
NME1	Nucleoside dip	P15531	17138	0	0
NME1-NME2	Nucleoside dip	F6XY72	32621	0	0
NME2	Nucleoside dip	P22392	17287	0	0
NME3	Nucleoside dip	Q13232	19003	182	4
NME4	Nucleoside dip	F2Z2X0	25444	31	1
NMNAT1	Nicotinamide n	Q9HAN9	31913	0	0
NO66	Bifunctional lys	Q9H6W3	71041	0	0
NOC2L	Nucleolar comp	Q9Y3T9	84866	0	0
NOC3L	Nucleolar comp	A6NJZ9	92566	0	0
NOC4L	Nucleolar comp	Q9BVI4	58431	0	0
NOL10	Nucleolar prote	F5H6G7	74336	0	0
NOL11	Nucleolar prote	Q9H8H0	81072	0	0
NOL6	Nucleolar prote	G8JLK7	121957	0	0
NOL7	Nucleolar prote	Q9UMY1	29409	0	0
NOL9	Polynucleotide	Q5SY16	79272	0	0
NOLC1	Nucleolar and	Q14978	73560	0	0
NOM1	Nucleolar MIF4	Q5C9Z4	96198	0	0
NONO	Non-POU dom	C9IZL7	54197	0	0
NOP10	H/ACA ribonuc	Q9NPE3	7701	0	0
NOP14	Nucleolar prote	E9PFK5	88810	0	0
NOP16	Nucleolar prote	Q9Y3C1	21175	0	0
NOP2	Putative riboso	P46087	89247	0	0
NOP56	Nucleolar prote	O00567	66009	0	0

NOP58	Nucleolar prote	Q9Y2X3	59541	0	0
NPEPPS	Puromycin-sen	B7Z463	94456	0	0
NPM1	Nucleophosmir	P06748	32555	0	0
NPM3	Nucleoplasmin	O75607	19331	0	0
NSA2	Ribosome biog	O95478	30047	0	0
NTMT1	N-terminal Xaa	Q9BV86	25371	8	0
NUDT16L1	Protein syndes	Q9BRJ7	23323	64	2
NUDT21	Cleavage and p	H3BND3	26211	574	17
NUMA1	Isoform 2 of Nu	Q14980-2	238115	105	3
NUP107	Nuclear pore c	B4DZ67	103109	0	0
NUP133	Nuclear pore c	F5H5C2	127262	0	0
NUP153	Nuclear pore c	F6QR24	157240	0	0
NUP155	Nuclear pore c	E9PF10	148000	0	0
NUP160	Nuclear pore c	Q12769	162017	0	0
NUP188	Nucleoporin NU	Q5SRE5	195917	0	0
NUP205	Nuclear pore c	Q92621	227776	107	2
NUP210	Nuclear pore m	Q8TEM1	204983	75	2
NUP214	Nuclear pore c	P35658	213488	0	0
NUP35	Nucleoporin NU	B4DYB4	33099	0	0
NUP43	Nucleoporin Nu	Q8NFH3	42124	0	0
NUP62	Nuclear pore g	M0QXN5	45588	0	0
NUP85	Nuclear pore c	J3KT10	70133	0	0
NUP88	Nuclear pore c	J3KMX1	78831	0	0
NUP93	Nuclear pore c	Q8N1F7	93430	0	0
NUP98	Nuclear pore c	J3KP29	177290	0	0
NUPL1	Nucleoporin p5	Q5JRG1	49065	0	0
NVL	Nuclear valosir	O15381	94991	0	0
NXF1	Nuclear RNA e	E9PIN3	68065	0	0
OGT	UDP-N-acetylg	O15294	116850	0	0
OLA1	Obg-like ATPa:	J3KQ32	46908	0	0
ORC5	Origin recognit	O43913	50251	0	0
OTUD4	OTU domain co	G3V0I6	123968	0	0
P4HB	Uncharacterize	F5H8J2	57081	0	0
PA2G4	Proliferation-a:	F8VR77	43759	0	0
PABPC1	Polyadenylate-	H0YAP2	65706	0	0
PAFAH1B2	Platelet-activa	J3KNE3	18345	0	0
PAK1IP1	p21-activated p	Q9NWT1	43936	0	0
PAN3	Isoform 2 of PA	Q58A45-2	64424	0	0
PARK7	Protein DJ-1 O	K7ELW0	17898	0	0

PARP1	Poly [ADP-ribo	P09874	113012	0	0
PAXBP1	PAX3- and PA)	Q9Y5B6	104739	0	0
PBRM1	Protein polybrc	E7EVG2	168399	33	1
PCBD1	Pterin-4-alpha-	P61457	11992	0	0
PCBP1	Poly(rC)-bindir	Q15365	37474	100	2
PCBP2	Poly(RC) bindi	G3V0E8	33475	0	0
PCID2	PCI domain-co	Q5JVF3	46000	0	0
PCMT1	Protein-L-isoa:	J3KP72	30296	0	0
PCNA	Proliferating ce	P12004	28750	0	0
PCNP	PEST proteolyt	Q8WW12	18913	565	23
PCNXL4	Pecanex-like p	H0YJN3	13206	0	0
PDCD11	Protein RRP5 I	Q14690	208570	0	0
PDCD5	Programmed c	B4DE64	14988	0	0
PDCD6	Programmed c	O75340	21855	242	5
PDHB	Pyruvate dehyc	F8WF02	27633	0	0
PDIA3	Thioredoxin (F	H7BZJ3	54929	0	0
PDIA4	Protein disulfic	P13667	72887	0	0
PDIA6	Protein disulfic	B5MCQ5	53228	0	0
PDS5B	Sister chromati	Q9NTI5	164563	0	0
PEBP1	Phosphatidylet	P30086	21044	0	0
PEF1	Peflin OS=Hon	Q9UBV8	30361	0	0
PELP1	Proline-, glutar	I3L3A8	124865	55	1
PES1	Pescadillo hon	B5MCF9	66036	0	0
PEX6	Peroxin Pex6p	Q8WYQ0	77694	12	0
PFDN2	Prefoldin subu	Q9UHV9	16638	0	0
PFKFB3	6-phosphofruc	H0Y483	52551	0	0
PFN1	Profilin-1 OS=I	P07737	15045	0	0
PFN2	Profilin OS=Hc	G5E9Q6	20773	0	0
PGAM1	Phosphoglycer	P18669	28786	0	0
PGAM2	Phosphoglycer	P15259	28748	0	0
PGAM5	Serine/threoni	Q96HS1	31985	0	0
PGK1	Phosphoglycer	B7Z7A9	41402	0	0
PGRMC1	Membrane-ass	B7Z1L3	15869	0	0
PHB	Prohibitin (Fra	E7ESE2	26875	0	0
PHB2	Prohibitin-2 OS	J3KPX7	33382	0	0
PHF5A	PHD finger-like	Q7RTV0	12397	155	41
PHGDH	D-3-phosphogl	Q5SZU1	53051	0	0
PHIP	PH-interacting	Q8WWQ0	206560	0	0
PIN1	Peptidyl-prolyl	K7EN45	9938	0	0

PIN4	Peptidyl-prolyl	Q9Y237	13801	0	0
PIP	Prolactin-induc	P12273	16562	0	0
PKM	Pyruvate kinas	H3BQ34	30701	0	0
PLEC	Isoform 2 of Pl	Q15149-2	531466	0	0
PLOD1	Procollagen-lys	B4DR87	88217	416	7
PLOD3	Procollagen-lys	O60568	84731	155	3
PLRG1	Pleiotropic regu	A8MW61	57146	0	0
PNN	Pinin OS=Hom	Q9H307	81565	0	0
PNO1	RNA-binding p	Q9NRX1	27907	0	0
POFUT2	GDP-fucose pr	Q9Y2G5	49944	0	0
POGZ	Pogo transposa	Q7Z3K3	155245	0	0
POLDIP3	Polymerase de	B4DMM2	20211	0	0
POLG2	DNA polymeras	Q9UHN1	54876	26	1
POLR2A	DNA-directed I	P24928	217039	68	1
POLR2B	DNA-directed I	C9J2Y9	132971	0	0
POLR2C	DNA-directed I	P19387	31422	42	1
POLR2D	DNA-directed I	E9PB93	12259	0	0
POLR2E	DNA-directed I	P19388	24536	0	0
POLR2H	DNA-directed I	C9JBJ6	12950	61	1
POLR2K	DNA-directed I	P53803	6999	0	0
POLR2L	DNA-directed I	P62875	7640	0	0
PPA1	Inorganic pyrop	Q5SQT6	19969	0	0
PPA2	Inorganic pyrop	H0Y9D8	25619	0	0
PPAN	HCG2033702, i	A8MV53	47116	0	0
PPHLN1	Periphilin-1 (Fr	F8VPY7	19327	0	0
PPIA	Peptidyl-prolyl	F8WE65	18001	0	0
PPIB	Peptidyl-prolyl	P23284	23728	61	2
PPIF	Peptidyl-prolyl	P30405	22026	0	0
PPIH	Peptidyl-prolyl	C9JQD4	15790	39	1
PPIL1	Peptidyl-prolyl	Q9Y3C6	18225	0	0
PPP1CA	Serine/threoni	E9PMD7	28879	0	0
PPP1CC	Serine/threoni	F8VYE8	34927	0	0
PPP2R1A	Serine/threoni	B3KQV6	45575	0	0
PPP2R2A	Serine/threoni	E5RFR9	22992	0	0
PRADC1	Protease-asso	Q9BSG0	21029	0	0
PRAF2	PRA1 family pr	A6NP52	19246	0	0
PRC1	Protein regulat	H0YL53	23061	3	1
PRDX1	Peroxiredoxin-	Q06830	22096	0	0
PRDX2	Peroxiredoxin-:	P32119	21878	0	0

PRDX3	Thioredoxin-de	E9PH29	25822	0	0
PRDX4	Peroxiredoxin-4	H7C3T4	30521	0	0
PRDX5	Peroxiredoxin-!	P30044	22073	0	0
PRDX6	Peroxiredoxin-(	P30041	25019	0	0
PRKCSH	Glucosidase 2 :	K7EJ70	60154	0	0
PRKDC	DNA-depender	P78527	468788	534	10
PRMT1	Protein arginin	H0YDE4	27003	0	0
PRMT5	Protein arginin	O14744	72638	0	0
PRPF19	Pre-mRNA-pro	Q9UMS4	55146	79	1
PRPF3	U4/U6 small nı	B4DSY9	31869	30	1
PRPF31	U4/U6 small nı	E7ESA8	55421	0	0
PRPF39	Pre-mRNA-pro	Q86UA1	78381	221	5
PRPF4	U4/U6 small nı	O43172	58412	43	1
PRPF40A	Pre-mRNA-pro	O75400	108737	0	0
PRPF6	Pre-mRNA-pro	O94906	106858	161	3
PRPF8	Pre-mRNA-pro	Q6P2Q9	273427	1900	45
PRPS1	Ribose-phosph	B1ALA9	24068	0	0
PRSS3	Trypsin-3 (Frag	B1AN99	19389	0	0
PRSS33	Serine proteas	I3L3D7	25382	0	0
PSAP	Saposin-D OS=	B1AVU8	61652	0	0
PSIP1	PC4 and SFRS	O75475	60067	0	0
PSMA1	Proteasome su	F5GX11	26488	0	0
PSMA2	Proteasome su	H3BT36	5007	0	0
PSMA3	Proteasome su	P25788	28415	0	0
PSMA4	Proteasome su	H0YKS0	20062	0	0
PSMA5	Proteasome su	P28066	26394	0	0
PSMA8	Proteasome su	Q8TAA3	28512	0	0
PSMB1	Proteasome su	P20618	26472	0	0
PSMB5	Proteasome su	P28074	28462	0	0
PSMB6	Proteasome su	P28072	25341	0	0
PSMC1	26S protease r	B4DR63	41141	0	0
PSMD10	26S proteasom	B1AJY5	20204	0	0
PSMD8	26S proteasom	R4GMR5	32531	0	0
PSPC1	Paraspeckle cc	Q8WXF1	58706	0	0
PTBP1	Polypyrimidine	K7EK45	57186	0	0
PTBP2	Polypyrimidine	B4DI28	59583	0	0
PTBP3	Polypyrimidine	O95758	59652	0	0
PTGES3	Prostaglandin I	B4DDC6	14563	0	0
PTMA	Thymosin alpha	H7C2N1	15849	0	0

PTS	6-pyruvoyl tetr	E9PNN3	8908	0	0
PUF60	Poly(U)-bindin	H0YEM1	35810	0	0
PWP2	Periodic trypto	Q15269	102387	0	0
PYCR1	Pyrroline-5-car	J3KQ22	26423	0	0
PYROXD2	Pyridine nucle	Q8N2H3	63027	0	0
QIL1	Protein QIL1 O	Q5XKP0	13079	0	0
QPCT	Glutaminyl-pep	Q16769	40851	0	0
RAB10	Ras-related pr	P61026	22527	0	0
RAB11A	Ras-related pr	H3BMH2	17657	0	0
RAB15	Ras-related pr	P59190	24375	22	0
RAB33B	Ras-related pr	Q9H082	25701	19	0
RAB35	Ras-related pr	F5H157	21201	22	0
RAB3GAP2	Rab3 GTPase-	Q9H2M9	155886	0	0
RAB4B	HCG1995540, i	Q6PIK3	13528	0	0
RAB5A	Ras-related pr	B4DJA5	22164	0	0
RAB5C	Ras-related pr	P51148	23468	0	0
RAB6A	Ras-related pr	H0YGL6	22746	19	0
RAB7A	Ras-related pr	C9IZZ0	13067	0	0
RAB8B	Ras-related pr	H0YMN7	10478	104	2
RAD50	DNA repair pro	Q92878	153797	0	0
RAD52	Isoform beta o	P43351-2	24524	0	0
RAE1	mRNA export f	P78406	40942	0	0
RALY	RNA-binding p	Q9UKM9	32444	89	2
RALYL	RNA-binding R	B3KT61	31055	0	0
RAN	GTP-binding n	H0YFC6	26799	0	0
RANBP1	Ran-specific G	C9JDM3	23296	0	0
RANBP2	E3 SUMO-prot	P49792	357974	148	3
RANGAP1	Ran GTPase-a	F8W7I9	57738	0	0
RAP1B	Ras-related pr	F5GWU8	15737	0	0
RB1CC1	RB1-inducible	Q8TDY2	182975	49	1
RBBP4	Histone-bindin	H0YCT5	47626	93	2
RBBP5	Retinoblastom	Q15291	59116	40	1
RBBP7	Histone-bindin	E9PC52	46909	0	0
RBM10	Isoform 3 of R	P98175-3	103469	1403	62
RBM12B	RNA-binding p	Q8IXT5	118030	0	0
RBM14	RNA-binding p	Q96PK6	69449	551	12
RBM15	Putative RNA-l	Q96T37	107124	92	2
RBM17	Splicing factor	Q5W010	44934	1190	75
RBM20	RNA-binding p	Q5T481	134274	0	0

RBM25	RNA-binding p	P49756	100124	0	0
RBM28	RNA-binding p	H7C5G8	21267	0	0
RBM3	Putative RNA-b	P98179	17160	0	0
RBM34	RNA-binding p	P42696	48535	0	0
RBM39	RNA-binding p	E1P5S2	40516	277	6
RBM4	RNA-binding p	E9PB51	26270	95	2
RBM45	RNA-binding p	Q8IUH3	53469	71	1
RBM5	RNA-binding p	C9J9P7	92097	1819	45
RBM6	RNA-binding p	B4DNY1	113859	182	5
RBM8A	RNA-binding p	Q9Y5S9	19877	0	0
RBMX	RNA-binding m	P38159	42306	353	21
RBMXL1	RNA binding m	Q96E39	42116	276	14
RCC1	Regulator of ch	C9JW69	39559	0	0
RCC2	Protein RCC2 (	Q9P258	56049	0	0
RCL1	RCL1 OS=Hom	Q5VZU1	40817	0	0
RCN1	Reticulocalbin-	B7Z1M1	33006	156	3
RCN2	Reticulocalbin-	H0YL43	17709	51	1
RCOR1	REST corepres	J3KN32	53295	0	0
RECQL	ATP-dependen	P46063	73410	0	0
REEP5	Receptor expre	E2QRG8	18109	0	0
REM1	GTP-binding pı	O75628	32927	0	0
REXO4	RNA exonuclea	Q9GZR2	46643	0	0
RFC1	Replication fac	P35251	128175	0	0
RFC2	Replication fac	P35250	39132	0	0
RFC4	Replication fac	C9JZI1	36854	0	0
RFC5	Replication fac	P40937	38472	0	0
RGPD3	RanBP2-like ar	J3KNE0	198270	0	0
RGPD4	RanBP2-like ar	J3KNY6	198072	0	0
RGS3	Regulator of G-	B3KUB2	66436	0	0
RHCE	Blood group Rh	H0YCJ8	8389	0	0
RHEB	GTP-binding pı	C9J931	8668	0	0
RHOC	Rho-related GT	Q5JR08	21566	0	0
RIF1	Telomere-asso	Q5UIP0	274294	0	0
RMDN1	Regulator of m	H0YBD9	18414	0	0
RNF123	E3 ubiquitin-pr	Q5XPI4	148420	0	0
RNF13	E3 ubiquitin-pr	C9J8T4	17918	0	0
RNF216	E3 ubiquitin-pr	Q9NWF9	99342	0	0
RNPS1	RNA-binding p	H3BMM9	31681	0	0
ROCK2	Rho-associate	D6REE7	50695	0	0

RP1	Oxygen-regulat	P56715	240511	36	1
RPA1	Replication pro	P27694	68095	0	0
RPA2	Replication pro	Q5TEJ7	19421	0	0
RPA3	Replication pro	B5MC59	9165	0	0
RPF2	Ribosome prod	Q9H7B2	35560	0	0
RPL10	60S ribosomal	F8W7C6	18580	0	0
RPL10A	60S ribosomal	P62906	24816	0	0
RPL10L	60S ribosomal	Q96L21	24503	0	0
RPL11	60S ribosomal	P62913	20240	0	0
RPL12	60S ribosomal	P30050	17808	0	0
RPL13	60S ribosomal	P26373	24247	174	4
RPL13A	60S ribosomal	P40429	23562	0	0
RPL14	60S ribosomal	P50914	23417	0	0
RPL15	60S ribosomal	P61313	24131	0	0
RPL17	60S ribosomal	J3KRX5	20158	121	3
RPL18	60S ribosomal	G3V203	18962	0	0
RPL18A	60S ribosomal	M0R1A7	18067	0	0
RPL19	Ribosomal prot	J3KTE4	23233	0	0
RPL21	60S ribosomal	M0R181	14140	0	0
RPL22	60S ribosomal	K7EJT5	10416	0	0
RPL23	60S ribosomal	J3KT29	14856	2	0
RPL23A	60S ribosomal	P62750	17792	102	2
RPL24	60S ribosomal	C9JNW5	17532	110	2
RPL26	60S ribosomal	P61254	17248	108	3
RPL26L1	60S ribosomal	E5RIT6	15197	0	0
RPL27	60S ribosomal	P61353	16460	183	5
RPL27A	60S ribosomal	E9PLL6	12194	49	1
RPL28	60S ribosomal	P46779	19060	67	2
RPL3	60S ribosomal	P39023	46080	0	0
RPL30	60S ribosomal	E5RI99	12648	128	4
RPL31	60S ribosomal	B7Z4C8	15109	17	0
RPL32	60S ribosomal	D3YTB1	15607	0	0
RPL34	60S ribosomal	P49207	13284	0	0
RPL35	60S ribosomal	F2Z388	10638	52	1
RPL35A	60S ribosomal	P18077	12530	0	0
RPL36	60S ribosomal	Q9Y3U8	12246	0	0
RPL36A	60S ribosomal	H0Y5B4	15291	0	0
RPL37A	60S ribosomal	P61513	10268	0	0
RPL38	60S ribosomal	J3KT73	7560	65	1

RPL4	60S ribosomal	P36578	47667	0	0
RPL5	60S ribosomal	P46777	34341	0	0
RPL6	60S ribosomal	Q02878	32708	42	1
RPL7	60S ribosomal	P18124	29207	0	0
RPL7A	60S ribosomal	P62424	29977	0	0
RPL7L1	60S ribosomal	Q6DKI1	28643	0	0
RPL8	60S ribosomal	P62917	28007	0	0
RPL9	60S ribosomal	H0Y9V9	21557	0	0
RPLP0	60S acidic ribo	P05388	34252	70	1
RPLP1	60S acidic ribo	P05386	11507	23	0
RPLP2	60S acidic ribo	P05387	11658	0	0
RPN1	Dolichyl-diphos	P04843	68527	0	0
RPN2	Dolichyl-diphos	F2Z3K5	17797	0	0
RPRD1B	Regulation of n	Q9NQG5	36877	0	0
RPS10	40S ribosomal	P46783	18886	5	0
RPS10-NUDT3	Protein RPS10-	S4R435	32576	0	0
RPS11	40S ribosomal	P62280	18419	189	4
RPS12	40S ribosomal	P25398	14505	0	0
RPS13	40S ribosomal	P62277	17212	190	5
RPS14	40S ribosomal	P62263	16263	23	0
RPS15	40S ribosomal	K7EJ78	17712	61	1
RPS15A	40S ribosomal	P62244	14830	52	1
RPS16	40S ribosomal	M0R210	14410	92	2
RPS17	40S ribosomal	H0YK46	21629	0	0
RPS17L	40S ribosomal	P0CW22	15540	54	1
RPS18	40S ribosomal	P62269	17708	261	5
RPS19	40S ribosomal	M0QXK4	16051	56	2
RPS19BP1	Active regulato	Q86WX3	15424	0	0
RPS2	40S ribosomal	P15880	31305	241	6
RPS20	40S ribosomal	P60866	13364	0	0
RPS21	40S ribosomal	Q8WVC2	8844	0	0
RPS23	40S ribosomal	P62266	15798	54	1
RPS24	40S ribosomal	E7ETK0	15187	0	0
RPS25	40S ribosomal	P62851	13734	2	0
RPS26	40S ribosomal	P62854	13007	0	0
RPS26P11	Putative 40S ri	Q5JNZ5	12994	31	1
RPS27L	40S ribosomal	H0YMV8	11338	43	1
RPS28	40S ribosomal	P62857	7836	0	0
RPS29	40S ribosomal	P62273	6672	0	0

RPS3	40S ribosomal	P23396	26671	0	0
RPS3A	40S ribosomal	P61247	29926	93	2
RPS4X	40S ribosomal	P62701	29579	17	1
RPS5	40S ribosomal	M0R0F0	22377	2	0
RPS6	40S ribosomal	P62753	28663	0	0
RPS7	40S ribosomal	P62081	22113	0	0
RPS8	40S ribosomal	Q5JR95	21866	0	0
RPS9	40S ribosomal	P46781	22578	47	1
RPSA	40S ribosomal	C9J9K3	29487	0	0
RPSAP58	40S ribosomal	A6NE09	32888	0	0
RRNAD1	Protein RRNAD	Q4VX71	29655	0	0
RRP1	Ribosomal RN/	P56182	52807	0	0
RRP12	RRP12-like pro	Q5JTH9	143611	0	0
RRP1B	Ribosomal RN/	Q14684	84375	0	0
RRP7A	Ribosomal RN/	Q9Y3A4	32314	0	0
RRP9	U3 small nucle	O43818	51809	0	0
RRS1	Ribosome biog	Q15050	41168	0	0
RSL1D1	Ribosomal L1 c	O76021	54939	0	0
RSL24D1	Probable ribos	Q9UHA3	19608	0	0
RTCB	tRNA-splicing l	Q9Y3I0	55175	230	5
RTN1	Reticulon OS=l	A8MT72	21848	37	1
RTN3	Reticulon-3 OS	O95197	112541	46	1
RTN4	Reticulon OS=l	F8W914	37121	0	0
RUFY2	RUN and FYVE	H0YD93	71390	36	1
RUVBL1	RuvB-like 1 OS	Q9Y265	50196	0	1
RUVBL2	RuvB-like 2 OS	B3KQ59	46277	48	1
RYR1	Ryanodine rece	M0R014	100290	32	1
S100A10	Protein S100-A	P60903	11196	0	0
S100A11	Protein S100-A	P31949	11733	0	0
S100A7	Protein S100-A	P31151	11464	0	0
S100A8	Protein S100-A	P05109	10828	0	0
S100A9	Protein S100-A	P06702	13234	0	0
SAFB	Scaffold attach	Q15424	102580	342	9
SAFB2	Scaffold attach	Q14151	107408	390	9
SAMM50	Sorting and ass	A8MZI2	28477	3	3
SAP18	Histone deacet	O00422	17550	241	6
SAR1A	GTP-binding pı	Q5SQT8	13653	0	0
SATB2	DNA-binding p	C9JR56	75988	0	0
SCAF8	Protein SCAF8	B7Z888	147254	0	0

SCGB1D2	Secretoglobin f	O95969	9918	0	0
SCP2	Non-specific li	P22307	58956	0	0
SDAD1	Protein SDA1 h	Q9NVU7	79820	0	0
SEC11A	SEC11-like 1 (S	H0YK72	18976	0	0
SEC16A	Protein transpo	J3KNL6	251738	34	1
SEC22B	Vesicle-traffick	O75396	24578	0	0
SEC23A	Protein transpo	F5H365	82916	0	0
SEC23B	Protein transpo	Q5QPE1	86424	87	1
SEC24C	Protein transpo	E7EP00	111914	0	0
SEC61B	Protein transpo	S4R3B5	9968	0	0
SEC61G	Protein transpo	P60059	7736	0	0
SEH1L	Nucleoporin SE	Q96EE3	39623	0	0
SENP3	Sentrin-specifi	J3KNH7	64841	0	0
15-Sep	15 kDa selenop	O60613	17780	0	0
SERPINH1	Serpin H1 (Fra	E9PIG2	17351	0	0
SET	Protein SET OS	Q01105	33469	0	0
SF1	Splicing factor	F8WEV5	80569	754	26
SF3A1	Splicing factor	F8WB66	88831	1598	106
SF3A2	Splicing factor	K7EMT0	49224	782	25
SF3A3	Splicing factor	E7EUT8	58812	1631	64
SF3B1	Splicing factor	O75533	145738	2799	225
SF3B14	Pre-mRNA bra	Q9Y3B4	14576	161	88
SF3B2	Splicing factor	E9PPJ0	100165	2018	106
SF3B3	Splicing factor	H3BMB0	135492	2113	195
SF3B4	Splicing factor	Q15427	44357	332	36
SF3B5	Splicing factor	Q9BWJ5	10129	263	29
SFN	14-3-3 protein	P31947	27757	0	0
SFPQ	Splicing factor,	P23246	76102	0	0
SFRS3	Serine/arginine	B4E241	14194	8	0
SFSWAP	Splicing factor,	Q12872	104758	90	2
SHMT2	Serine hydroxy	G3V2Y4	25660	0	0
SHROOM3	Protein Shroom	Q8TF72	216724	0	0
SIGMAR1	Sigma non-opi	Q99720	25112	0	0
SKIV2L2	Superkiller vira	F5H7E2	106746	0	0
SKP1	S-phase kinase	P63208	18708	0	0
SLC12A7	Solute carrier f	Q9Y666	119029	0	0
SLC22A5	Solute carrier f	H7C1R8	15573	34	1
SLC25A1	Tricarboxylate	P53007	33991	0	0
SLC25A10	Mitochondrial c	B4DLN1	48069	0	0

SLC25A11	Mitochondrial 2	I3L1P8	32162	0	0
SLC25A3	Phosphate carr	F8VVM2	36138	0	0
SLC25A5	ADP/ATP trans	P05141	32831	108	3
SLC25A6	ADP/ATP trans	P12236	32845	0	0
SLFN11	Schlafen family	Q7Z7L1	102770	0	0
SLIRP	SRA stem-loop	H0YJ40	10843	0	0
SMAD6	Isoform B of M	O43541-2	26219	0	0
SMARCA1	Probable globa	P28370	122527	0	0
SMARCA4	SMARCA4 isof	Q9HBD4	188031	553	13
SMARCA5	SWI/SNF-relat	O60264	121828	0	0
SMARCC1	SWI/SNF comp	Q92922	122790	0	0
SMARCC2	SWI/SNF comp	F8VXC8	136100	83	2
SMARCE1	SWI/SNF-relat	J3QKS7	33413	0	0
SMC1A	Structural mair	Q14683	143144	116	3
SMC1B	Structural mair	Q8NDV3	143818	0	0
SMC3	Structural mair	Q9UQE7	141454	0	0
SMC4	Structural mair	E9PD53	144365	0	0
SMCHD1	Structural mair	J3KTL8	155549	0	0
SMNDC1	Survival of mot	O75940	26694	140	2
SMU1	WD40 repeat-c	Q2TAY7	57507	71	2
SND1	Staphylococcal	Q7KZF4	101934	0	0
SNRNP200	U5 small nucle	O75643	244353	1944	40
SNRNP40	U5 small nucle	B4DQJ1	44488	0	0
SNRNP70	U1 small nucle	M0QYR1	51526	109	3
SNRPA	U1 small nucle	M0QXK2	31259	138	15
SNRPA1	U2 small nucle	P09661	28398	666	83
SNRPB2	U2 small nucle	P08579	25470	400	69
SNRPC	U1 small nucle	P09234	17381	72	1
SNRPD1	Small nuclear r	P62314	13273	103	23
SNRPD2	Small nuclear r	P62316	13518	354	46
SNRPD3	Small nuclear r	B4DJP7	13283	53	42
SNRPE	Small nuclear r	P62304	10797	181	52
SNRPF	Small nuclear r	P62306	9719	116	15
SNRPG	Small nuclear r	F5H013	8490	91	23
SNRPN	Small nuclear r	B3KVR1	25059	345	27
SNX12	Sorting nexin-1	Q9UMY4	19718	0	0
SNX9	Sorting nexin-9	Q9Y5X1	66550	0	0
SOD1	Superoxide dis	H7BYH4	15926	0	0
SOD2	Superoxide dis	B3KUK2	19718	0	0

SON	Protein SON O	P18583	263664	78	2
SP140L	Nuclear body p	H7BYP4	46190	39	1
SPIN1	Spindlin-1 OS=	Q9Y657	29582	0	0
SPIN2B	Spindlin-2B (F	Q5JZB8	29140	0	0
SPTAN1	Spectrin alpha	A6NG51	284772	0	0
SPTBN1	Spectrin beta c	Q01082	274439	0	0
SRCAP	Helicase SRCA	Q6ZRS2	343343	0	0
SRCRB4D	Scavenger rece	Q8WTU2	60779	43	1
SRP14	Signal recognit	H0YLA2	14561	0	0
SRP68	Signal recognit	F5H5Y3	60247	0	0
SRP72	Signal recognit	R4GNC1	24794	0	0
SRP9	Signal recognit	E9PE20	7663	0	0
SRPK1	SRSF protein k	H3BLV9	75992	0	0
SRPK2	SRSF protein k	H7C5L6	33320	0	0
SRPRB	Signal recognit	H7C4H2	17602	0	0
SRRM2	Serine/arginine	Q9UQ35	299438	0	0
SRRT	Serrate RNA ef	H7C3A1	56698	0	0
SRSF1	Serine/arginine	J3KTL2	28312	108	2
SRSF10	Serine/arginine	Q5JRI1	20900	27	0
SRSF2	Serine/arginine	J3KP15	15518	0	0
SRSF4	Serine/arginine	S4R2X6	17939	0	0
SRSF5	Serine/arginine	Q13243	31245	0	0
SRSF6	Serine/arginine	Q13247	39563	0	0
SRSF7	Serine/arginine	C9JAB2	26912	33	0
SRSF9	Serine/arginine	Q13242	25526	266	8
SSB	Lupus La prote	P05455	46808	0	0
SSBP1	Single-strande	E7EUY5	15703	0	0
SSR4	Translocon-ass	P51571	18987	0	0
SSRP1	FACT complex	Q08945	81024	44	1
STK17A	Serine/threoni	Q9UEE5	46529	2	0
STK38	Serine/threoni	Q15208	54155	0	0
STK38L	Serine/threoni	Q9Y2H1	53968	0	0
STMN1	Stathmin (Frag	A2A2D0	17292	0	0
STMN2	Stathmin OS=H	E5RGX5	19562	0	0
STOML2	Stomatin-like p	B4E1K7	33317	0	0
STRBP	Spermatid peri	Q96SI9	73606	55	4
STRN4	Striatin-4 (Frag	R4GN16	34684	0	0
SUB1	Activated RNA	P53999	14386	0	0
SUGP1	SURP and G-p	Q8IWZ8	72425	1610	37

SULT1A1	Sulfotransferas	H3BRY5	32604	0	0
SUN2	SUN domain-c	B0QY63	25384	0	0
SUPT16H	FACT complex	Q9Y5B9	119838	0	0
SURF4	Surfeit 4 OS=H	Q5T8U5	21114	0	0
SURF6	Surfeit locus pı	O75683	41426	0	0
SYMPK	Symplekin OS=	Q92797	141059	0	0
SYNCRIP	Heterogeneous	O60506	69560	450	9
TAF12	Transcription iı	Q16514	17913	32	1
TAF15	TATA-binding		Q92804	61793	0	12
TAGLN2	Transgelin-2 O	P37802	22377	0	0
TARDBP	TAR DNA-bind	B1AKP7	33441	42	1
TARS	Threonine--tRI	G3XAN9	72022	0	0
TAX1BP1	Tax1-binding p	B8ZZD4	93551	0	0
TBCA	Tubulin-specifi	E5RHG6	15053	0	0
TBL3	Transducin bet	Q12788	88978	0	0
TCEB1	Transcription e	R4GMY8	6963	43	1
TCEB2	Transcription e	I3L0M9	15592	7	0
TCERG1	Transcription e	O14776	123823	0	0
TCOF1	Treacle protein	J3KQ96	144040	35	1
TECR	Very-long-chai	B3KSQ1	37443	0	0
TEX10	Testis-express	Q9NXF1	105608	0	0
TFAM	Transcription f	H7BYN3	25662	0	0
TFCP2	Alpha-globin tr	F8VWL0	43906	0	0
TFG	Protein TFG (F	C9JJP5	20352	0	0
TFIP11	Tuftelin-intera	Q9UBB9	96758	0	0
THOC2	THO complex s	Q8NI27	182659	0	0
THOC6	THO complex s	Q86W42	37511	0	0
THRAP3	Thyroid hormoı	Q9Y2W1	108601	0	0
TIA1	Nucleolysin TI	C9JTN7	42808	0	0
TIAL1	Isoform 2 of Nı	Q01085-2	43421	0	0
TIMM13	Mitochondrial i	Q9Y5L4	10493	0	0
TIMM50	Mitochondrial i	M0R003	28204	73	1
TIMM8A	Mitochondrial i	O60220	10991	0	0
TKT	Transketolase	B4E022	62839	0	0
TLN2	Talin-2 (Fragm	H0YMT1	154631	0	0
TMCC3	Transmembran	G3V207	50143	0	0
TMCO1	Transmembran	J3QQY2	11365	0	0
TMED10	Transmembran	P49755	24960	0	0
TMED2	Transmembran	F5GX39	13622	0	0

TMED9	Transmembran	Q9BVK6	27260	0	0
TMEM109	Transmembran	Q9BVC6	26194	0	0
TMEM167A	Protein kish-A	Q8TBQ9	8054	0	0
TMEM189	HCG2044781 C	G3V2F7	42181	0	0
TMEM194A	Transmembran	G3V5K2	12019	0	0
TMEM200C	Transmembran	A6NKL6	63889	0	0
TMEM205	Transmembran	Q6UW68	21184	0	0
TMEM33	Transmembran	D6RAA6	25207	0	0
TMEM43	Transmembran	Q9BTV4	44847	0	0
TMEM97	Transmembran	J3KT68	20834	0	0
TMPO	Lamina-associ	P42167	75446	99	2
TMPRSS13	Transmembran	J3KQC6	63127	16	0
TNPO3	Transportin-3 (	C9J7E5	107978	100	3
TNRC6B	Isoform 2 of Tr	Q9UPQ9-1	182703	0	0
TNRC6C	Trinucleotide r	G3XAB8	179211	0	0
TOMM22	Mitochondrial i	Q9NS69	15512	0	0
TOMM40	Mitochondrial i	O96008	37869	0	0
TOMM5	Mitochondrial i	F8W8Z9	10969	0	0
TOP1	DNA topoisom	P11387	90669	0	0
TOP2A	DNA topoisom	P11388	174276	0	0
TOP2B	DNA topoisom	Q02880	183152	0	0
TOR1A	Torsin-1A OS=	O14656	37784	0	0
TP53	Cellular tumor	P04637	43625	0	0
TPI1	Triosephospha	U3KPZ0	30772	0	0
TPM1	Tropomyosin a	H0YK48	28563	0	0
TPM3	Tropomyosin 3	Q5VU58	33202	0	0
TPR	Nucleoprotein	P12270	267131	0	0
TPT1	Translationally	H0YCX0	9091	0	0
TRA2A	Transformer-2	Q13595	32669	98	2
TRA2B	Transformer-2	P62995	33646	134	3
TRAM1	Translocating (	G3XAN4	33414	0	0
TRANK1	TPR and ankyr	B7WP88	273309	31	1
TRAP1	Heat shock prc	I3L0K7	57184	0	0
TRAPPC3	Trafficking pro	A6NKE1	13335	0	0
TRDN	Triadin OS=Ho	Q5SWK9	80708	0	0
TRIM21	E3 ubiquitin-pr	F5H012	54047	4	0
TRIM23	E3 ubiquitin-pr	P36406	64025	0	0
TRIM28	Transcription i	M0R0K9	88493	0	0
TRIM46	Tripartite motif	F5GYK0	69510	0	0

TRIP12	E3 ubiquitin-pr	Q14669	220296	0	0
TRMT112	tRNA methyltra	J3KNG5	13567	0	0
TRNAU1AP	tRNA selenocy:	Q9NX07	32477	0	0
TRRAP	Transformatior	H0Y4W2	405650	0	0
TSPAN10	Tetraspanin-1(	Q9H1Z9	36475	0	0
TTN	Titin (Fragmen	H7C1P9	109167	0	0
TUBA1A	Tubulin alpha-:	Q71U36	50104	970	24
TUBA1C	Tubulin alpha-:	F5H5D3	57693	294	13
TUBA4B	Putative tubuli	Q9H853	27534	36	7
TUBAL3	Tubulin alpha c	A6NHL2	49877	0	0
TUBB	Tubulin beta ch	Q5JP53	47736	92	7
TUBB2B	Tubulin beta-2	Q9BVA1	49921	96	6
TUBB3	Tubulin beta-3	Q13509	50400	465	13
TUBB4B	Tubulin beta-4	P68371	49799	139	7
TUBB6	Tubulin beta-6	Q9BUF5	49825	35	4
TUFM	Elongation fact	P49411	49510	0	0
TXN	Thioredoxin OS	P10599	11730	0	0
TXNDC12	Thioredoxin do	O95881	19194	0	0
TXNDC17	Thioredoxin do	I3L0K2	8366	0	0
TXNL4A	Thioredoxin-lik	K7ESL1	16775	31	1
U2AF1	Splicing factor	Q01081	27854	200	6
U2AF2	Splicing factor	K7ENG2	53467	981	27
U2SURP	U2 snRNP-ass	O15042	118219	1908	81
UBA1	Ubiquitin-like r	Q5JRR9	117774	0	0
UBA52	Ubiquitin-60S r	P62987	14719	0	0
UBB	Ubiquitin (Frag	J3QS39	10463	110	3
UBE2I	SUMO-conjuga	B0QYN7	20444	0	0
UBE2L3	Ubiquitin-conju	P68036	17850	0	0
UBE2M	NEDD8-conjug	P61081	20887	0	0
UBE2NL	Putative ubiqui	Q5JXB2	17366	0	0
UBTF	Nucleolar trans	E9PKP7	87380	0	0
UCHL1	Ubiquitin carbc	D6R956	26823	0	0
UFC1	Ubiquitin-fold r	Q9Y3C8	19446	0	0
UFL1	E3 UFM1-prote	O94874	89540	0	0
UGGT1	UDP-glucose:g	Q9NYU2	177078	0	0
UGT2B15	UDP-glucuronc	P54855	60996	0	0
UQCR10	Cytochrome b-	Q9UDW1	7304	0	0
URB1	Nucleolar pre-r	O60287	254227	0	0
USMG5	Up-regulated d	Q96IX5	6453	0	0

USP17L24	Ubiquitin carbo	Q0WX57	59673	0	0
USP39	U4/U6.U5 tri-s	B9A018	62005	0	0
USP9X	Probable ubiqu	Q93008	292094	0	0
UTP11L	Probable U3 sr	Q9Y3A2	30428	0	0
UTP14A	U3 small nucle	F8WD00	82164	0	0
UTP15	U3 small nucle	H0Y8P4	61393	0	0
UTP18	U3 small nucle	Q9Y5J1	61964	0	0
UTP20	Small subunit p	O75691	318182	0	0
UTP3	Something abo	Q9NQZ2	54525	0	0
UTP6	U3 small nucle	Q9NYH9	70149	0	0
UTS2	Urotensin-2 OS	Q5H8X8	16266	0	0
VAC14	Protein VAC14	H3BN23	4527	0	0
VAPA	Vesicle-associa	Q9P0L0	27875	0	0
VAPB	Vesicle-associa	E5RK64	7796	0	0
VAT1	Synaptic vesicl	K7EJM4	3348	0	0
VCP	Transitional en	P55072	89266	0	0
VDAC1	Voltage-depen	P21796	30754	0	0
VDAC2	Voltage-depen	B4DKM5	27462	0	0
VDAC3	Voltage-depen	F5H740	30738	0	0
VIM	Vimentin OS=H	P08670	53619	693	14
VMA21	Vacuolar ATPa	Q3ZAQ7	11347	0	0
VPS35	Vacuolar protei	Q96QK1	91649	0	0
VTN	Vitronectin OS=	P04004	54271	0	0
WDR12	Ribosome biog	Q9GZL7	47678	0	0
WDR18	WD repeat-cor	U3KQC1	43321	0	0
WDR26	WD repeat-cor	H0Y9R3	33225	54	1
WDR3	WD repeat-cor	Q9UNX4	106032	0	0
WDR33	pre-mRNA 3~ (	Q9C0J8	145799	73	2
WDR36	WD repeat-cor	Q8NI36	105255	0	0
WDR43	WD repeat-cor	Q15061	74843	0	0
WDR46	WD repeat-cor	O15213	68029	0	0
WDR65	WD repeat-cor	H7C4D4	9380	34	1
WDR74	WD repeat-cor	Q6RFH5	42415	0	0
WDR75	WD repeat-cor	Q8IWA0	94438	0	0
WDR77	Methylosome p	H0Y711	31079	0	0
WNT16	Protein Wnt-16	Q9UBV4	40663	0	0
WRB	Tail-anchored		H7C384	9013	0	0
XP32	Skin-specific p	Q5T750	26219	33	1
XPNPEP3	Probable Xaa-P	Q9NQH7	56997	4	0

XPO1	Exportin-1 OS=	O14980	123306	0	0
XRCC1	DNA repair pro	F5H8D7	65924	0	0
XRCC5	X-ray repair cr(	P13010	82652	0	0
XRCC6	X-ray repair cr(	P12956	69799	31	0
XRN2	5~-3~ exoribor	B4DZC3	102351	0	0
YBX1	Nuclease-sens	H0Y449	41991	0	0
YEATS2	YEATS domain	H0Y6M6	21152	0	0
YIF1B	Protein YIF1B (	Q5BJH7	34413	0	0
YLPM1	YLP motif-cont	F8VU51	160641	0	0
YTHDC1	YTH domain-co	J3QR07	85536	0	0
YTHDC2	Probable ATP-	Q9H6S0	160147	0	0
YWHAB	14-3-3 protein	P31946	28065	16	1
YWHAE	14-3-3 protein	P62258	29155	0	0
YWHAG	14-3-3 protein	P61981	28285	213	5
YWHAH	14-3-3 protein	A2IDB2	28201	0	0
YWHAQ	14-3-3 protein	P27348	27747	0	0
YWHAZ	14-3-3 protein	B0AZS6	28019	229	6
ZADH2	Zinc-binding al	J3KTQ8	16158	37	2
ZBBX	Zinc finger B-b	F2Z370	87945	30	1
ZBTB46	Zinc finger and	Q86UZ6	64043	0	0
ZC3H3	Zinc finger CC(	Q8IXZ2	101878	0	0
ZMYND8	Protein kinase	Q2HXV1	126336	46	1
ZNF277	Zinc finger prot	G5E9M4	33831	0	0
ZNF326	DBIRD comple:	Q5BKZ1	65613	256	7
ZNF638	Zinc finger prot	Q14966	220488	0	0
ZSWIM8	Zinc finger SW	S4R393	192863	0	0

gene nane	protein score	group
ILF2	633	mRNA binding
SNRPD1	103	Sm
SNRPD2	354	
SNRPD3	53	
SNRPE	181	
SNRPF	116	
SNRPG	91	
SNRNP70	109	U1
SNRPA	138	
SNRPC	72	
PHF5A	155	U2
SF3A1	1598	
SF3A2	782	
SF3A3	1631	
SF3B1	2799	
SF3B14	161	
SF3B2	2018	
SF3B3	2113	
SF3B4	332	
SF3B5	263	
SNRPA1	666	
SNRPB2	400	
CHERP	1622	U2 rel
DDX46	31	
DHX15	1456	
DNAJC8	704	
RBM17	1190	
SMNDC1	140	
U2AF1	200	
U2AF2	981	
U2SURP	1908	
C19orf43	563	A
CCAR1	1961	
DDX17	192	
FUS	32	
RBM10	1403	

RBM39	277	
RBM5	1819	
SF1	754	
SUGP1	1610	
CD2BP2	49	U5
EFTUD2	145	
PRPF6	161	
PRPF8	1900	
SNRNP200	1944	
TXNL4A	31	
PPIH	39	U4/U6
PRPF3	30	
PRPF4	43	
HSPA8	1019	Prp19
PRPF19	79	
CRNKL1	79	Prp19 rel
IK	34	B
SMU1	71	
MATR3	257	C2
RBM4	95	
SRSF1	108	SR
SRSF10	27	
SRSF7	33	
SRSF9	266	
TRA2A	98	
TRA2B	134	
HNRNPA0	127	hnRNP
HNRNPA2B1	403	
HNRNPAB	78	
HNRNPC	373	
HNRNPD	87	
HNRNPF	54	
HNRNPH1	79	
HNRNPH3	232	
HNRNPM	847	
HNRNPR	354	
HNRNPU	19	
PCBP1	100	

RALY	89	
RBMX	353	
SYNCRIP	450	
C16orf80	207	MISC
CIRBP	20	
ILF3	553	
KHDRBS1	47	
ACIN1	33	EJC/TREX
SAP18	241	
A2M	3	
AARD	39	
ACSL1	32	
ACTB	56	
ACTC1	67	
ACTL6A	1	
ADAR	577	
AGO3	86	
AKAP8	204	
AKAP8L	144	
ALG13	32	
ANP32A	1	
ANP32B	32	
ANP32C	98	
APOBEC3C	90	
ARF6	69	
ARHGEF17	33	
ARL1	70	
ASAP2	4	
ATP6V1C1	31	
BAG4	43	
BLOC1S6	68	
C12orf23	47	
C14orf166	373	
C1QBP	82	
CALCOCO1	30	
CALU	31	
CCNK	31	
CEP85	31	

CHD4	308
CKAP5	14
CPA5	30
CPSF1	264
CPSF2	186
CPSF3	76
CPSF6	219
CPSF7	304
CPVL	292
CRBN	59
CRTAP	313
CTTNBP2	1
DAPP1	11
DDB1	132
DDX1	170
DDX21	46
DDX39	11
DDX3Y	140
DHX30	55
DNAJA3	44
DSG1	43
DYNC1H1	1706
DYNLL2	37
EEF1A1	44
ENY2	101
EP400	36
ERH	12
FAM124B	33
FAM188B	40
FAM98A	39
FARP2	37
FBL	164
FBLL1	86
FIP1L1	63
FLG	9
FNBP4	30
GCN1L1	141
GIPC2	19

GJA8	30
GRB2	71
GTF2I	12
H3F3A	102
HDAC2	48
HIST1H2AG	67
HIST1H2BN	86
HNRNPDL	27
HNRNPUL2	574
HSPA5	97
HSPA6	46
HSPA9	429
IGHG1	32
IGHG2	7
IGLL5	88
INO80	32
IREB2	74
ITPRIPL2	9
KBTBD3	1
KIAA0408	31
LAMA5	32
LANCL1	41
LEPRE1	517
LMNA	74
LMNB1	1103
LMNB2	232
LRMP	3
LUC7L3	127
MAP1B	116
MDN1	538
MEX3A	42
MOGS	52
MOV10	70
MRPL22	35
MRPS35	51
MSH6	144
MYBBP1A	154
MYEF2	117

MYH10	33	ungrouped
MYH7	32	
NKRF	199	
NME3	182	
NME4	31	
NTMT1	8	
NUDT16L1	64	
NUDT21	574	
NUMA1	105	
NUP205	107	
NUP210	75	
PBRM1	33	
PCNP	565	
PDCD6	242	
PELP1	55	
PEX6	12	
PLOD1	416	
PLOD3	155	
POLG2	26	
POLR2A	68	
POLR2C	42	
POLR2H	61	
PPIB	61	
PRC1	3	
PRKDC	534	
PRPF39	221	
RAB15	22	
RAB33B	19	
RAB35	22	
RAB6A	19	
RAB8B	104	
RANBP2	148	
RB1CC1	49	
RBBP4	93	
RBBP5	40	
RBM14	551	
RBM15	92	
RBM45	71	

RBM6	182
RBMXL1	276
RCN1	156
RCN2	51
RP1	36
RPL17	121
RPL26	108
RPS17L	54
RPS26P11	31
RPS5	2
RTCB	230
RTN1	37
RTN3	46
RUFY2	36
RUVBL2	48
RYR1	32
SAFB	342
SAFB2	390
SAMM50	3
SEC16A	34
SEC23B	87
SFRS3	8
SFSWAP	90
SLC22A5	34
SLC25A5	108
SMARCA4	553
SMARCC2	83
SMC1A	116
SNRPN	345
SON	78
SP140L	39
SRCRB4D	43
SSRP1	44
STK17A	2
STRBP	55
TAF12	32
TARDBP	42
TCEB1	43

TCEB2	7	
TCOF1	35	
TIMM50	73	
TMPO	99	
TMPRSS13	16	
TNPO3	100	
TRANK1	31	
TRIM21	4	
TUBA1A	970	
TUBA1C	294	
TUBA4B	36	
TUBB	92	
TUBB2B	96	
TUBB3	465	
TUBB4B	139	
TUBB6	35	
UBB	110	
VIM	693	
WDR26	54	
WDR33	73	
WDR65	34	
XP32	33	
XPNPEP3	4	
XRCC6	31	
YWHAB	16	
YWHAG	213	
YWHAZ	229	
ZADH2	37	
ZBBX	30	
ZMYND8	46	
ZNF326	256	

protein score

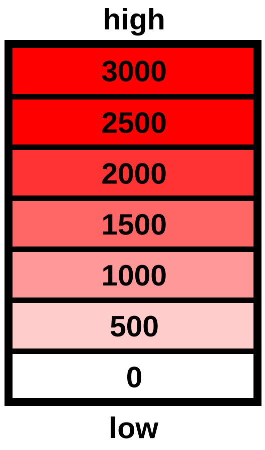

Supplement: Supplementary file 1 [file ijms-23-02555-s001.zip › Table S1.pdf]
