# Supplementary material for: CHERP Regulates the Alternative Splicing of pre-mRNAs in the Nucleus
Source: Int J Mol Sci. 2022 Feb 25;23(5):2555. doi: 10.3390/ijms23052555 (PMC8910253; doi:10.3390/ijms23052555)
Supplement: Supplementary file 1 [file ijms-23-02555-s001.zip › Table S2.pdf]

Name	Description
pcDNA5 FRT/TO	
pcDNA5 3×Flag FRT/TO	3×Flag inserted in the HindⅢ site pcDNA5 FRT/TO
pcDNA5 3×Flag CHERP FRT/TO	CHERP（coding sequence）, 2751bp<br>inserted in the Asp718Ⅰ/ EcoRⅤ site pcDNA5 3×Flag FRT/TO
pOG44	
pcDNA5 3×Flag RPGR_e14_e15 FRT/TO	RPGR from exon14 to exon15（including intron14）, 948bp<br>inserted in the BamHⅠ/ XhoⅠ site of pcDNA5 3×Flag CHERP FRT/TO
pcDNA5 3×Flag ARAP2_e23_e25 FRT/TO	ARAP2 from exon23 to exon25（including intron23 and intron24）, 4266bp<br>inserted in the BamHⅠ/ XhoⅠ site of pcDNA5 3×Flag CHERP FRT/TO
pcDNA5 3×Flag ATG16L1_e7_e9 FRT/TO	ATG16L1 from exon7 to exon9（including intron7 and intron8）, 1813bp<br>inserted in the BamHⅠ/ XhoⅠ site of pcDNA5 3×Flag CHERP FRT/TO
pcDNA5 3×Flag PPP1R12A_e12_e14 FRT/TO	PPP1R12A from exon12 to exon14（including intron12 and intron13）, 1739bp<br>inserted in the BamHⅠ/ XhoⅠ site of pcDNA5 3×Flag CHERP FRT/TO
pcDNA5 3×Flag SIK3_e8_e10 FRT/TO	SIK3 from exon8 to exon10（including intron8 and intron9）, 990bp<br>inserted in the BamHⅠ site of pcDNA5 3×Flag CHERP FRT/TO
