# Supplementary material for: CHERP Regulates the Alternative Splicing of pre-mRNAs in the Nucleus
Source: Int J Mol Sci. 2022 Feb 25;23(5):2555. doi: 10.3390/ijms23052555 (PMC8910253; doi:10.3390/ijms23052555)
Supplement: Supplementary file 1 [file ijms-23-02555-s001.zip › Table S3.pdf]

Name		Nucleotide sequence (5'→3')	Used in the
EGFP	Sense	GGGCACAAGCUGGAGUACAACUACA	(various studies)
	Antisense	UGUAGUUGUACUCCAGCUUGUGCCC	
CAT	Sense	CAAUGAAAGACGGUGAGCUGGUGAU	live cell imaging
	Antisense	(unpublished)	
CHERP#1	Sense	GAGAAGAGAACAAAGGCCAUCAGAU	RNA-FISH
	Antisense	(unpublished)	
CHERP#2	Sense	rGrGrUrUrUrArGrGrUrCrUrArGrArArArGrArArGrArArUAC	(various studies)
	Antisense	rGrUrArUrUrCrUrUrCrUrUrUrCrUrArGrArCrCrUrArArArCrCrArA	
UAP56	Sense	CCUGAUGAGAUAGACAUCUCCUCCU	RNA-FISH
	Antisense	(unpublished)	
NXT1	Sense	GAACUGGUUCAAGAUUACAAUUCCU	RNA-FISH
	Antisense	(unpublished)	
