# Supplementary material for: CHERP Regulates the Alternative Splicing of pre-mRNAs in the Nucleus
Source: Int J Mol Sci. 2022 Feb 25;23(5):2555. doi: 10.3390/ijms23052555 (PMC8910253; doi:10.3390/ijms23052555)
Supplement: Supplementary file 1 [file ijms-23-02555-s001.zip › Table S4.pdf]

Name		Nucleotide sequence (5'→3')	Used in the	
PGK1	Forward	GTTGCAGACAAGATCCAGCTC	quantification of mRNA expression	
	Reverse	GAAGTGCCAATCTCCATGTTG		
CHERP	Forward	CCAATGTGCCTACTTCGAT	quantification of mRNA expression	
	Reverse	TGTACTCGTGATCTTCCAGCTT		
	Forward	TGACAAGCTTGCATGCATGGAGATGCCGCTGC	construction of expression vector wild-type	
	Reverse	GCCACTGTGCTGGATCTACTTACACTCGTCCCTGG		
tRNA	Forward	GTAGTCGTGGCCGAGTGGTTAAG	confirmation of the fractionation	
	Reverse	GTAGTCGGCAGGATTCGAACC		
U6 snRNA	Forward	GTGCTCCCTTCGGCAAGCACTTACACTAAA	confirmation of the fractionation	
	Reverse	CTCAAAAAGGAATGCTTCACAAATTTGCCT		
E2F8	Forward	CGGGGAGGAGAATAAGTACG	confirmation of the fractionation	
	Reverse	CTTGTCTTTGCGGCTGTTTA		
CANT1	Forward	CCTTCCTCACTCAGCTTCC	quantification of increased intron inclusion	
	Reverse	GGTCAGGTAGCCCTTTTTCA		
RPGR	Forward	GACGCAGGATACAGCTCTTA	quantification of increased intron inclusion	
	Reverse	TTGAATCCTCTGCTCCTTCC		
	Forward	CGCGGATCCAAACAACAAACAATTGGGGAACTGA	construction of minigene wild-type	
	Reverse	ATATCCGCTCGAGCTCTGCTTTGTCTGTAAGGTCATCTGATA		
	Forward	AGATGAGGAAGCAGGTAAGGACACAGGCCA	construction of minigene mutant for 5' splice site	1st PCR
	Reverse	TGGCCTGTGTCCTTACCTGCTTCCTCATC		
	Forward	CGCGGATCCAAACAACAAACAATTGGGGAACTGA		2nd PCR
	Reverse	ATATCCGCTCGAGCTCTGCTTTGTCTGTAAGGTCATCTGATA		
	Forward	GTGATCTCTTTTCAGGTATCCCAGAGGAGAAGGAAGG	construction of minigene mutant for 3' splice site	1st PCR
	Reverse	CCTTCCTTCTCCTCTGGGATACCTGAAAAGAGATCAC		
	Forward	CGCGGATCCAAACAACAAACAATTGGGGAACTGA		2nd PCR
	Reverse	ATATCCGCTCGAGCTCTGCTTTGTCTGTAAGGTCATCTGATA		
RSRP1	Forward	GAACAACCAACATTGACTTGC	quantification of increased intron inclusion	
	Reverse	CTTTGCTGGGTAGGTTTTTC		
DVL3	Forward	TGAGCAGTGAGCTGGAGACC	quantification of decreased intron inclusion	
	Reverse	CGCCGCTTGTGTCTTCTCATC		
PODXL	Forward	ACCTACCCTGCCAGAGACCA	quantification of decreased intron inclusion	
	Reverse	AGGACGAGCTGCTTCTCACTC		
SUPT7L	Forward	TTTGACCGACACAGAATGCCTG	quantification of decreased intron inclusion	
	Reverse	AGTGTCAGCAAAACTGTAGGACCA		
ARAP2	Forward	TGGCCTTGGTCTTTTCATCC	quantification of exon inclusion	
	Reverse	CAGTCGGGTTCCTTCCTTTC		
ATG16L1	Forward	TTGAGGTCATTGTGGATGAAAC	quantification of exon inclusion	
	Reverse	ACTGGGAAGGAAGAGACAGA		
	Forward	CGCGGATCCGGATGATGACATTGAGGTCATTGTGGA	construction of minigene wild-type	
	Reverse	CCGCTCGAGGAAGACACACAAGGCAGTAG		
	Forward	CCTTTCCTCCCTCCCTTTTTAGTAAGCGACTCTCGC	construction of minigene mutant for 3' splice site	1st PCR
	Reverse	GCGAGAGTCGCTTACTAAAAAGGGAGGGAGGAAAGG		
	Forward	CCTATCCTCGCCTCCTCTTTAGTAAGCGACTCTCG		
	Reverse	CGAGAGTCGCTTACTAAAGAGGAGGCGAGGATAGG		
	Forward	CGCGGATCCGGATGATGACATTGAGGTCATTGTGGA		2nd PCR
	Reverse	CCGCTCGAGGAAGACACACAAGGCAGTAG		
	Forward	TTCTGGATTCTATCACTAATATCTTTGGGTAAGTTAGAAGACCTTTCCTTTTTAAAT	construction of minigene mutant for 5' splice site	1st PCR
	Reverse	CACATTTAAAAAGGAAAGGTCTTCTAACTTACCCAAAGATATTAGTGATAGAATCC		
	Forward	TTCTGGATTCTATCACTAATATCTTTGGGTAGGGTAGAAGACCTTTCCTTTTTAAA		
	Reverse	TACACATTTAAAAAGGAAAGGTCTTCTACCCTACCCAAAGATATTAGTGATAGAAT		
	Forward	CGCGGATCCGGATGATGACATTGAGGTCATTGTGGA		2nd PCR
	Reverse	CCGCTCGAGGAAGACACACAAGGCAGTAG		
CLASP1	Forward	TGCCATGAGAGTTCTGAGC	quantification of exon inclusion	
	Reverse	AACTAGCACAGTGGTTGAGA		
EFCAB14	Forward	ATCATTCCGTCACCTTCAGC	quantification of exon inclusion	
	Reverse	ACTGTCTTTTCAGAGTGGCAG		
INTS13	Forward	GAACAACCACGAAAGTCAGG	quantification of exon inclusion	
	Reverse	CCTCTTGTACCAACTGTGGA		
MAP4K2	Forward	TGACTTTGGGGTGTCAGG	quantification of exon inclusion	
	Reverse	CTTATCTCTCAGTTTGGGCG		
MON2	Forward	GCCATTTGTGTAGGACCAG	quantification of exon inclusion	
	Reverse	AATTCAAGGCTACCCATTCC		
SNX14	Forward	CAGAATCACCAACACGCAA	quantification of exon inclusion	
	Reverse	TCATCTTCACCTTCAGCTACA		
ZNF207	Forward	CCTCCAATGACTCAAGCACA	quantification of exon inclusion	
	Reverse	AATGTAGGCTTTGGGGGTTC		
ZFAND1	Forward	GTGTGTGATGATTGTTCAGG	quantification of exon inclusion	
	Reverse	TAACTGCCACAAGTTCTCTC		
AURKB	Forward	GGCGTGGCAGATTCAGTTG	quantification of exon skipping	
	Reverse	TGAGGACAAGTGCAGATGGG		
CDCA7L	Forward	CTTCCGAGATGATGTTCCCA	quantification of exon skipping	
	Reverse	GAAAGGCTACTCGAAGACCA		
CHFR	Forward	AAAGCAAGGCATGACACAAG	quantification of exon skipping	
	Reverse	AGACTTCATCACTTGCCACA		
FN1	Forward	TACCACAACCCCTACAAACG	quantification of exon skipping	
	Reverse	ACTGCATTGTCTGAAGGAGA		
KTN1	Forward	GAAAAGGCAGAGATGGAACG	quantification of exon skipping	
	Reverse	ATCACCAGCTACCTTCTGTC		
MYO1B	Forward	CATCAAAAGCGCTGTAAGGA	quantification of exon skipping	
	Reverse	CAAGCGTTTCAATTCCCTTC		
NUP50	Forward	AAGAAATGCCGAGAAGGAAC	quantification of exon skipping	
	Reverse	GCGCTTTGCTTTCTTTATGG		
NUP62	Forward	AAGAAAGAAGGTGGGAGCGG	quantification of exon skipping	
	Reverse	AGTGAAGCCTGTCGTCTGTG		
OGT	Forward	AGGCACGCATTTTTGACAG	quantification of exon skipping	
	Reverse	GCGAACTGCCTCTTCAATG		
PPP1R12A	Forward	AGCTCAGTTAATGAAGGATCAACG	quantification of exon skipping	
	Reverse	TCCCTGACCTCTGTTGTGGA		
RPP40	Forward	ACTTCAGGGTCATCCATCTC	quantification of exon skipping	
	Reverse	CTACTTTTGGCTGATGCTCC		
SIK3	Forward	GACATGGGACTGGACAAAGA	quantification of exon skipping	
	Reverse	ACAATTTGGTTCTCTGGGTTG		
	Forward	TACCGAGCTCGCTAGTTAATAGCTGAATGCCAACAACTAAAGGAAG	construction of minigene wild-type	
	Reverse	CTGGACTAGTGCTAGCTCCACAATTTGGTTCTCTGGGTTG		
	Forward	GTTTTGTACTCTTTTCCCcTCTCTCTTtGTAGTCATTAAGATC	construction of minigene mutant for 3' splice site	1st PCR
	Reverse	GATCTTAATGACTACaAAGAGAGAgGGGAAAAGAGTACAAAAC		
	Forward	CAGGGTTTTGTACTCTTTTCaCTaCTCTCTTGGTAGTCATTAAGATC		
	Reverse	GATCTTAATGACTACCAAGAGAGtAGtGAAAAGAGTACAAAACCCTG		
	Forward	TACCGAGCTCGCTAGTTAATAGCTGAATGCCAACAACTAAAGGAAG		2nd PCR
	Reverse	CTGGACTAGTGCTAGCTCCACAATTTGGTTCTCTGGGTTG		
	Forward	CACCAGTCAATATCCAGGTaGGtAATAACTCCAGTGACCTAG	construction of minigene mutant for 5' splice site	1st PCR
	Reverse	CTAGGTCACTGGAGTTATTaCCtACCTGGATATTGACTGGTG		
	Forward	GCACCAGTCAATATCCAGGTtGGCAATAACTCCAGTGACCTAG		
	Reverse	CTAGGTCACTGGAGTTATTGCCaACCTGGATATTGACTGGTGC		
	Forward	TACCGAGCTCGCTAGTTAATAGCTGAATGCCAACAACTAAAGGAAG		2nd PCR
	Reverse	CTGGACTAGTGCTAGCTCCACAATTTGGTTCTCTGGGTTG		
SKA3	Forward	GCGCCGAGATTCAAACTA	quantification of exon skipping	
	Reverse	TTTTCTTGACACGTGGACTA		
TBL1X	Forward	TTTTATGACCGGATGCTGGG	quantification of exon skipping	
	Reverse	ATGCTCATCTTAGCCTCACC		
pcDNA5 (-specific)	Forward	GTTTTGACCTCCATAGAAGACA	quantification of minigene reporter assay	
	Reverse	TAGAAGGCACAGTCGAGG		
BIRC5	Forward	TTGGCCCAGTGTTTCTTCTGC	quantification of mRNA expression	
	Reverse	TGACAGAAAGGAAAGCGCAACC		
AURKB	Forward	AGAAGCAATTGCAGGCAACCAG	quantification of mRNA expression	
	Reverse	GTTTTGATGCCAGTTCCTCCTCAG		
CENPA	Forward	AAGCTTCAGAAGAGCACACACC	quantification of mRNA expression	
	Reverse	TGCCAATTGAAGTCCACACCAC		
BRCA1	Forward	GTCCCATCTGTCTGGAGTTG	quantification of mRNA expression	
	Reverse	GCCCTTTCTTCTGGTTGAGA		
