# Supplementary figures and images for: CHERP Regulates the Alternative Splicing of pre-mRNAs in the Nucleus
Source: Int J Mol Sci. 2022 Feb 25;23(5):2555. doi: 10.3390/ijms23052555 (PMC8910253; doi:10.3390/ijms23052555)

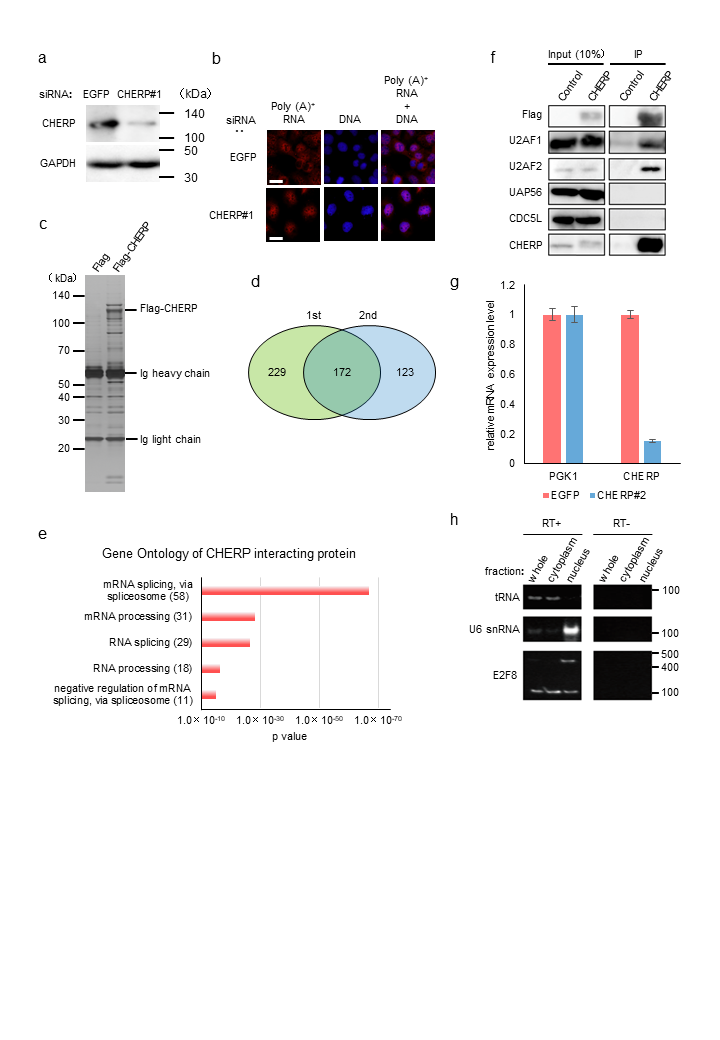

Supplement: Supplementary file 1 [file ijms-23-02555-s001.zip › Figure S1.tif]

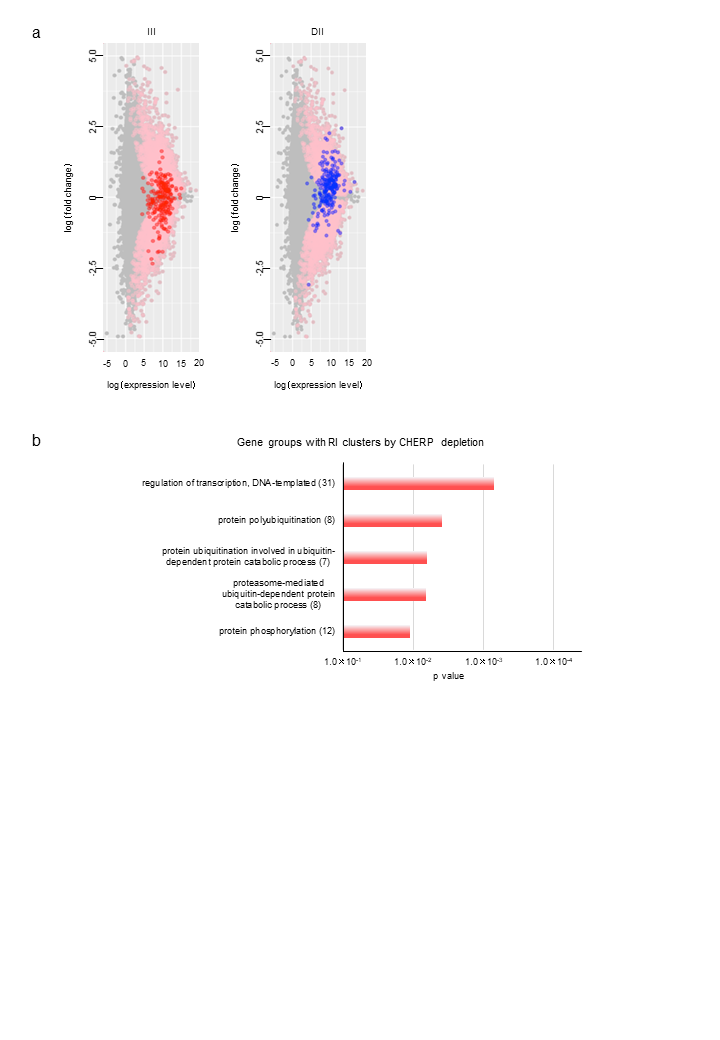

Supplement: Supplementary file 1 [file ijms-23-02555-s001.zip › Figure S2.tif]

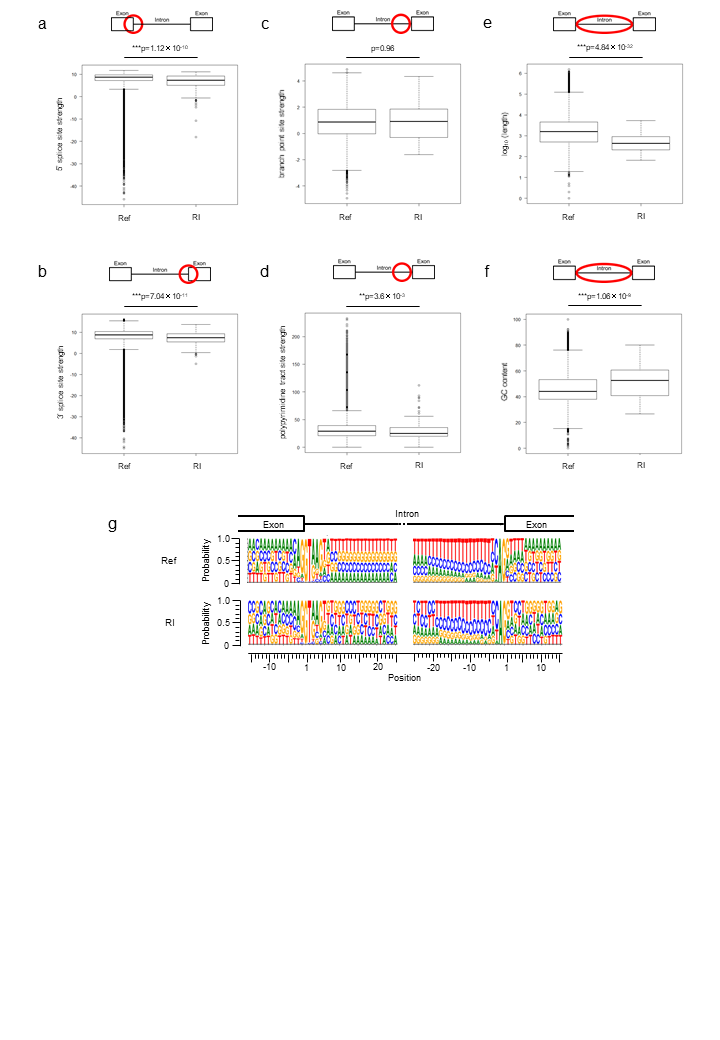

Supplement: Supplementary file 1 [file ijms-23-02555-s001.zip › Figure S3.tif]

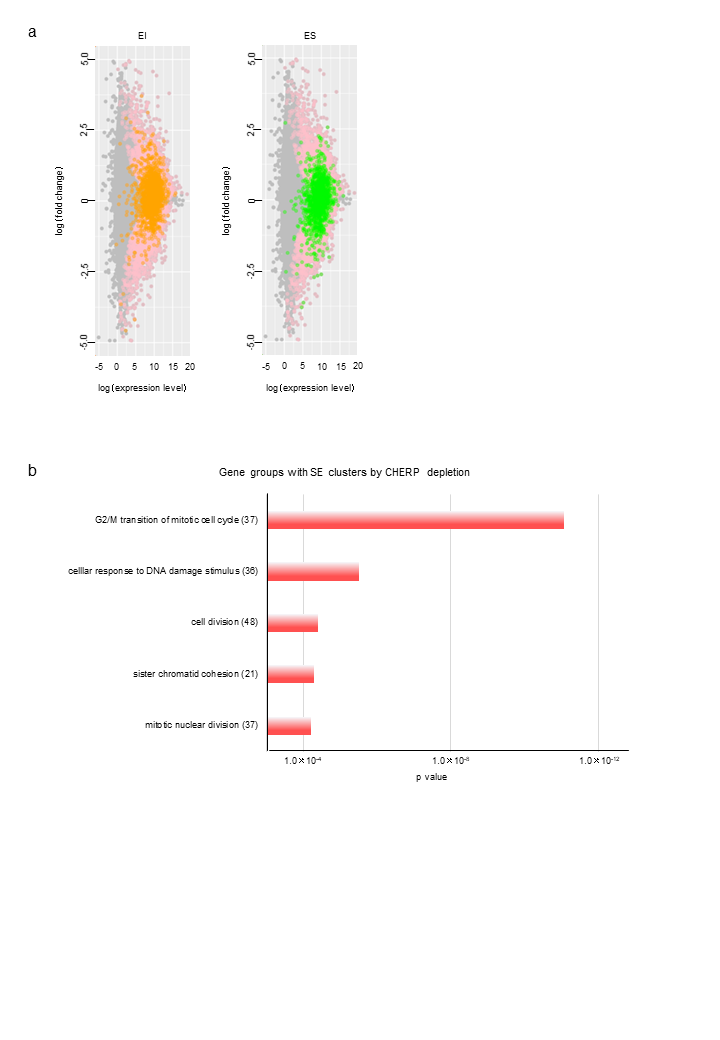

Supplement: Supplementary file 1 [file ijms-23-02555-s001.zip › Figure S4.tif]

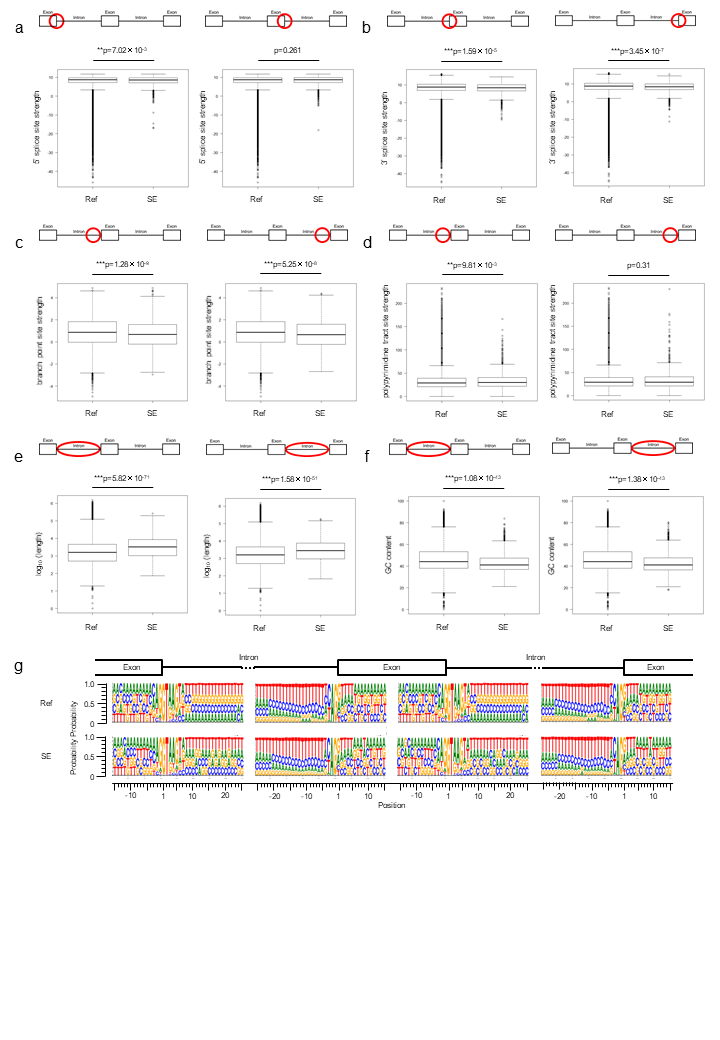

Supplement: Supplementary file 1 [file ijms-23-02555-s001.zip › Figure S5.tif]

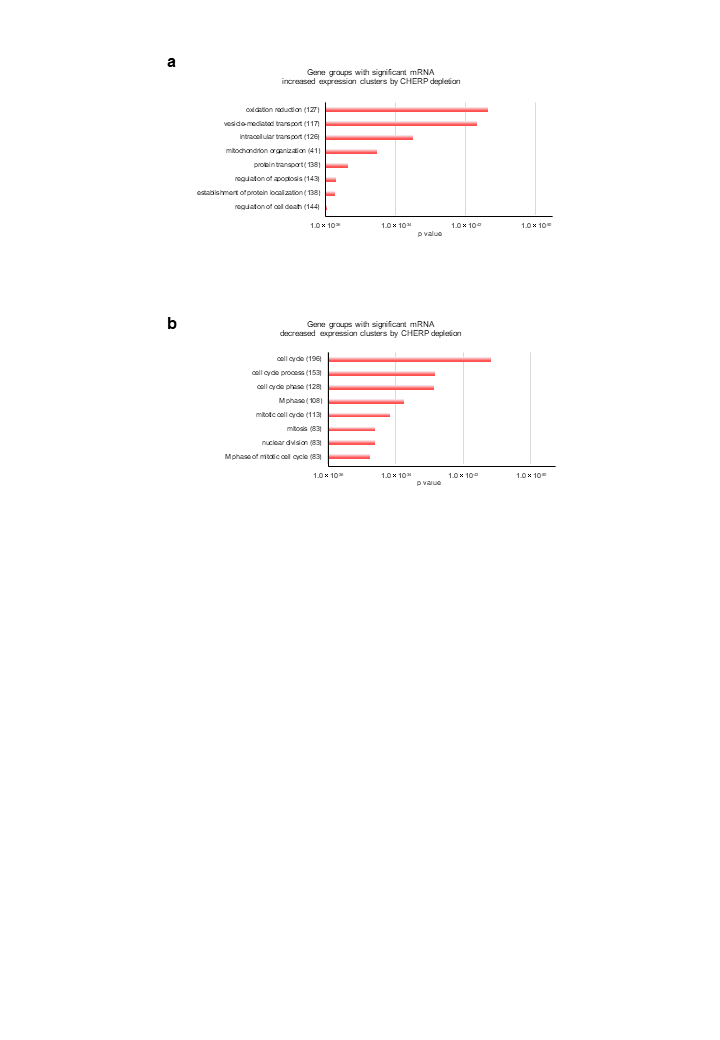

Supplement: Supplementary file 1 [file ijms-23-02555-s001.zip › Figure S6.tif]
